# The clinical presentation and detection of tuberculosis during pregnancy and in the postpartum period in low- and middle-income countries: A systematic review and meta-analysis

**DOI:** 10.1371/journal.pgph.0002222

**Published:** 2023-08-23

**Authors:** Grace Simpson, Moira Philip, Joshua P. Vogel, Michelle J. L. Scoullar, Stephen M. Graham, Alyce N. Wilson

**Affiliations:** 1 Maternal Child and Adolescent Health Program, International Development, Burnet Institute, Melbourne, Australia; 2 Centre for International Health, University of Melbourne Department of Paediatrics, Melbourne, Australia; Indian Institute of Public Health Gandhinagar, INDIA

## Abstract

For women infected with *Mycobacterium tuberculosis*, pregnancy is associated with an increased risk of developing or worsening TB disease. TB in pregnancy increases the risk of adverse maternal and neonatal outcomes, however the detection of TB in pregnancy is challenging. We aimed to identify and summarise the findings of studies regarding the clinical presentation and diagnosis of TB during pregnancy and the postpartum period (within 6 months of birth) in low-and middle-income countries (LMICs). A systematic review was conducted searching Ovid MEDLINE, Embase, CINAHL and Global Index Medicus databases. We included any primary research study of women diagnosed with TB during pregnancy or the postpartum period in LMICs that described the clinical presentation or method of diagnosis. Meta-analysis was used to determine pooled prevalence of TB clinical features and health outcomes, as well as detection method yield. Eighty-seven studies of 2,965 women from 27 countries were included. 70.4% of women were from South Africa or India and 44.7% were known to be HIV positive. For 1,833 women where TB type was reported, pulmonary TB was most common (79.6%). Most studies did not report the prevalence of presenting clinical features. Where reported, the most common were sputum production (73%) and cough (68%). Having a recent TB contact was found in 45% of women. Only six studies screened for TB using diagnostic testing for asymptomatic antenatal women and included mainly HIV-positive women ‒ 58% of women with bacteriologically confirmed TB did not report symptoms and only two were in HIV-negative women. Chest X-ray had the highest screening yield; 60% abnormal results of 3036 women tested. Screening pregnant women for TB-related symptoms and risk factors is important but detection yields are limited. Chest radiography and bacteriological detection methods can improve this, but procedures for optimal utilisation remain uncertain in this at-risk population.

**Trial registration:** *Prospero registration number*: CRD42020202493.

## Introduction

Tuberculosis (TB) has consistently been the major cause of death associated with infectious disease globally, at least until the COVID pandemic. An estimated 1.7 billion people are infected with *Mycobacterium tuberculosis* and therefore at risk of developing TB. Each year an estimated 10 million people develop TB and 1.5 million die [1, 2]. For women infected with *M*. *tuberculosis*, pregnancy is associated with an increased risk of developing or worsening TB, which can have major consequences for the health of the mother, fetus and infant [3–5]. In TB-endemic countries, TB is an important cause of maternal morbidity and mortality and is associated with an increased risk of preterm birth, low birth weight, and fetal death [6]. While congenital TB infection is itself rare, pregnancy-related TB is often not detected until the postnatal period, by which time newborns may have been exposed [7–9]. Early detection and treatment of TB in pregnant women are critical to reduce such risks.

Approximately 216,500 women developed TB during pregnancy in 2014, however the actual number of cases is likely higher [10]. TB in pregnancy is most common in those countries with the highest prevalence of TB infection in the community. Many of these countries are also low- and middle-income countries (LMICs), with limited resources for health care and surveillance [11]. For countries with a population TB prevalence of 100 cases per 100,000 people or greater, the World Health Organization (WHO) recommends considering screening for active TB in pregnant women as part of routine antenatal care [12]. This may be via standardised symptom screening or chest radiography [12]. However, symptom screening is problematic as TB symptoms in pregnancy are often difficult to detect. TB-associated weight loss may be masked by gestational weight gain, and TB symptoms such as fatigue, dyspnoea, mild fever and night sweats may be mistaken for pregnancy-related symptoms or physiological changes [8, 13]. Furthermore, the addition of a systematic screening approach to identify those with TB among all pregnant women in busy antenatal care settings in resource-limited areas can be challenging to implement. Hence, targeted approaches to identifying pregnant women at high risk of active TB are required. To strengthen early detection of TB in pregnancy, we conducted a systematic review of findings from studies reporting on the clinical presentation and diagnosis of TB during pregnancy and the postpartum period in LMICs.

## Methods

A protocol for this review was developed and registered with PROSPERO (CRD42020202493). The review was conducted as per the Preferred Reporting Items for Systematic Reviews and Meta-Analyses (PRISMA) guidelines (S1 File).

### Ethical approval

As this was a systematic review of published studies, ethical approval was not required.

### Eligibility criteria

We aimed to include primary research studies published in peer-review journals, including randomized controlled trials, non-randomized or quasi-randomized studies, cohort, case-control, cross-sectional and descriptive studies. Case reports, letters to the editor, commentaries and conference abstracts were excluded. We included those studies involving women in LMICs, defined by the World Bank as countries with a gross national income per capita of less than US$12,375 [14], who were diagnosed with active TB while pregnant or in the postpartum period (up to 6 months after birth). Studies were included regardless of classification by method of TB diagnosis (bacteriologically confirmed or clinically diagnosed) or TB type (pulmonary or extrapulmonary TB). Bacteriologically confirmed TB is defined as a positive biological specimen by smear microscopy, culture, or WHO-approved rapid diagnostics (such as Xpert MTB/RIF) [15]. Clinically diagnosed TB refers to those not bacteriologically confirmed but with a decision to treat for TB disease, based on clinical symptoms and supportive investigations such as chest X-ray findings. Studies of women diagnosed before pregnancy were included, provided there were data relating to women during pregnancy or six months postpartum. Studies involving women who had no disease and evidence of TB infection only, were not eligible. We included all studies regardless of the use of interventions.

### Literature searches, eligibility assessment, data collection, quality assessment and analysis

A search strategy was developed and run in four databases (Ovid MEDLINE, Embase, CINAHL and Global Index Medicus) on 21 July 2020 and updated on 15 January 2023. The search terms were developed around the three key concepts of TB, LMICs, and pregnancy, and the strategy was developed with the assistance of an information specialist (S2 File). There were no limitations on year of publication or language used. Google Translate was used to clarify eligibility of papers in languages other than English. Two reviewers (GS, MP) independently screened and assessed the title and abstracts of the search results and selected potentially eligible studies according to the eligibility criteria. The results of these assessments were compared, and where discrepancies were found this was resolved through discussion or consultation with a third reviewer (AW or JV). The same process of assessing eligibility was conducted based on recovered full texts. Endnote X9 and Covidence online software facilitated this process.

Data from included studies were extracted in duplicate by two reviewers into a pre-designed Excel spreadsheet. Data extracted included characteristics of included studies (author, year, title, design, country, sample size) and demographics of the study population (age, ethnicity, parity, comorbidities, gestational age, use of TB treatment) (S3 File). We classified countries based on TB endemicity according to the WHO Global TB Report 2020 [16]. Review outcomes included women’s clinical presentation with TB and the TB detection methods used, which were extracted from each study: clinical features and duration; onset; diagnostic method(s) employed; and whether pulmonary and/or extrapulmonary TB was present. Discrepancies in data extraction were settled by discussion between reviewers or consulting a third reviewer. Where study reporting was unclear or conflicting, reviewers used the data most often reported, or excluded data that could not be verified.

As the review included a diversity of study designs, we assessed study quality using a six-point checklist adapted from the Newcastle-Ottawa instrument (S4 File) [17–19]. One point was assigned for each checklist item, with the overall study quality score calculated based on the sum of these points. In line with previous reviews, we considered scores of 0 to 2 to be low-quality, scores of 3 or 4 to be moderate quality, and scores of 5 or 6 to be high quality [17–19]. Two reviewers independently assessed the quality of each included study, with discrepancies resolved through discussion or consulting a third reviewer. Studies were not excluded from analysis based on quality assessment.

Extracted data were summarised and reported. Pooled meta-analysis was conducted using a random effects model for all review outcomes (presenting clinical features and detection methods) where more than one study provided data. A random effects model was chosen to maximise accuracy given considerable heterogeneity between studies. Calculated prevalence was impacted by heterogeneity in study design; indicating trends rather than representing population prevalence values. Where multiple studies reported on the same study population, the study with the larger sample size was used. Clinical data were presented as a prevalence in the study population. Detection methods data were presented as a proportion of the study population. Analyses provided the prevalence or proportion, 95% confidence interval, tau squared statistic, and Cochran’s Q statistic and corresponding p value. Cochran’s Q statistic were used to quantify the level of heterogeneity in variance across studies, with larger values suggesting individual prevalence values do not represent a common population prevalence. P values of less than 0.05 were considered statistically significant. Tau squared shows the distribution of heterogeneity between studies [20]. Detection methods data also included the range of the proportions. We conducted an additional sub-analysis of studies with women where pulmonary TB cases only were present, and no TB diagnosis was known prior to detection method testing. The sub-analysis was necessary to determine the influence of these factors on the results. Stata SE 16 software [21] was used for meta-analyses.

## Results

**Fig 1:**
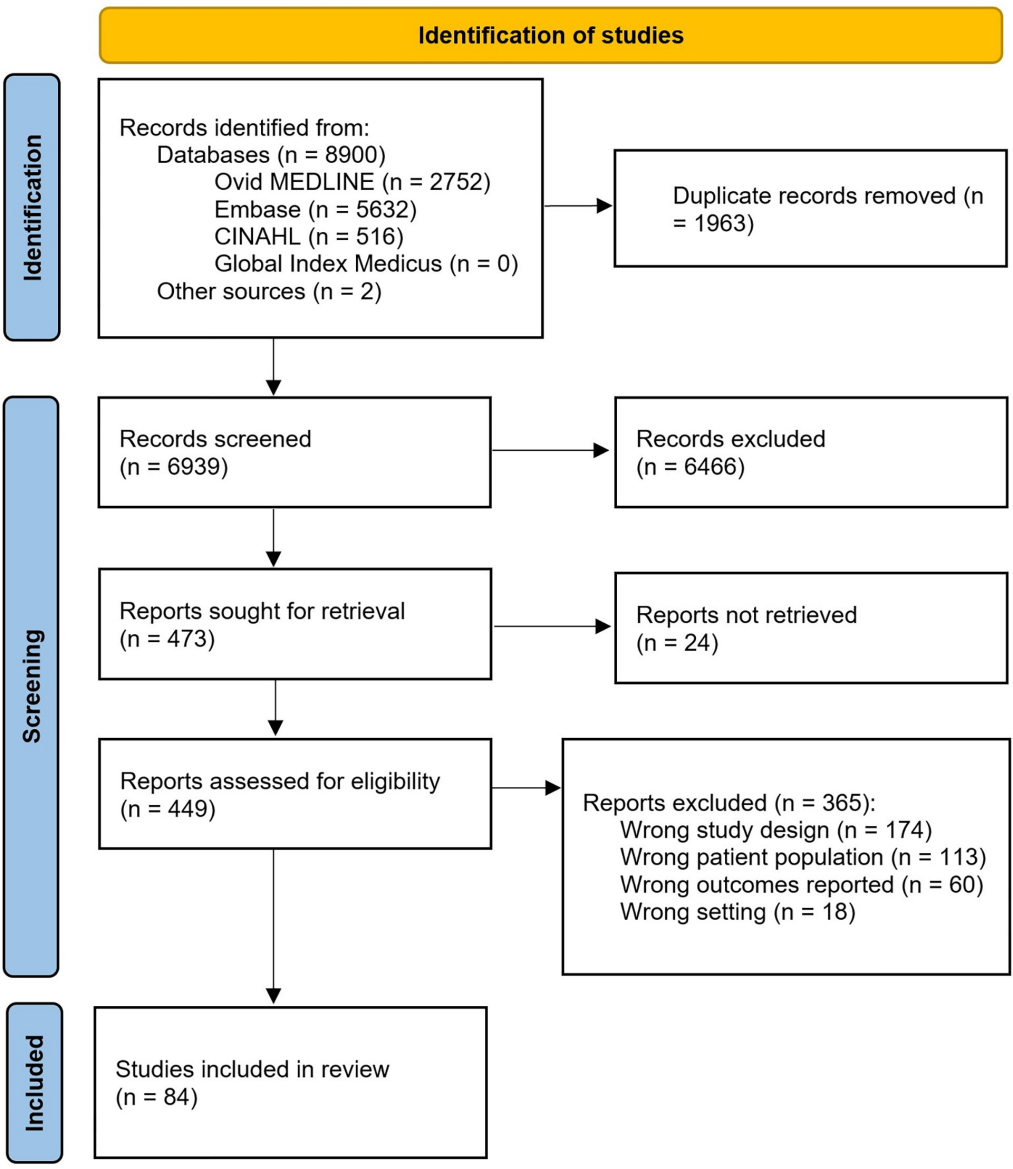
Prisma flow diagram reporting the systematic review. Adapted from: Page MJ, McKenzie JE, Bossuyt PM, Boutron I, Hoffmann TC, Mulrow CD, et al. The PRISMA 2020 statement: an updated guideline for reporting systematic reviews. BMJ 2021;372:n71. doi: 10.1136/bmj.n71.

Literature searches identified 8900 citations, with an additional two studies identified through other sources (further research and reference snowballing) (Fig 1). After removal of duplicates, 6939 studies were screened by title and abstract, and 473 studies were included for full-text review. Full texts could be obtained for 449 studies, of which 365 did not meet the eligibility criteria. In total, 84 studies met inclusion criteria, with two providing multiple datasets, yielding 87 separate studies for analysis.

The 87 studies were published between 1962 to 2022 and most used observational designs (S5 File). Thirty-seven studies were conducted in upper-middle income countries, 39 in lower-middle income countries, 9 in low-income countries, and two studies in multiple countries with various income levels. Across all 87 studies, 23 (26.4%) were conducted in South Africa and 19 (21.8%) in India, together representing 70.4% of women. These countries are both currently WHO-listed high-burden TB settings, while only sixteen studies were conducted in low TB endemic settings (S5 and S6 Files) [16]. On quality assessment, 45 studies (51.7%) were high quality, 33 (37.9%) were moderate quality, and 9 (10.3%) were low quality (S5 File).

The 87 studies included 2,965 women who were pregnant or postpartum and had active TB. In total, 1,459 women (49.2%) had pulmonary TB only, 344 women (11.6%) had extrapulmonary TB only, 30 women (1.0%) had both pulmonary and extrapulmonary TB, and in 1,132 women (38.2%) the type of TB was not specified (Table 1). Amongst the women with extrapulmonary TB, 306 had the site reported ‒ disseminated TB (miliary or TB meningitis) accounted for a third (33.3%) while the most common focal sites were genitourinary (16.7%), osteoarticular (15.7%), and pleural (15.0%) TB (Table 1, S7 File).

**Table 1:** Tuberculosis case characteristics by studies and by women included in those studies.

TB characteristics	Number of studies	%	Number of women	%
Total sample	87	100	2965^a^	100
*Type of TB*				
Pulmonary TB only	38	43.7	1459	49.2
Extrapulmonary TB only	10	11.5	344^b^	11.6
Pulmonary TB and extrapulmonary TB	23^c^	26.4	30^d^	1.0
Not specified	16	18.4	1132	38.2
*TB symptom screen used*				
Yes	28	32.2	400	13.5
No	52	59.8	2496	84.2
Symptom used in study inclusion criteria	7	8.0	69	2.3
*Method of TB diagnosis*				
Clinical (symptoms + positive CXR)	6	6.9	276	9.3
Bacteriological (sputum smear or culture positive)	33	37.9	921	31.1
Both methods utilised	14	16.1	n/a	n/a
Not specified/Incomplete data	34	39.1	1693	57.1

TB: tuberculosis

a. Multiple studies included the same study population. In these cases, the data from the larger study only was included to avoid counting women twice.

b. The types of extrapulmonary TB reported included: miliary/disseminated (n = 62); genitourinary (n = 51); osteoarticular (n = 48); pleural (n = 46); meningitis or central nervous system (n = 40); peripheral lymph node (n = 32); abdominal (n = 12); renal (n = 10); and pericardial (n = 5).

c. Reported both types, either in the same woman or in separate women

d. Both types in the same woman

Table 2 lists reported characteristics of women in the 87 studies, though these were rarely reported. Average mean age was 27 years in the 39 studies that reported this. Comorbid HIV infection was present in 1,325 women (44.7%), though 18 studies enrolled only women living with HIV. Only one case of comorbid diabetes was reported. TB treatment was described for 1,842 women ‒ the majority (89.6%) received treatment for drug susceptible TB (Table 2). Stage of pregnancy or weeks postpartum was specified for 1,687 (56.9%) women. Of those, 1,324 women (78.5%) were antepartum ‒ 42 in the first trimester, 104 in the second trimester and 85 in the third trimester ‒ and 358 women (21.2%) were intrapartum or postpartum. Only 15 studies specifically enrolled women who were in the perinatal or postnatal periods.

**Table 2:** Characteristics of women diagnosed with tuberculosis during pregnancy or within six months postpartum.

Author Year	Study Design	Number of women with TB	Average Age	Stage of pregnancy/ postpartum	Women with comorbid HIV (n/N, %)	TB treatment commenced (DSTB or DRTB treatment)
Adejumo 2020 [22]	Cross sectional	8	-	Antepartum: 8	-	DRTB
Adhikari 1997 [23]	Retrospective cohort	4	19	Antepartum: 1 Postpartum: 3	4/4 (100%)	-
Adjobimey 2022 [24]	Mixed methods cross sectional	2	-	Antepartum: 2	0/2 (0%)	DSTB
Ali 2011 [25]	Case control	42	31	Antepartum: 42	5/42 (12%)	DSTB
Ali 2021 [26]	Retrospective cohort	27	25	Antepartum: 27	-	DSTB: n = 24 DRTB: n = 1 No treatment: 2
Balaka 2004 [27]	Retrospective cohort	13	-	Postpartum: 13	-	DSTB
Baluku, Bongomin 2021 [28]	Retrospective cohort	18	27.5	-	8/18 (44%)	DRTB
Baluku, Nakazibwe 2021 [29]	Retrospective cohort	18	-	-	-	DRTB
Bates 2013 [30]	Prospective cohort	20	-	-	-	-
Bekker 2016 [31]	Prospective cohort	74	29.8	Antepartum: 39 Intrapartum or postpartum: 35	53/74 (72%)	DSTB: n = 68 DRTB: n = 6
Bekker 2012 [32]	Retrospective audit	38	27	Antepartum: 21 Intrapartum or postpartum: 17	25/38 (66%)	DSTB
Berju 2019 [33]	Cross sectional	11	-	Antepartum: 11	4/11 (36%)	-
Bhosale 2021 [34]	Prospective cohort	8	-	Antepartum third trimester:1 Postpartum: 7	4/8 (50%)	DSTB: 7 DRTB: 1
Black 2008 [35]	Retrospective observational	53	-	Antepartum: 53	53/53 (100%)	-
Brar 2021 [36]	Prospective cohort	11	-	Antepartum: 11	-	DSTB
Chansamouth 2016 [37]	Prospective cohort	2	20^a^	Antepartum: 2 (32 weeks: 1)	-	DSTB
Chen 2016 [38]	Retrospective cohort	21	27.2	Antepartum: 21	-	-
Chopra 2017 [39]	Retrospective observational	50	25.74	Antepartum: 50	-	DSTB
Chweneyagae 2012 [40]	Descriptive survey	529	-	-	474/529 (90%)	-
Connor 1970 [41]	Descriptive survey	48	-	Antepartum: 48	-	DSTB
de Oliviera 2011 [42]	Retrospective cohort	7	25.4	Antepartum: 7	2/7 (29%)	DRTB
De Waard 2021 [43]	Prospective cohort	1	29	Postpartum: 1	1/1 (100%)	-
Denti 2016 [44]	Prospective cohort	48	28	Antepartum: 48	48/48 (100%)	DSTB
Desai 2018 [45]	Retrospective cohort	5	25	Antepartum, third trimester: 5	0/5 (0%)	DRTB
Devi 1964 [46]	Prospective cohort	137	-	Antepartum second trimester: 29^b^	-	DSTB
Dong 2022 [47]	Retrospective observational	6	30	Antepartum: 6	-	DSTB
Du 2021 [48]	Retrospective cohort	7	-	Postpartum: 7	-	-
Figueroa-Damian 1998 [49]	Prospective cohort	25	28.2	Antepartum: 25	-	DSTB
Fortes Deguenonvo 2019 [50]	Retrospective descriptive	14	-	-	2/14 (14%)	DSTB
Gai 2021 [51]	Retrospective observational	7	-	Antepartum: 7	-	DSTB
Gounder 2011 [52]	Cross sectional	15	-	Antepartum: 15	10/15 (67%)	DSTB
Gupta 2011 [53]	Randomised trial	26	19: n = 1 25: n = 1	-	26/26 (100%)	-
Gupta 2007 [54]	Prospective cohort	7	-	Postpartum (0–2 weeks): 7	7/7 (100%)	DSTB
Hamda 2020 [55]	Cross sectional	2	-	Antepartum: 2 • First trimester: 1 • Second trimester: 1	1/2 (50%)	DSTB
Heywood 1999 [56]	Descriptive survey	71	-	Antepartum: 70 Postpartum (3 months): 1	-	DSTB
Hoffmann 2013 [57]	Prospective descriptive	49	-	Antepartum: 49	49/49 (100%)	DSTB: n = 43 DRTB: n = 4
Inkaya 2020 [58]	Retrospective audit	1	-	Antepartum: 1	1/1 (100%)	-
Kali 2006 [59]	Cross sectional	8	26	Antepartum: 8	8/8 (100%)	-
Kancheya 2014 [60]	Observational cohort	17	24.9	Antepartum: 17	10/17 (59%)	DSTB
Keskin 2008 [61]	Retrospective observational	2	-	Antepartum, third trimester: 2	-	DSTB
Khan 2000 [62]	Observational study	146	-	Antepartum: 10 Peripartum: 2	115/146 (79%)	DSTB
Khan 2007 [63]	Prospective descriptive study	5	26	-	3/5 (60%)	DRTB
Kosgei 2011 [64]	Cross sectional	3	28.3	Antepartum: 3	3/3 (100%)	-
Kosgei 2013 [65]	Cross-sectional	11	-	Antepartum: 11	10/11 (91%)	DSTB
Kravchenko 2014 [66]	Retrospective cohort	59	26	Antepartum: 59	2/59 (3%)	-
Kriplani 2017 [67]	Randomised controlled trial	21	-	Antepartum, second trimester: 1	-	DSTB
Kumar 1997 [68]	Prospective cohort	10	-	Antepartum: 10	10/10 (100%)	DSTB
Kumar Praveen 2013 [69]	Cross-sectional	212	-	Postpartum: 212	-	-
LaCourse 2016 [70]	Cross sectional	10	-	Antepartum: 10	10/10 (100%)	DSTB
Lawson i 1962 [71]	Prospective observational ANC 1960–61	53	-	Antepartum: 53	-	-
Lawson ii 1962 [71]	Retrospective observational emergency 1960–61	5	-	-	-	-
Lawson iii 1962 [71]	Retrospective observational inpatient 1957–60	69	-	Antepartum: 69	-	DSTB: n = 62 DRTB: n = 7
Letang 2021 [72]	Prospective observational	5	32	Antepartum: 5	5/32 (16%)	-
Loveday, Hlangu 2021 [73]	Prospective qualitative	17	28	-	14/17 (82%)	DRTB
Loveday, Hughes 2021 [74]	Retrospective cohort	108	28	-	88/108 (81%)	DRTB
Mathad 2022 [75]	Case control	7	24	Postpartum: 7	4/7 (57%)	DSTB
Mesic 2020 [76]	Retrospective cohort	8	-	-	-	DRTB
Micozzi 1982 [77]	Retrospective descriptive	4	-	-	-	-
Modi 2016 [78]	Prospective cohort	8	-	Antepartum: 8	-	-
Naranbhai 2014 [79]	Randomised controlled trial	4	-	Postpartum: 4	4/4 (100%)	-
Narayan 2022 [80]	Prospective observational	7	-	-	-	-
Ndwiga 2013 [81]	Operations research/interventional	13	-	Postpartum: 13	3/13 (23%)	-
Odayar 2018 [82]	Retrospective cohort	23	Antenatal (n = 13): 31 Postpartum (n = 10): 29	Antepartum: 13 Postpartum: 10	23/23 (100%)	DSTB
Pasipamire 2020 [83]	Cross sectional	12	-	Antepartum: 12	9/12 (75%)	DSTB
Patil 2012 [84]	Prospecitve descriptive	2	26: n = 1	Antepartum, third trimester: 1	-	DSTB
Pillay a 2001 [85]	Prospective cohort	5	-	-	-	-
Pillay b 2001 [86]	Prospective observational	146	-	-	115/146 (79%)	DSTB: n = 144 DRTB: n = 2
Ranaivomanana 2021 [87]	Prospective cohort	24	25.7	-	-	DSTB
Rendell 2016 [88]	Retrospective observational	104	27	Antepartum: 103 • First trimester: 27 • Second trimester: 37 • Third trimester: 39 Postpartum 1	-	DSTB: n = 102 DRTB: n = 2
Rickman 2020 [89]	Prospective cohort	7	29	Antepartum, third trimester: 7	7/7 (100%)	-
Sabesan 2021 [90]	Prospective observational	1	-	-	-	DSTB
Salazar-Austin 2018 [91]	Prospective cohort	80	29	Pre-pregnancy: 5 Antepartum: 69 • First trimester: 5 • Second trimester: 32 • Third trimester: 32 Postpartum: 3	80/80 (100%)	DSTB
Sengupta 2018 [92]	Prospective observational	8	23: n = 1 31: n = 1 26: n = 1	Antepartum: 7 • First trimester: 4 • Second trimester: 1 • Third trimester: 2 Postpartum: 1	-	DSTB
Shabad 1975 [93]	Observational study	2	-	Antepartum: 2	-	DSTB
Sharma 2021 [94]	Prospective cohort	3	-	Antepartum: 3	-	-
Soibelman 1963 [95]	Retrospective descriptive	59	18: n = 1	Antepartum: 45 Postpartum: 14	-	DSTB
Tiam 2014 [96]	Prospective descriptive	3	-	Antepartum: 3	2/3 (67%)	-
Tripathy 2003 [97]	Case control	111	23.6	Antepartum: 111	-	DSTB: n = 110 DRTB: n = 1
Uwimana i 2013 [98]	Cross sectional survey	2	-	Antepartum: 2	2/2 (100%)	-
Uwimana ii 2013 [98]	Cross sectional survey	4	-	Antepartum: 4	4/4 (100%)	DSTB
van de Water 2020 [99]	Prospective cohort	36	24.5	Antepartum: 36	1/36 (4%)	DSTB: 20 DRTB: 8
van de Walt 2020 [100]	Retrospective observational	26	29	Antepartum: 26	20/26 (77%)	DRTB
Vijayageetha 2019 [101]	Cross sectional	1	-	Antepartum: 1	-	-
Walles 2022 [102]	Prospective cohort	4	24	Antepartum • First trimester: 2 • Second trimester: 2	2/4 (50%)	DSTB
Walles, Tesfaye 2021 [103]	Cross sectional	5	-	-	3/5 (60%)	-
Xia 2022 [104]	Retrospective cohort	59	-	-	0/59 (0%)	DSTB
Yadav 2019 [105]	Retrospective cohort	30	29	Antepartum: 8 • First trimester: 4 • Second trimester: 3 • Third trimester: 1	-	DSTB

HIV: human immunodeficiency virus, DSTB: drug susceptible tuberculosis, DRTB: drug resistant tuberculosis

a. Age data from 1 woman only (n = 1)

b. Stage of pregnancy was reported in only 29 patients

### Clinical features of TB in study populations

Across the 87 studies, 28 (32.2%) utilised a TB symptom screen, 7 (8.0%) included a TB symptom in study inclusion criteria, and 52 (59.8%) did not utilise a TB symptom screen (Table 1). The duration of clinical features was not well reported. Thirty-eight different clinical features were identified, with prevalence data available in at least one study for 27 features only (Table 3, S8 File). Of the 18 features with prevalence data in two or more studies, sputum production had the highest pooled prevalence of 73% (95% CI 57% to 89%), followed by cough with 68% (95% CI 53% to 83%) (S9 File). “Asymptomatic” presentation was prevalent in 58% of those assessed (95% CI 23% to 93%). History of a close TB contact and haemoptysis were rarely identified. Larger and statistically significant Cochran’s Q statistic values found for multiple symptoms suggest high variance in population prevalence between studies and support the use of the random effects model of analysis. A sub-analysis of women diagnosed with pulmonary TB was limited by low sample sizes and found no significant differences (S8 File).

**Table 3:** Prevalence of clinical features reported in pregnant or postpartum (within 6 months) women with tuberculosis.

Clinical feature^a^	Number of reporting studies	Number of studies reporting prevalence	Number of women assessed for clinical feature	Number of women with clinical feature	Pooled Prevalence	Confidence interval	T^2^	Q	Q p value
Sputum production	7	7	116	84	0.73	0.57, 0.89	0.03	52.99	0.00
Cough	25	22	365	238	0.68	0.53, 0.83	0.12	1.50x10^12^	0.00
Vaginal bleeding	2	2	13	8	0.63	0.37, 0.88	0.00	0.65	0.42
Asymptomatic	6	5	77	35	0.58	0.23,0.93	0.14	727.50	0.00
Shortness of breath	7	6	110	57	0.54	0.27, 0.81	0.10	342.12	0.00
Loss of appetite	4	3	46	23	0.50	0.15, 0.85	0.08	16.57	0.00
Fatigue	6	5	69	22	0.48	0.14, 0.82	0.13	497.47	0.00
Fever	21	18	323	121	0.46	0.29, 0.63	0.13	1.33 x10^12^	0.00
History of known TB exposure	7	5	81	39	0.45	0.31, 0.59	0.01	6.54	0.16
Chest pain	3	2	40	14	0.35	0.20, 0.49	0.00	0.42	0.52
Prior TB history	22	20	626	183	0.31	0.19, 0.43	0.07	5.00 x10^11^	0.00
Night sweats	10	10	204	77	0.30	0.14, 0.47	0.06	205.23	0.00
Weight loss/absence of weight gain	17	14	331	107	0.28	0.13, 0.43	0.07	286.08	0.00
Altered sensorium	3	2	13	4	0.28	0.02, 0.53	0.01	1.18	0.28
History of close TB contact	9	9	213	55	0.23	0.12, 0.34	0.02	83.25	0.00
Headache	5	3	20	5	0.23	0.05, 0.41	0.00	0.83	0.66
Haemoptysis	8	6	117	23	0.13	0.00, 0.26	0.02	33.59	0.00
Lymphadenopathy	4	2	24	1	0.01	0.00, 0.08	0.00	1.17	0.28

TB: tuberculosis

a. Clinical features reported only for those reported in >1 study. Clinical features with only 1 study reporting prevalence (number of women with symptom/number of women screened): seizures (2/2), bone and joint pain (40/40), fertility issues (21/21), chills (4/17), swelling (10/50), dizziness (1/7), vomiting (1/7), diplopia (1/7), malaise (2/31).

### Detection methods

The method for diagnosing TB was largely not specified (Table 1). A total of 276 women (9.3%) were diagnosed based on clinical presentation and a positive chest X-ray, and 921 (31.1%) were diagnosed bacteriologically, based on either sputum smear, culture, or Xpert MTB results. There were a multitude of methods for diagnosing extrapulmonary TB, depending on the site (S7 Table in S7 File).

Table 4 presents the yield of each detection method utilised for identifying pregnant or postnatal women with TB (forest plots for each detection method analysis are shown in S10 File). From 11 studies which reported chest X-ray yield, 60% of women (0.60, 95% CI 0.36 to 0.85) with a chest X-ray had findings suggestive of TB. For bacteriological detection methods, the positive test result yield was 23% (95% CI 0% to 50%) for PCR testing, 30% (95% CI 17% to 42%) for sputum smear, and 38% (95% CI 23% to 53%) for sputum culture. However, these yields might be artificially high as some women were known to have TB prior to undergoing a detection method. We therefore conducted a sub-analysis, excluding those studies where women with known TB were screened or tested. This analysis found lower estimated yields for all detection methods examined ‒ 1% (95% CI 1% to 1%) for PCR testing, 12% (95% CI 2% to 23%) for sputum smear, 17% (95% CI 4% to 30%) for sputum culture, and 33% (95% CI 0% to 68%) for chest X-ray (Table 4). Here too, large Cochran’s Q statistic values support using the random effects model for analysis of highly variable population prevalence values between studies.

**Table 4:** TB detection methods used in pregnant and postpartum women, and proportion of women with positive results.

	Studies of women undergoing detection methods								Studies of women undergoing detection methods, excluding those studies where women had a known TB diagnosis							
Question	Number of studies in meta-analysis	Number of women screened/tested	Proportion	Confidence interval	T^2^	Q	Q p value	Ranges of Proportion	Number of studies in meta-analysis	Number of women screened/tested	Proportion	Confidence interval	T2	Q	Q p value	Ranges of Proportion
What proportion of women who underwent symptom screening have TB symptoms?	20	152531	0.21	0.10, 0.31	0.06	1.54x10^7^	0.00	0.00, 1.00	18	152311	0.15	0.08, 0.22	0.02	6425.27	0.00	0.00, 0.61
What proportion of women given a chest X-ray have findings suggestive of TB?	11	3036	0.60	0.36, 0.85	0.17	71090.28	0.00	0.03, 1.00	5	2821	0.33	0.00, 0.68	0.15	101.72	0.00	0.03, 0.85
What proportion of women given a sputum smear have TB?	27	2827	0.30	0.17, 0.42	0.10	8.00x10^11^	0.00	0.00, 1.00	15	2520	0.12	0.02, 0.23	0.04	189.51	0.00	0.00, 0.86
What proportion of women given a sputum culture have TB?	21	3603	0.38	0.23, 0.53	0.12	1.00 x10^12^	0.00	0.00, 1.00	11	3272	0.17	0.04, 0.30	0.05	276.91	0.00	0.00, 0.76
What proportion of women given a PCR test have TB?	8	3820	0.23	0.00, 0.50	0.15	420598.69	0.00	0.00, 1.00	6	3784	0.01	0.01, 0.01	0.00	4.43	0.49	0.00, 0.08
What proportion of women diagnosed with TB are reported to be treated for TB?	46	1756^a^	1.00	1.00, 1.00	0.00	63.98	0.03	0.58, 1.00	-	-	-	-	-	-	-	-

TB: tuberculosis, PCR: polymerase chain reaction

a. 1756 women diagnosed

## Discussion

This systematic review identified 87 studies of 2,965 women that reported on the detection of TB during pregnancy and postpartum. Most studies used observational designs, and most were high or moderate quality. Over 70% of study participants were in South Africa or India (which are TB-endemic countries) and nearly 80% were assessed in the antenatal period. When sought and reported, sputum production (73%) and cough (68%) were the most prevalent clinical features, though 58% of women with TB were asymptomatic. Other common symptoms included shortness of breath (54%), fatigue (48%) and fever (46%), though these features can be hard to distinguish from physiological pregnancy symptoms. Chest X-ray had the highest yield for detection of TB cases (60%), as compared to sputum culture (38%), sputum smear (30%) and PCR testing (23%). This review highlights the challenges for early detection and treatment of TB in maternity care settings.

Most women in this review had pulmonary TB which is consistent with existing international reports in non-pregnant adults [106–108]. As such, pulmonary TB symptoms should be emphasised in screening procedures in maternity settings to maximise identification. For women with extrapulmonary TB, we observed a high proportion with disseminated disease. This is concerning as it can not only lead to worse maternal outcomes and congenital infection, but disseminated disease can be more difficult to identify clinically than pulmonary TB [109–111]. The prevalence data of extrapulmonary TB types are broadly consistent with existing data in non-pregnant adult populations [112–114]. While we found that the majority of TB cases were identified during the antenatal period—predominantly in the second and third trimesters–this might reflect women’s lack of access to early antenatal care services and an associated lack of TB screening in early pregnancy, or perhaps the reactivation of TB later in pregnancy [4, 115, 116]. Testing for HIV status in pregnant women in TB-endemic settings should also be routine, considering that 44.7% of TB cases in this review were also HIV positive.

In non-pregnant adults, the addition of chest X-ray to symptom screening increases TB case detection, including of bacteriologically confirmed TB cases with sub-clinical disease [117]. The use of Xpert MTB/RIF is currently recommended by WHO as the initial diagnostic test in symptomatic adults [118]. A 2022 systematic review identified 22 studies of non-pregnant HIV positive adults and evaluated the diagnostic accuracy of screening techniques for active TB [119]. They reported that the WHO-recommended symptom screen (any one of cough, fever, night sweats, and weight loss) followed by Xpert testing for symptomatic patients had a suboptimal sensitivity of 58%. Universal diagnosis with Xpert alone increased this sensitivity to 68%. For HIV positive patients on anti-retroviral therapy, symptom screen with chest X-ray increased the sensitivity to 89%, however it had low specificity (33%).

TB detection is however more challenging in pregnant women. The immunomodulatory effects of pregnancy, as well as the potential masking of TB symptoms by pregnancy, affect the diagnostic yield of symptom screening alone. For countries with a high TB prevalence (100 cases per 100,000 or greater), WHO recommends considering screening for active TB in pregnant women via standardised symptom screening or chest X-ray as part of routine antenatal care [12]. In those with HIV, more intensified screening in addition to symptom screen such as with molecular rapid diagnostics is suggested [120]. In this review, studies that aimed to detect TB in asymptomatic mothers were limited in terms of sample size and design. There were only six studies reporting asymptomatic antenatal women, most of which focused on systematic screening of pregnant women living with HIV [57, 60, 103, 121]. Most (5/6) found that over 50% of pregnant women with pulmonary TB were asymptomatic. This is supported by other studies from Sweden and India which suggest symptom screening alone is suboptimal in pregnant women [122, 123].

In pregnant and postpartum women, chest X-ray can be used in combination with symptom screening for clinical diagnosis, or it can be used as an initial screening tool, with abnormal radiographs triggering further bacteriologic diagnostic testing. Chest X-rays are considered to be safe in pregnancy ‒ here the clinical benefits of TB diagnosis outweigh the very small radiation risk to the fetus [124]. One barrier to chest X-ray screening is the need for experienced interpreters. While computer-aided detection software appears promising to address this [16], many TB-endemic countries have limited resources for their health systems, and consistent access to radiology services is not guaranteed. The combination of symptom screening with chest X-ray seems the most sensitive option for screening pregnant women, consistent with the findings of Dhana et al’s 2022 systematic review [119]. Based on our findings, we think it likely that other testing modalities are likely to be less useful for initial screening ‒ bacteriological confirmation from sputum using smear, culture or molecular diagnostics each had a yield of 20–40%. However, these results were likely influenced by participant selection, and yield was demonstrably lower when used as a single screening tool in unselected populations. In mothers without a diagnosis of TB, chest X-ray, sputum smear, and culture had a yield of 33%, 12% and 17%, respectively. The optimal use in ante- or post-natal women of molecular WHO-recommended rapid diagnostic tests, such as Xpert MTB/RIF or the more sensitive Xpert Ultra, has not yet been thoroughly evaluated and there are no current guidelines specific to this population, despite Xpert being WHO’s current first line recommendation for presumptive TB cases and WHO endorsement of a range of rapid diagnostics [118].

### Strengths and limitations

Strengths of this review included using a broad search strategy across multiple databases with duplicate screening and data extraction. The inclusion of studies in pregnant and postpartum women from high TB endemic countries is important as this is an under-researched area critical to improving health outcomes in these vulnerable populations in TB endemic countries. Limitations include a relatively limited number of studies and significant heterogeneity between study designs, reducing our ability to draw firmer conclusions from pooled data. Significant heterogeneity is present universally with only underpowered analyses from limited data being more homogenous. Most countries included were lower-middle to upper-middle-income, indicating a lack of research from low-income countries, many of which are TB-endemic. In addition, we found a lack of data related to important variables including TB types and pregnancy or postpartum stage. Despite our best efforts we were unable to locate 24 articles, the absence of which may have affected the conclusions (S11 File). Whilst most studies were assessed as moderate to high quality, possible biases exist where there are inadequate TB case definitions or non-representative sampling of study participants. Also, very few studies focused on postpartum women. We recognise that the prevalence of asymptomatic women (58%) and women with symptoms (for example sputum production prevalence of 73%) appear contradictory–this is because studies have used multiple and differing methods for eliciting TB signs and symptoms. As such, we consider these as representing data trends rather than exact estimates.

### Future directions

WHO has recognised the lack of available data in identifying active TB in pregnant women, and the importance of appropriate management of women with TB to prevent adverse maternal and perinatal outcomes [125]. There is a need for well-designed, prospective cohort studies from TB-endemic settings that include HIV-negative pregnant women, irrespective of symptoms. These studies would improve our understanding of TB disease in pregnancy, and how it affects pregnant and postpartum women. The additional screening and diagnostic yield of tests–particularly chest X-ray and molecular rapid diagnostic testing ‒ in pregnant women who do not have TB-related symptoms is not yet known, nor the optimal test timing. Such evidence is required to improve current clinical guidelines [126]. Molecular WHO-approved rapid diagnostics are not only more sensitive than sputum smear, but also provide an indication of drug susceptibility more rapidly than culture with drug susceptibility testing [118].

## Conclusions

It is critical to identify TB during pregnancy or the postpartum period in order to prevent disease progression and optimise maternal and perinatal outcomes. However, identifying pregnant and postpartum women with TB can be challenging, as they are often asymptomatic or clinical features may be mistaken for physiological changes of pregnancy. Screening and diagnosis with further tests in addition to symptoms—such as chest X-ray and/or rapid diagnostics–are likely to be more effective, but further evaluation in pregnant and postpartum women is needed. These findings indicate that further robust research into the presentation, diagnosis and management of active TB in asymptomatic pregnant and postnatal women, including those without HIV infection, is warranted.

## Supporting information

S1 File PRISMA checklist. (DOC)

S2 File Search strategy. (DOCX)

S3 File List of equivalent terms extracted. (DOCX)

S4 File Six-point checklist adapted from the Newcastle Ottawa checklist. (DOCX)

S5 File Characteristics of included studies. (DOCX)

S6 File Characteristics of the studies by country and income-level. (DOCX)

S7 File Types of extrapulmonary tuberculosis amongst 374 women, and methods by which they were detected. (DOCX)

S8 File Clinical feature prevalence for 38 clinical features of tuberculosis in pregnancy. (DOCX)

S9 File Meta-analyses of clinical features of tuberculosis in pregnancy and postpartum. (DOCX)

S10 File Meta-analysis and forest plots for different methods of detecting TB. (DOCX)

S11 File Full texts unable to be obtained for analysis. (DOCX)

S12 File Protocol. (DOCX)
