# Supplementary material for: The clinical presentation and detection of tuberculosis during pregnancy and in the postpartum period in low- and middle-income countries: A systematic review and meta-analysis
Source: PLOS Glob Public Health. 2023 Aug 23;3(8):e0002222. doi: 10.1371/journal.pgph.0002222 (PMC10446195; doi:10.1371/journal.pgph.0002222)
Supplement: S2 File — (DOCX) [file pgph.0002222.s002.docx]

**Appendix S2: Search Strategy.**

1. Ovid MEDLINE Search Strategy.

| Search Number | Search Terms |
| --- | --- |
| 1 | *tuberculosis/ or *peritonitis, tuberculous/ or exp *tuberculoma/ or exp *tuberculosis, cardiovascular/ or exp *tuberculosis, central nervous system/ or *tuberculosis, cutaneous/ or *tuberculosis, endocrine/ or *tuberculosis, gastrointestinal/ or *tuberculosis, hepatic/ or *tuberculosis, laryngeal/ or exp *tuberculosis, lymph node/ or *tuberculosis, miliary/ or exp *tuberculosis, multidrug-resistant/ or tuberculosis, oral/ or exp *tuberculosis, osteoarticular/ or *tuberculosis, pleural/ or exp *tuberculosis, pulmonary/ or *tuberculosis, splenic/ or *tuberculosis, urogenital/ or *tuberculosis, female genital/ or *tuberculosis, renal/ |
| 2 | tubercul*.mp. [mp=title, abstract, original title, name of substance word, subject heading word, floating sub-heading word, keyword heading word, organism supplementary concept word, protocol supplementary concept word, rare disease supplementary concept word, unique identifier, synonyms] |
| 3 | TB.mp. [mp=title, abstract, original title, name of substance word, subject heading  word, floating sub-heading word, keyword heading word, organism supplementary  concept word, protocol supplementary concept word, rare disease supplementary  concept word, unique identifier, synonyms] |
| 4 | koch? disease.mp. [mp=title, abstract, original title, name of substance word, subject heading word, floating sub-heading word, keyword heading word, organism supplementary concept word, protocol supplementary concept word, rare disease supplementary concept word, unique identifier, synonyms] |
| 5 | 1 or 2 or 3 or 4 |
| 6 | Developing Countries/ |
| 7 | (Afghanistan* or Albania* or Algeria* or Angola* or Argentina* or Armenia* or Azerbaijan* or Bangladesh* or Belarus* or Beliz* or Benin* or Bhutan* or Bolivia* or Bosnia* or Herzegovin* or Botswan* or Brazil* or Bulgaria* or Burkina* or Burundi* or Cabo Verde* or Cape Verde* or Cambodia* or Cameroon* or Central African or Chad* or China or Chinese or Colombia* or Comor* or Congo* or Costa Rica* or Cote d'Ivoir* or Ivory Coast or Cuba* or Djibouti* or Dominica* or Ecuador* or Egypt* or El Salvador* or Eritrea* or Eswatini* or Ethiopia* or Fiji* or Gabon* or Gambia* or Georgia* or Ghana* or Grenad* or Guatemala* or Guinea* or Guyan* or Haiti* or Hondura* or India* or Indonesia* or Iran* or Iraq* or Jamaica* or Jordan* or Kazakhstan* or Kenya* or Kiribati* or Korea* or Kosov* or Kyrgyz Republic or Lao* or Leban* or Lesotho* or Liberia* or Libya* or Macedonia* or Madagascar* or Malawi* or Malaysia* or Maldiv* or Mali* or Marshall Island* or Mauritania* or Mauriti* or Mexic* or Micronesia* or Moldova* or Mongolia* or Montenegr* or Morocc* or Mozambi* or Myanma* or Burmese or Namibia* or Nauru* or Nepal* or Nicaragua* or Niger* or Nigeria* or Pakistan* or Papua New Guinea* or Paraguay* or Peru* or Philippines or Filipino or Romania* or Russia* or Rwanda* or Samoa* or Sao Tome* or Senegal* or Serbia* or Sierra Leon* or Solomon Island* or Somalia* or South Africa* or Sudan* or Sri Lanka* or St Lucia* or St Vincent or Grenadines or Surinam* or Swazi* or Syria* or Tajikistan* or Tanzania* or Thai* or Timor* or Togo* or Tonga* or Tunisia* or Turk* or Turkmenistan* or Tuvalu* or Uganda* or Ukrain* or Uzbekistan* or Vanuatu* or Venezuela* or Vietnam* or West Bank or Gaza or Yemen* or Zambia* or Zimbabwe*).mp. |
| 8 | exp africa/ or exp caribbean region/ or exp central america/ or latin america/ or exp south america/ or asia/ or exp asia, central/ or exp asia, southeastern/ or exp asia, western/ or exp indian ocean islands/ or pacific islands/ or exp melanesia/ or exp micronesia/ or exp west indies/ |
| 9 | (africa* or asia* or caribbean or central america* or latin america* or south america* or melanesia* or micronesia* or polynesia*).mp. |
| 10 | (resource-limit* or resource-poor or low-resource* or limited-resource* or resourceconstrain* or constrain*-resource* or under-resource* or poor*-resource* or resourcescarce* or scarce*-resource* or low-income or middle-income or lowincome or middleincome or (low adj3 middle-income)).mp. |
| 11 | ((developing or underdeveloped or under-developed or emerging or less-developed or least-developed or less-economically developed or least-economically developed or less-affluent or least-affluent or deprived or poor) adj (country or countries or nation or nations or region or regions or economy or economies)).mp. |
| 12 | ((developing or underdeveloped or under-developed or less-developed or leastdeveloped) adj (population* or world)).mp. |
| 13 | (third-world* or thirdworld* or 3rd-world* or lmic or lmics or lami countr* or lalmi countr* or transitional countr*).mp. |
| 14 | (low* adj (gdp or gnp or gross domestic or gross national)).mp. |
| 15 | 6 or 7 or 8 or 9 or 10 or 11 or 12 or 13 or 14 |
| 16 | 5 and 15 |
| 17 | pregnancy/ or gravidity/ or exp labor, obstetric/ or maternal-fetal exchange/ or parity/ or exp parturition/ or placentation/ or pregnancy in adolescence/ or exp pregnancy outcome/ or pregnancy, high-risk/ or exp pregnancy maintenance/ or exp pregnancy, multiple/ or pregnancy, unplanned/ or pregnancy, unwanted/ |
| 18 | pregnan*.mp. [mp=title, abstract, original title, name of substance word, subject heading word, floating sub-heading word, keyword heading word, organism supplementary concept word, protocol supplementary concept word, rare disease supplementary concept word, unique identifier, synonyms] |
| 19 | Prenatal Care/ or Noninvasive Prenatal Testing/ or prenatal.mp. or exp Prenatal Diagnosis/ |
| 20 | pre?natal.mp. [mp=title, abstract, original title, name of substance word, subject heading word, floating sub-heading word, keyword heading word, organism supplementary concept word, protocol supplementary concept word, rare disease supplementary concept word, unique identifier, synonyms] |
| 21 | ante?natal.mp. [mp=title, abstract, original title, name of substance word, subject heading word, floating sub-heading word, keyword heading word, organism supplementary concept word, protocol supplementary concept word, rare disease supplementary concept word, unique identifier, synonyms] |
| 22 | post?natal.mp. or Postnatal Care/ |
| 23 | puerperium.mp. |
| 24 | pre?partum.mp. |
| 25 | ante?partum.mp. |
| 26 | peri?partum.mp. or Peripartum Period/ |
| 27 | post?partum.mp. or exp Postpartum Period/ |
| 28 | matern*.mp. or exp Maternal Health Services/ |
| 29 | gestat*.mp. |
| 30 | Perinatal Death/ or peri*natal.mp. or exp Perinatal Care/ or Perinatal Mortality/ |
| 31 | childbirth.mp. |
| 32 | parturition.mp. |
| 33 | exp Fetal Organ Maturity/ or exp Fetal Development/ or exp Fetal Macrosomia/ or exp Fetal Death/ or exp Maternal-Fetal Exchange/ or f?etal.mp. or exp Fetal Hypoxia/ or exp Fetal Diseases/ or exp Fetal Membranes, Premature Rupture/ or exp Fetal Weight/ or exp Fetal Growth Retardation/ |
| 34 | Fetus/ or F?etus*.mp. |
| 35 | Labor Presentation/ or labo?r.mp. |
| 36 | Obstetrics/ or obstetric?.mp. |
| 37 | placentation.mp. |
| 38 | gravidity.mp. |
| 39 | parity.mp. [mp=title, abstract, original title, name of substance word, subject heading word, floating sub-heading word, keyword heading word, organism supplementary concept word, protocol supplementary concept word, rare disease supplementary concept word, unique identifier, synonyms] |
| 40 | 17 or 18 or 19 or 20 or 21 or 22 or 23 or 24 or 25 or 26 or 27 or 28 or 29 or 30 or 31 or 32 or 33 or 34 or 35 or 36 or 37 or 38 or 39 |
| 41 | 16 and 40 |

2. Embase Search Strategy.

| Search Number | Search Terms |
| --- | --- |
| 1 | *tuberculosis/ or *abdominal tuberculosis/ or *adrenal tuberculosis/ or exp *central  nervous system tuberculosis/ or *congenital tuberculosis/ or exp *drug resistant  tuberculosis/ or *experimental tuberculosis/ or *extrapulmonary tuberculosis/ or exp *gastrointestinal tuberculosis/ or *hepatic tuberculosis/ or *kidney tuberculosis/ or *laryngeal tuberculosis/ or *lung tuberculosis/ or *miliary tuberculosis/ or *ocular tuberculosis/ or *oral tuberculosis/ or *pancreatic tuberculosis/ or *postprimary tuberculosis/ or *primary tuberculosis/ or exp *skin tuberculosis/ or *splenic tuberculosis/ or *thyroid tuberculosis/ or *tuberculoma/ or *tuberculous arthritis/ or *tuberculous empyema/ or *tuberculous lymphadenitis/ or *tuberculous osteomyelitis/ or *tuberculous pericarditis/ or *tuberculous peritonitis/ or *tuberculous pleurisy/ or *tuberculous spondylitis/ |
| 2 | urogenital tuberculosis/ or *female genital tuberculosis/ |
| 3 | *Mycobacterium tuberculosis/ or *tuberculosis rapid test/ or *multidrug resistant  tuberculosis/ or *tuberculosis control/ or *Mycobacterium tuberculosis test kit/ |
| 4 | tubercul*.mp. [mp=title, abstract, heading word, drug trade name, original title, device manufacturer, drug manufacturer, device trade name, keyword, floating subheading word, candidate term word] |
| 5 | TB.mp. [mp=title, abstract, heading word, drug trade name, original title, device  manufacturer, drug manufacturer, device trade name, keyword, floating subheading word, candidate term word] |
| 6 | koch? disease.mp. [mp=title, abstract, heading word, drug trade name, original title, device manufacturer, drug manufacturer, device trade name, keyword, floating subheading word, candidate term word] |
| 7 | 1 or 2 or 3 or 4 or 5 or 6 |
| 8 | developing country/ |
| 9 | (Afghanistan* or Albania* or Algeria* or Angola* or Argentina* or Armenia* or  Azerbaijan* or Bangladesh* or Belarus* or Beliz* or Benin* or Bhutan* or Bolivia* or Bosnia* or Herzegovin* or Botswan* or Brazil* or Bulgaria* or Burkina* or Burundi* or Cabo Verde* or Cape Verde* or Cambodia* or Cameroon* or Central African or Chad* or China or Chinese or Colombia* or Comor* or Congo* or Costa Rica* or Cote  d'Ivoir* or Ivory Coast or Cuba* or Djibouti* or Dominica* or Ecuador* or Egypt* or El Salvador* or Eritrea* or Eswatini* or Ethiopia* or Fiji* or Gabon* or Gambia* or  Georgia* or Ghana* or Grenad* or Guatemala* or Guinea* or Guyan* or Haiti* or  Hondura* or India* or Indonesia* or Iran* or Iraq* or Jamaica* or Jordan* or  Kazakhstan* or Kenya* or Kiribati* or Korea* or Kosov* or Kyrgyz Republic or Lao* or Leban* or Lesotho* or Liberia* or Libya* or Macedonia* or Madagascar* or Malawi* or Malaysia* or Maldiv* or Mali* or Marshall Island* or Mauritania* or Mauriti* or Mexic* or Micronesia* or Moldova* or Mongolia* or Montenegr* or Morocc* or Mozambi* or Myanma* or Burmese or Namibia* or Nauru* or Nepal* or Nicaragua* or Niger* or Nigeria* or Pakistan* or Papua New Guinea* or Paraguay* or Peru* or Philippines or Filipino or Romania* or Russia* or Rwanda* or Samoa* or Sao Tome* or Senegal* or Serbia* or Sierra Leon* or Solomon Island* or Somalia* or South Africa* or Sudan* or Sri Lanka* or St Lucia* or St Vincent or Grenadines or Surinam* or Swazi* or Syria* or Tajikistan* or Tanzania* or Thai* or Timor* or Togo* or Tonga* or Tunisia* or Turk* or Turkmenistan* or Tuvalu* or Uganda* or Ukrain* or Uzbekistan* or Vanuatu* or Venezuela* or Vietnam* or West Bank or Gaza or Yemen* or Zambia* or Zimbabwe*).mp. |
| 10 | exp Africa/ or exp caribbean/ or exp caribbean islands/ or exp "South and Central  America"/ or exp Asia/ or exp indian ocean/ or exp pacific ocean/ |
| 11 | (africa* or asia* or caribbean or central america* or latin america* or south america* or melanesia* or micronesia* or polynesia*).mp. |
| 12 | (resource-limit* or resource-poor or low-resource* or limited-resource* or resourceconstrain* or constrain*-resource* or under-resource* or poor*-resource* or resourcescarce* or scarce*-resource* or low-income or middle-income or lowincome or middleincome or (low adj3 middle-income)).mp. |
| 13 | ((developing or underdeveloped or under-developed or emerging or less-developed or least-developed or less-economically developed or least-economically developed or less-affluent or least-affluent or deprived or poor) adj (country or countries or nation or nations or region or regions or economy or economies)).mp. |
| 14 | ((developing or underdeveloped or under-developed or less-developed or leastdeveloped) adj (population* or world)).mp. |
| 15 | (third-world* or thirdworld* or 3rd-world* or lmic or lmics or lami countr* or lalmi  countr* or transitional countr*).mp. |
| 16 | (low* adj (gdp or gnp or gross domestic or gross national)).mp. |
| 17 | 8 or 9 or 10 or 11 or 12 or 13 or 14 or 15 or 16 |
| 18 | 7 and 17 |
| 19 | exp triplet pregnancy/ or exp second trimester pregnancy/ or exp third trimester  pregnancy/ or exp pregnancy disorder/ or exp first trimester pregnancy/ or exp pregnancy termination/ or exp angular pregnancy/ or exp adolescent pregnancy/ or exp prolonged pregnancy/ or exp "parameters concerning the fetus, newborn and pregnancy"/ or exp pregnancy outcome/ or exp ectopic pregnancy/ or exp uterine tube pregnancy/ or exp twin pregnancy/ or exp cornual pregnancy/ or exp interstitial pregnancy/ or exp ovary pregnancy/ or exp pregnancy/ or exp pregnancy diabetes mellitus/ or exp high risk pregnancy/ or exp unwanted pregnancy/ or exp unplanned pregnancy/ or exp multiple pregnancy/ or exp pregnancy complication/ or exp quintuplet pregnancy/ or exp quadruplet pregnancy/ |
| 20 | gravidity.mp. |
| 21 | exp labor complication/ or exp labor stage 1/ or exp labor/ or exp labor stage 3/ or exp "immature and premature labor"/ or exp labor stage 2/ or exp premature labor/ |
| 22 | labo?r.mp. |
| 23 | exp obstetric delivery/ or exp obstetric patient/ or obstetric*.mp. or exp obstetric  hemorrhage/ or exp "gynecological and obstetric diagnostic device"/ or exp obstetric procedure/ or exp obstetric operation/ or exp obstetric emergency/ |
| 24 | (f?etal or f?etus).mp. [mp=title, abstract, heading word, drug trade name, original title, device manufacturer, drug manufacturer, device trade name, keyword, floating subheading word, candidate term word] |
| 25 | parity.mp. or exp parity/ |
| 26 | parturition.mp. or birth/ |
| 27 | placentation.mp. or placenta development/ |
| 28 | pregnan*.mp. |
| 29 | exp prenatal mortality/ or exp prenatal development/ or exp prenatal growth/ or exp prenatal care/ or exp prenatal period/ or pre?natal.mp. or exp prenatal stress/ or exp prenatal diagnosis/ or exp noninvasive prenatal testing/ or exp non-invasive prenatal test kit/ or exp prenatal screening/ or exp prenatal disorder/ |
| 30 | ante?natal.mp. or exp antenatal depression/ |
| 31 | exp postnatal care/ or exp postnatal growth/ or exp postnatal development/ or exp  postnatal depression/ or post?natal.mp. |
| 32 | puerperium.mp. or exp puerperium/ |
| 33 | pre?partum.mp. or exp lactation/ |
| 34 | exp antepartum hemorrhage/ or ante?partum.mp. |
| 35 | exp peripartum cardiomyopathy/ or peri?partum.mp. |
| 36 | exp postpartum pain/ or post?partum.mp. or exp postpartum hemorrhage/ or exp  postpartum thyroiditis/ |
| 37 | exp birth weight/ or gestat*.mp. or exp fetus/ |
| 38 | exp fetal well being/ or exp fetal therapy/ or exp fetal hemorrhage/ or exp fetal tumor/ or exp fetal malnutrition/ |
| 39 | exp mother fetus relationship/ or exp fetus outcome/ or exp fetus distress/ or exp fetus death/ or exp fetus development/ or exp fetus wastage/ or exp fetus risk/ or exp fetus growth/ or exp fetus weight/ or exp fetus disease/ |
| 40 | exp perinatal infection/ or exp perinatal stress/ or exp perinatal depression/ or exp  perinatal morbidity/ or peri*natal.mp. or exp perinatal death/ or exp perinatal mortality/ or exp perinatal development/ or exp perinatal care/ or exp perinatal asphyxia/ or exp perinatal period/ |
| 41 | exp childbirth/ or childbirth.mp. |
| 42 | exp maternal age/ or matern*.mp. |
| 43 | 19 or 20 or 21 or 22 or 23 or 24 or 25 or 26 or 27 or 28 or 29 or 30 or 31 or 32 or 33 or 34 or 35 or 36 or 37 or 38 or 39 or 40 or 41 or 42 |
| 44 | 18 and 43 |

3. CINAHL Search Strategy.

| Search Number | Search Terms |
| --- | --- |
| 1 | (MM "Mycobacterium Tuberculosis") OR (MM "Tuberculosis") OR (MM  "Tuberculosis, Central Nervous System") OR (MM "Tuberculosis,  Gastrointestinal") OR (MM "Tuberculosis, Meningeal") OR (MM "Tuberculosis, Multidrug-Resistant") OR (MM "Tuberculosis, Ocular") OR  (MM "Tuberculosis, Osteoarticular") OR (MM "Tuberculosis, Pulmonary")  OR (MM "Tuberculosis, Spinal") ) OR tubercul* OR TB OR koch# disease |
| 2 | ( (MH "Developing Countries") OR (MH "Low and Middle Income Countries") ) OR ( Afghanistan* or Albania* or Algeria* or Angola* or Argentina* or Armenia* or Azerbaijan* or Bangladesh* or Belarus* or Beliz* or Benin* or Bhutan* or Bolivia* or Bosnia* or Herzegovin* or Botswan* or Brazil* or Bulgaria* or Burkina* or Burundi* or Cabo Verde* or Cape Verde* or Cambodia* or Cameroon* or Central African or Chad* or China or Chinese or Colombia* or Comor* or Congo* or Costa Rica* or Cote d'Ivoir* or Ivory Coast or Cuba* or Djibouti* or Dominica* or Ecuador* or Egypt* or El Salvador* or Eritrea* or Eswatini* or Ethiopia* or Fiji* or Gabon* or Gambia* or Georgia* or Ghana* or Grenad* or Guatemala* or Guinea* or Guyan* or Haiti* or Hondura* or India* or Indonesia* or Iran* or Iraq* or Jamaica* or Jordan* or Kazakhstan* or Kenya* or Kiribati* or Korea* or Kosov* or Kyrgyz Republic or Lao* or Leban* or Lesotho* or Liberia* or Libya* or Macedonia* or Madagascar* or Malawi* or Malaysia* or Maldiv* or Mali* or Marshall Island* or Mauritania* or Mauriti* or Mexic* or Micronesia* or Moldova* or Mongolia* or Montenegr* or Morocc* or Mozambi* or Myanma* or Burmese or Namibia* or Nauru* or Nepal* or Nicaragua* or Niger* or Nigeria* or Pakistan* or Papua New Guinea* or Paraguay* or Peru* or Philippines or Filipino or Romania* or Russia* or Rwanda* or Samoa* or Sao Tome* or Senegal* or Serbia* or Sierra Leon* or Solomon Island* or Somalia* or South Africa* or Sudan* or Sri Lanka* or St Lucia* or St Vincent or Grenadines or Surinam* or Swazi* or Syria* or Tajikistan* or Tanzania* or Thai* or Timor* or Togo* or Tonga* or Tunisia* or Turk* or Turkmenistan* or Tuvalu* or Uganda* or Ukrain* or Uzbekistan* or Vanuatu* or Venezuela* or Vietnam* or West Bank or Gaza or Yemen* or Zambia* or Zimbabwe* ) OR ( (MH "Africa+") OR (MH "West Indies+") OR (MH "Central America+") OR (MH "Latin America") OR (MH "South America+") OR (MH "Asia") OR (MH "Asia, Central+") OR (MH "Asia, Southeastern+") OR (MH "Asia, Western+") OR (MH "Indian Ocean Islands+") OR (MH "Pacific Islands") OR (MH "Melanesia") OR (MH "Micronesia+") ) OR ( africa* or asia* or caribbean or central america* or latin america* or south america* or melanesia* or micronesia* or polynesia* ) OR ( resource-limit* or resource-poor or low-resource* or limited-resource* or resource-constrain* or constrain*-resource* or under-resource* or poor*-resource* or resource-scarce* or scarce*-resource* or low-income or middle-income or lowincome or middleincome or (low N3 middle-income) ) OR ( (developing or underdeveloped or under-developed or emerging or less-developed or least-developed or less-economically developed or least-economically developed or less-affluent or least-affluent or deprived or poor) N2 (country or countries or nation or nations or region or regions or economy or economies) ) OR ( (developing or underdeveloped or under-developed or less-developed or least-developed) N2 (population* or world) ) OR ( third-world* or thirdworld* or 3rd-world* or lmic or lmics or lami countr* or lalmi countr* or transitional countr* ) OR ( low* N2 (gdp or gnp or gross domestic or gross national) ) |
| 3 | ( (MH "Pregnancy+") OR (MH "Postnatal Period+") OR (MH "Parity") OR (MH "Fetal Development+") OR (MH "Pregnancy, Multiple+") OR (MH "Pregnancy Trimesters+") OR (MH "Pregnancy Outcomes") OR (MH "Pregnancy Complications+") OR (MH "Pregnancy Termination Care (Iowa NIC)") OR (MH "Pregnancy in Adolescence") OR (MH "Pregnancy Tests, Immunologic") OR (MH "Obstetric Emergencies") OR (MH "Obstetric Care+") OR (MH "Obstetric Patients") OR (MH "Obstetric Nursing") OR (MH "Perinatal Nursing") OR (MH "Obstetric Service") OR (MH "Maternal-Child Health") OR (MH "Maternal Age+") OR (MH "Fetal Abnormalities") OR (MH "Fetal Diseases+") OR (MH "Fetus+") OR (MH "Obstetrics") OR (MH "Birth Weight") OR (MH "Birth Injuries+") OR (MH "Placental Hormones+") OR (MH "Pregnancy Proteins+") OR (MH "Prenatal Care (Iowa NIC)") OR (MH "Gestational Weight Gain") OR (MH "Perinatal Death") ) OR ( pregnan* or obstetric* or matern* or labo#r or gravidity f#etal OR f#etus or parity or parturition or placentation or pre#natal or ante#natal or post#natal or puerperium or pre#partum or ante#partum or peri#partum of post#partum or gestat* or peri#natal or childbirth ) |
| 4 | 1 AND 2 AND 3 |

4. Global Index Medicus Search Strategy.

| Search Number | Search Terms |
| --- | --- |
| 1 | (tw:(tubercul* or TB or koch? disease)) AND (tw:(pregnan* or obstetric* or matern* or labo?r or gravidity f?etal OR f?etus or parity or parturition or placentation or pre?natal or ante?natal or post?natal or puerperium or pre?partum or ante?partum or peri?partum of post?partum or gestat* or peri?natal or childbirth)) AND (tw:(Afghanistan* or Albania* or Algeria* or Angola* or Argentina* or Armenia* or Azerbaijan* or Bangladesh* or Belarus* or Beliz* or Benin* or Bhutan* or Bolivia* or Bosnia* or Herzegovin* or Botswan* or Brazil* or Bulgaria* or Burkina* or Burundi* or Cabo Verde* or Cape Verde* or Cambodia* or Cameroon* or Central African or Chad* or China or Chinese or Colombia* or Comor* or Congo* or Costa Rica* or Cote d'Ivoir* or Ivory Coast or Cuba* or Djibouti* or Dominica* or Ecuador* or Egypt* or El Salvador* or Eritrea* or Eswatini* or Ethiopia* or Fiji* or Gabon* or Gambia* or Georgia* or Ghana* or Grenad* or Guatemala* or Guinea* or Guyan* or Haiti* or Hondura* or India* or Indonesia* or Iran* or Iraq* or Jamaica* or Jordan* or Kazakhstan* or Kenya* or Kiribati* or Korea* or Kosov* or Kyrgyz Republic or Lao* or Leban* or Lesotho* or Liberia* or Libya* or Macedonia* or Madagascar* or Malawi* or Malaysia* or Maldiv* or Mali* or Marshall Island* or Mauritania* or Mauriti* or Mexic* or Micronesia* or Moldova* or Mongolia* or Montenegr* or Morocc* or Mozambi* or Myanma* or Burmese or Namibia* or Nauru* or Nepal* or Nicaragua* or Niger* or Nigeria* or Pakistan* or Papua New Guinea* or Paraguay* or Peru* or Philippines or Filipino or Romania* or Russia* or Rwanda* or Samoa* or Sao Tome* or Senegal* or Serbia* or Sierra Leon* or Solomon Island* or Somalia* or South Africa* or Sudan* or Sri Lanka* or St Lucia* or St Vincent or Grenadines or Surinam* or Swazi* or Syria* or Tajikistan* or Tanzania* or Thai* or Timor* or Togo* or Tonga* or Tunisia* or Turk* or Turkmenistan* or Tuvalu* or Uganda* or Ukrain* or Uzbekistan* or Vanuatu* or Venezuela* or Vietnam* or West Bank or Gaza or Yemen* or Zambia* or Zimbabwe* or africa* or asia* or caribbean or central america* or latin america* or south america* or melanesia* or micronesia* or polynesia* or resource-limit* or resource-poor or low-resource* or limited-resource* or resource-constrain* or constrain*-resource* or under-resource* or poor*-resource* or resource-scarce* or scarce*-resource* or low-income or middle-income or lowincome or middleincome or (low adj3 middle-income) or (developing or underdeveloped or under-developed or emerging or less-developed or least-developed or less-economically developed or least-economically developed or less-affluent or least-affluent or deprived or poor) adj (country or countries or nation or nations or region or regions or economy or economies) or (developing or underdeveloped or under-developed or less-developed or least-developed) adj (population* or world) or third-world* or thirdworld* or 3rd-world* or lmic or lmics or lami countr* or lalmi countr* or transitional countr* or low* adj (gdp or gnp or gross domestic or gross national) or caribbean or indian ocean islands or pacific islands or west indies)) |
