# Supplementary material for: The clinical presentation and detection of tuberculosis during pregnancy and in the postpartum period in low- and middle-income countries: A systematic review and meta-analysis
Source: PLOS Glob Public Health. 2023 Aug 23;3(8):e0002222. doi: 10.1371/journal.pgph.0002222 (PMC10446195; doi:10.1371/journal.pgph.0002222)
Supplement: S3 File — (DOCX) [file pgph.0002222.s003.docx]

**Appendix S3: List of equivalent terms extracted.**

| **Reported Term** | **Equivalent terms from article** |
| --- | --- |
| fatigue | - tiredness |
| sputum production | - expectoriation - productive cough |
| convulsions | - seizures |
| ankle swelling | - pedal edema |
| Shortness of breath | - difficulty breathing |
