# Supplementary material for: The clinical presentation and detection of tuberculosis during pregnancy and in the postpartum period in low- and middle-income countries: A systematic review and meta-analysis
Source: PLOS Glob Public Health. 2023 Aug 23;3(8):e0002222. doi: 10.1371/journal.pgph.0002222 (PMC10446195; doi:10.1371/journal.pgph.0002222)
Supplement: S4 File — (DOCX) [file pgph.0002222.s004.docx]

**Appendix S4: Six-point checklist adapted from the Newcastle Ottawa checklist**

Adequate description of study population (ie. Multiple key parameters described, inclusion/exclusion criteria)
Y = study population well described
N = study population is not well described

Random selection used
Y = a census or some form of random sampling (eg. simple random sampling, stratified random sampling, cluster sampling, systematic sampling)
N = not a census and random sampling not used

Acceptable case definition
Y = acceptable case definition used (description of how TB cases were defined)
N = no acceptable case definition used

Consistent mode of data collection
Y = same mode of data collection used for all subjects
N = same mode of data collection not used for all subjects

Consistency of reported data
Y = data is consistent throughout paper
N = data is not consistent throughout paper

Minimisation of likelihood of non-response bias
Y = data from direct observation, or in surveys rate of response >75%, or comparability between respondent and non-respondent characteristics was established
N = rate of response <75%, or comparability between respondent and non-respondent was unsatisfactory or not described

**Total points**Allocate 1 point for every “Y”:
Low quality = 0-2
Moderate quality = 3-4
High quality = 5-6
