# Supplementary material for: The clinical presentation and detection of tuberculosis during pregnancy and in the postpartum period in low- and middle-income countries: A systematic review and meta-analysis
Source: PLOS Glob Public Health. 2023 Aug 23;3(8):e0002222. doi: 10.1371/journal.pgph.0002222 (PMC10446195; doi:10.1371/journal.pgph.0002222)
Supplement: S5 File — (DOCX) [file pgph.0002222.s005.docx]

**Appendix S5: Characteristics of included studies**

Table S1. Characteristics of included studies

| **Author Year** | **Study Design** | **Country** | **Country income level^1^** | **High TB endemic setting^2^** | **Pregnant/6 months postpartum women with active TB** | **Study Quality** |
| --- | --- | --- | --- | --- | --- | --- |
| Adejumo 2020 | Cross-sectional | Nigeria | Lower middle income | Y | 8 | High |
| Adhikari 1997 | Retrospective Cohort | South Africa | Upper middle income | Y | 4 | High |
| Adjobimey 2022 | Mixed-methods cross-sectional | Benin | Lower middle income | N | 2 | High |
| Ali 2011 | Case control | Sudan | Low income | N | 42 | Moderate |
| Ali 2021 | Retrospective cohort | Pakistan | Lower middle income | Y | 27 | High |
| Balaka 2004 | Retrospective cohort | Togo | Low income | N | 13 | Moderate |
| Baluku, Bongomin 2021 | Retrospective cohort | Uganda | Low income | N | 18 | High |
| Baluyku, Nakazibwe 2021 | Retrospective cohort | Uganda | Low income | N | 18 | High |
| Bates 2013 | Prospective Cohort | Zambia | Lower middle income | Y | 20 | High |
| Bekker 2016 | Prospective Cohort | South Africa | Upper middle income | Y | 74 | High |
| Bekker 2012 | Retrospective audit | South Africa | Upper middle income | Y | 38 | High |
| Berju 2019 | Cross sectional | Ethiopia | Low income | Y | 11 | High |
| Bhosale 2021 | Prospective cohort | India | Lower middle income | Y | 8 | High |
| Black 2008 | Retrospective Observational | South Africa | Upper middle income | Y | 53 | High |
| Brar 2021 | Prospective cohort | India | Lower middle income | Y | 11 | Moderate |
| Chansamouth 2016 | Prospective Cohort | Laos | Lower middle income | N | 2 | High |
| Chen 2016 | Retrospective Cohort | China | Upper middle income | Y | 21 | Moderate |
| Chopra 2017 | Retrospective observational | India | Lower middle income | Y | 50 | Low |
| Chweneyagae 2012 | Descriptive Survey | South Africa | Upper middle income | Y | 529 | Moderate |
| Connor 1970 | Descriptive Survey | South Africa | Upper middle income | Y | 48 | Moderate |
| de Oliviera 2011 | Retrospective Cohort | Brazil | Upper middle income | Y | 7 | Low |
| de Waard 2021 | Prospective cohort | South Africa | Upper middle income | Y | 1 | High |
| Denti 2016 | Prospective Cohort | South Africa | Upper middle income | Y | 48 | Moderate |
| Desai 2018 | Retrospective Cohort | India | Lower middle income | Y | 5 | High |
| Devi 1964 | Prospective Cohort | India | Lower middle income | Y | 137 | Moderate |
| Dong 2022 | Retrospective observational | China | Upper middle income | Y | 6 | High |
| Du 2021 | Retrospective cohort | China | Upper middle income | Y | 7 | High |
| Figueroa-Damian 1998 | Prospective Cohort | Mexico | Upper middle income | N | 25 | Moderate |
| Fortes Deguenonvo 2019 | Retrospective descriptive | Senegal | Lower middle income | N | 14 | High |
| Gai 2021 | Retrospective observational | China | Upper middle income | Y | 7 | High |
| Gounder 2011 | Cross sectional | South Africa | Upper middle income | Y | 15 | Moderate |
| Gupta 2011 | Randomised Trial | India | Lower middle income | Y | 26 | Moderate |
| Gupta 2007 | Prospective Cohort | India | Lower middle income | Y | 7 | Moderate |
| Hamda 2020 | Cross sectional | Botswana | Upper middle income | N | 2 | High |
| Heywood 1999 | Descriptive Survey | Papua New Guinea | Lower middle income | Y | 71 | Moderate |
| Hoffmann 2013 | Prospective descriptive | South Africa | Upper middle income | Y | 49 | High |
| Inkaya 2020 | Retrospective audit | Turkey | Upper middle income | N | 1 | Moderate |
| Kali 2006 | Cross sectional | South Africa | Upper middle income | Y | 8 | High |
| Kancheya 2014 | Observational Cohort | Zambia | Lower middle income | Y | 17 | High |
| Keskin 2008 | Retrospective observational | Turkey | Upper middle income | N | 2 | Moderate |
| Khan 2000 | Observational study | South Africa | Upper middle income | Y | 146 | Moderate |
| Khan 2007 | Prospective descriptive study | South Africa | Upper middle income | Y | 5 | Moderate |
| Kosgei 2011 | Cross sectional | Kenya | Lower middle income | Y | 3 | High |
| Kosgei 2013 | Cross-sectional | Kenya | Lower middle income | Y | 11 | Moderate |
| Kravchenko 2014 | Retrospective Cohort | Russia | Upper middle income | Y | 59 | Low |
| Kriplani 2017 | Randomised Controlled Trial | India | Lower middle income | Y | 21 | High |
| Kumar 1997 | Prospective Cohort | India | Lower middle income | Y | 10 | Moderate |
| Kumar Praveen 2013 | Cross-sectional | India | Lower middle income | Y | 212 | Low |
| LaCourse 2016 | Cross Sectional | Kenya | Lower middle income | Y | 10 | High |
| Lawson i 1962 | Prospective Observational ANC 1960-61 | Nigeria | Lower middle income | Y | 53 | Low |
| Lawson ii 1962 | Retrospective Observational Emergency 1960-61 | Nigeria | Lower middle income | Y | 5 |  |
| Lawson iii 1962 | Retrospective Observational Inpatient 1957-60 | Nigeria | Lower middle income | Y | 69 |  |
| Letang 2021 | Prospective observational | Mozambique/Brazil | Low income/Upper middle income | Y/Y | 5 | High |
| Loveday, Hlangu 2021 | Prospective qualitative study | South Africa | Upper middle income | Y | 17 | High |
| Loveday, Hughes 2021 | Retrospective cohort | South Africa | Upper middle income | Y | 108 | High |
| Mathad 2022 | Prospective case-control | India | Lower middle income | Y | 7 | High |
| Mesic 2020 | Retrospective cohort | Afghanistan | Low income | N | 8 | High |
| Micozzi 1982 | Retrospective descriptive | Philippines | Lower middle income | Y | 4 | Moderate |
| Modi 2016 | Prospective Cohort | Kenya | Lower middle income | Y | 8 | High |
| Naranbhai 2014 | Randomised Controlled Trial | South Africa, Tanzania, Uganda, and Zimbabwe | South Africa: Upper middle income Tanzania: Lower middle income Uganda: Low income Zimbabwe: Lower middle income | South Africa: Y Tanzania: Y  Uganda: N Zimbabwe: Y | 4 | High |
| Narayan 2022 | Prospective observational | India | Lower middle income | Y | 7 | Moderate |
| Ndwiga 2013 | Operations research/interventional | Kenya | Lower middle income | Y | 13 | Moderate |
| Odayar 2018 | Retrospective cohort | South Africa | Upper middle income | Y | 23 | High |
| Pasipamire 2020 | Cross-sectional | Eswatini | Lower middle income | N | 12 | High |
| Patil 2012 | Prospecitve descriptive | India | Lower middle income | Y | 2 | Moderate |
| Pillay a 2001 | Prospective Cohort | South Africa | Upper middle income | Y | 5 | High |
| Pillay b 2001 | Prospective observational | South Africa | Upper middle income | Y | 146 | High |
| Ranaivomanana 2021 | Prospective cohort | Madagascar | Low income | N | 24 | Moderate |
| Rendell 2016 | Retrospective observational | Mongolia | Lower middle income | N | 104 | Moderate |
| Rickman 2020 | Prospective cohort | South Africa | Upper middle income | Y | 7 | Moderate |
| Sabesan 2021 | Prospective observational | India | Lower middle income | Y | 1 | High |
| Salazar-Austin 2018 | Prospective cohort | South Africa | Upper middle income | Y | 80 | High |
| Sengupta 2018 | Prospective observational | India | Lower middle income | Y | 8 | Moderate |
| Shabad 1975 | Observational Study | Russia | Upper middle income | Y | 2 | Moderate |
| Sharma 2021 | Prospective cohort | India | Lower middle income | Y | 3 | High |
| Soibelman 1963 | Retrospective descriptive | Russia | Upper middle income | Y | 59 | Low |
| Tiam 2014 | Prospective descriptive | Lesotho | Lower middle income | Y | 3 | Moderate |
| Tripathy 2003 | Case control | India | Lower middle income | Y | 111 | Low |
| Uwimana i 2013 | Cross sectional survey | South Africa | Upper middle income | Y | 2 | Moderate |
| Uwimana ii 2013 | Cross sectional survey | South Africa | Upper middle income | Y | 4 |  |
| van de Water 2020 | Prospective cohort | Peru | Upper middle income | N | 36 | High |
| van der Walt 2020 | Retrospective observational | South Africa | Upper middle income | Y | 26 | High |
| Vijayageetha 2019 | Cross sectional | India | Lower middle income | Y | 1 | High |
| Walles 2022 | Prospective cohort | Ethiopia | Low income | Y | 4 | High |
| Walles 2021 | Cross-sectional | Ethiopia | Low income | Y | 5 | Moderate |
| Xia 2022 | Retrospective cohort | China | Upper middle income | Y | 59 | High |
| Yadav 2019 | Retrospective cohort | India | Lower middle income | Y | 30 | Moderate |

TB: tuberculosis, PTB: pulmonary tuberculosis, EPTB: extrapulmonary tuberculosis

1. The World Bank Group. World Bank Country and Lending Groups 2020 [Available from: <https://datahelpdesk.worldbank.org/knowledgebase/articles/906519-world-bank-country-and-lending-groups>.
2. World Health Organization. Global tuberculosis report. 2020

Table S2. Study designs of 89 included studies

| **Study design** | **Number of studies** | **%** |
| --- | --- | --- |
| Prospective cohort | 19 | 21.3 |
| Cross sectional | 16 | 18.0 |
| Retrospective cohort | 16 | 18.0 |
| Retrospective observational | 9 | 10.1 |
| Prospective observational | 6 | 6.7 |
| Prospective descriptive | 4 | 4.5 |
| Descriptive survey | 3 | 3.4 |
| Retrospective descriptive | 3 | 3.4 |
| Case control | 3 | 3.4 |
| Observational study | 3 | 3.4 |
| Randomised trial | 3 | 3.4 |
| Retrospective audit | 2 | 2.2 |
| Operations research/interventional | 1 | 1.1 |
| Prospective qualitative | 1 | 1.1 |
