# Supplementary material for: The clinical presentation and detection of tuberculosis during pregnancy and in the postpartum period in low- and middle-income countries: A systematic review and meta-analysis
Source: PLOS Glob Public Health. 2023 Aug 23;3(8):e0002222. doi: 10.1371/journal.pgph.0002222 (PMC10446195; doi:10.1371/journal.pgph.0002222)
Supplement: S6 File — (DOCX) [file pgph.0002222.s006.docx]

**Appendix S6: Characteristics of the studies by country and income-level.**

| **Country** | **Number of studies** | **Number of women included** | **Income level** |
| --- | --- | --- | --- |
| **Africa** | **48** | **1797** |  |
| South Africa | 23 | 1429 | Upper-middle |
| Kenya | 5 | 45 | Lower-middle |
| Nigeria | 4 | 135 | Lower-middle |
| Ethiopia | 4 | 21 | Low |
| Zambia | 2 | 37 | Lower-middle |
| Uganda | 2 | 18 | Low |
| Botswana | 1 | 2 | Upper-middle |
| Lesotho | 1 | 3 | Lower-middle |
| Senegal | 1 | 14 | Lower-middle |
| Sudan | 1 | 42 | Low |
| Togo | 1 | 13 | Low |
| Madagascar | 1 | 24 | Low |
| Eswatini | 1 | 12 | Lower-middle |
| Benin | 1 | 2 | Lower-middle |
| **Asia** | **30** | **973** |  |
| India | 19 | 657 | Lower-middle |
| China | 5 | 100 | Upper-middle |
| Laos | 1 | 2 | Lower-middle |
| Mongolia | 1 | 104 | Lower-middle |
| Papua New Guinea | 1 | 71 | Lower-middle |
| Philippines | 1 | 4 | Lower-middle |
| Pakistan | 1 | 27 | Lower-middle |
| Afghanistan | 1 | 8 | Low |
| **Europe** | **5** | **123** |  |
| Russia | 3 | 120 | Upper-middle |
| Turkey | 2 | 3 | Upper-middle |
| **North America** | **1** | **25** |  |
| Mexico | 1 | 25 | Upper-middle |
| **South America** | **2** | **43** |  |
| Brazil | 1 | 7 | Upper-middle |
| Peru | 1 | 36 | Upper-middle |
| **Multiple** | **2** | **9** |  |
| South Africa, Tanzania, Uganda, and Zimbabwe | 1 | 4 | 1 upper-middle, 2 lower-middle, 1 low |
| Brazil, Mozambique | 1 | 5 | 1 upper-middle, 1 low |
| **Totals** | **89** | **3078** |  |
