# Supplementary material for: The clinical presentation and detection of tuberculosis during pregnancy and in the postpartum period in low- and middle-income countries: A systematic review and meta-analysis
Source: PLOS Glob Public Health. 2023 Aug 23;3(8):e0002222. doi: 10.1371/journal.pgph.0002222 (PMC10446195; doi:10.1371/journal.pgph.0002222)
Supplement: S7 File — (DOCX) [file pgph.0002222.s007.docx]

**Appendix S7. Types of extrapulmonary tuberculosis amongst 374 women, and methods by which they were detected**

| **Type of extrapulmonary TB** | **Number of cases**^a^ |  |
| --- | --- | --- |
| **Total** | **374** |  |
| **Miliary/disseminated** | **62** |  |
| *Detected on* *X-ray, sputum examination, CT scan, bronchoalveolar lavage, lung biopsy, fundus exam, CT, and MRI* | *6* |  |
| *Detected on chest X-ray and CT scan* | *5* |  |
| *Detected on CT scan* | *1* |  |
| *Detected on blood culture* | *1* |  |
| *Detected on radiological imaging* | *1* |  |
| *Detected on chest X-ray* | *1* |  |
| *Not reported* | *47* |  |
| **Genital/genitourinary** | **51** |  |
| *Detected on endometrial biopsy* | *8* |  |
| *Detected on TB culture* | *1* |  |
| *Not reported* | *42* |  |
| **Osteoarticular (bone, joint)** | **48** |  |
| *Detected on X-ray, biopsy, and aspiration* | *3* |  |
| *Detected on bone biopsy* | *1* |  |
| *Detected on X-ray, and biopsy* | *1* |  |
| *Detected on MRI* | *1* |  |
| *Not reported* | *42* |  |
| **Not specified** | **50** |  |
| **Pleural** | **46** |  |
| *Detected on pleural fluid culture* | *6* |  |
| *Not reported* | *40* |  |
| **Meningitis/central nervous system** | **40** |  |
| *Detected on CSF analysis, and CT scan* | *4* |  |
| *Detected on MRI, lumbar puncture, and CT* | *3* |  |
| *Detected on neuroimaging , CSF analysis +/- MGIT* | *2* |  |
| *Detected on* *CSF culture* | *3* |  |
| *Detected on* *CSF analysis* | *2* |  |
| *Detected on CT scan* | *2* |  |
| *Detected on head CT* | *3* |  |
| *Not reported* | *21* |  |
| **Peripheral lymph node** | **32** |  |
| *Detected on fine needle aspirate* | *7* |  |
| *Detected on lymph node culture* | *2* |  |
| *Not reported* | *23* |  |
| **Abdomen** | **12** |  |
| *Detected on biopsy, CT, barium meal follow through* | *3* |  |
| *Detected on lymph node culture* | *1* |  |
| *Not reported* | *8* |  |
| **Renal** | **10** |  |
| *Detected on urinalysis* | *2* |  |
| *Detected on histopathology* | *1* |  |
| *Not reported* | *7* |  |
| **Pericarditis** | **5** |  |
| *Detected on adenosine deaminase testing on pericardial fluid* | *2* |  |
| *Detected on pericardial fluid culture* | *1* |  |
| *Not reported* | *2* |  |

TB: tuberculosis, CT: computerized tomography, MRI: magnetic resonance imaging, CSF: cerebrospinal fluid

1. The total differs from the number of cases in this row as some women had multiple types of extrapulmonary TB.
