# Supplementary material for: The clinical presentation and detection of tuberculosis during pregnancy and in the postpartum period in low- and middle-income countries: A systematic review and meta-analysis
Source: PLOS Glob Public Health. 2023 Aug 23;3(8):e0002222. doi: 10.1371/journal.pgph.0002222 (PMC10446195; doi:10.1371/journal.pgph.0002222)
Supplement: S8 File — (DOCX) [file pgph.0002222.s008.docx]

**Appendix S8: Clinical feature prevalence for 38 clinical features of tuberculosis in pregnancy.**

Table S1. Prevalence of clinical features for tuberculosis in pregnancy, ordered by number of reporting studies.

| **Clinical feature** | **Number of reporting studies** | **Number of studies reporting prevalence** | **Prevalence** | **Confidence interval** | **T^2^** | **Q** | **Q p value** | **PTB only** | **Number of reporting studies** | **Number of studies reporting prevalence** | **Prevalence** | **Confidence interval** | **T^2^** | **Q** | **Q p value** |
| --- | --- | --- | --- | --- | --- | --- | --- | --- | --- | --- | --- | --- | --- | --- | --- |
| Cough | 25 | 22 | 0.68 | 0.53, 0.83 | 0.12 | 1.50x10^12^ | 0.00 |  | 14 | 13 | 0.70 | 0.51, 0.90 | 0.12 | 8.00 x10^11^ | 0.00 |
| Prior TB history | 22 | 20 | 0.31 | 0.19, 0.43 | 0.07 | 5.00 x10^11^ | 0.00 |  | 13 | 12 | 0.27 | 0.10, 0.44 | 0.09 | 6.67 x10^11^ | 0.00 |
| Fever | 21 | 18 | 0.46 | 0.29, 0.63 | 0.13 | 1.33 x10^12^ | 0.00 |  | 9 | 9 | 0.25 | 0.11, 0.39 | 0.04 | 87.27 | 0.00 |
| Weight loss/absence of weight gain | 17 | 14 | 0.28 | 0.13, 0.43 | 0.07 | 286.08 | 0.00 |  | 10 | 9 | 0.35 | 0.14, 0.56 | 0.10 | 247.07 | 0.00 |
| Night sweats | 10 | 10 | 0.30 | 0.14, 0.47 | 0.06 | 205.23 | 0.00 |  | 8 | 8 | 0.31 | 0.11, 0.51 | 0.08 | 186.98 | 0.00 |
| History of close TB contact | 9 | 9 | 0.23 | 0.12, 0.34 | 0.02 | 83.25 | 0.00 |  | 5 | 5 | 0.23 | 0.05, 0.41 | 0.03 | 35.35 | 0.00 |
| Haemoptysis | 8 | 6 | 0.13 | 0.00, 0.26 | 0.02 | 33.59 | 0.00 |  | 5 | 5 | 0.13 | 0.00, 0.28 | 0.02 | 32.43 | 0.00 |
| Sputum production | 7 | 7 | 0.73 | 0.57, 0.89 | 0.03 | 52.99 | 0.00 |  | 5 | 5 | 0.69 | 0.54, 0.85 | 0.02 | 13.30 | 0.01 |
| Shortness of breath | 7 | 6 | 0.54 | 0.27, 0.81 | 0.10 | 342.12 | 0.00 |  | 3 | 3 | 0.44 | 0.05, 0.83 | 0.11 | 48.44 | 0.00 |
| History of known TB exposure | 7 | 5 | 0.45 | 0.31, 0.59 | 0.01 | 6.54 | 0.16 |  | 6 | 5 | 0.45 | 0.31, 0.59 | 0.01 | 6.54 | 0.16 |
| Asymptomatic | 6 | 5 | 0.58 | 0.23,0.93 | 0.14 | 727.50 | 0.00 |  | 5 | 5 | 0.58 | 0.23,0.93 | 0.14 | 727.50 | 0.00 |
| Fatigue | 6 | 5 | 0.48 | 0.14, 0.82 | 0.13 | 497.47 | 0.00 |  | 2 | 2 | 0.46 | 0.20, 0.72 | 0.02 | 1.73 | 0.19 |
| Headache | 5 | 3 | 0.23 | 0.05, 0.41 | 0.00 | 0.83 | 0.66 |  | 0 | 0 |  |  |  |  |  |
| Loss of appetite | 4 | 3 | 0.50 | 0.15, 0.85 | 0.08 | 16.57 | 0.00 |  | 2 | 2 | 0.44 | 0.00, 0.94 | 0.13 | 15.18 | 0.00 |
| Lymphadenopathy | 4 | 2 | 0.01 | 0.00, 0.08 | 0.00 | 1.17 | 0.28 |  | 2 | 2 | 0.01 | 0.00, 0.08 | 0.00 | 1.17 | 0.28 |
| Chest pain | 3 | 2 | 0.35 | 0.20, 0.49 | 0.00 | 0.42 | 0.52 |  | 2 | 2 | 0.35 | 0.20, 0.49 | 0.00 | 0.42 | 0.52 |
| Altered sensorium | 3 | 2 | 0.28 | 0.02, 0.53 | 0.01 | 1.18 | 0.28 |  | 0 | 0 |  |  |  |  |  |
| Seizures | 3 | 1 | 1.00 ^b^ | 0.16, 1.00^a^ |  |  |  |  | 0 | 0 |  |  |  |  |  |
| Malaise | 3 | 1 | 0.06 ^b^ | 0.01, 0.21 |  |  |  |  | 0 | 0 |  |  |  |  |  |
| Vaginal bleeding | 2 | 2 | 0.63 | 0.37, 0.88 | 0.00 | 0.65 | 0.42 |  | 0 | 0 |  |  |  |  |  |
| Chills | 2 | 1 | 0.24 ^b^ | 0.07, 0.50 |  |  |  |  | 1 | 1 | 0.24 ^b^ | 0.07, 0.50 |  |  |  |
| Ankle swelling | 2 | 0 |  |  |  |  |  |  | 0 | 0 |  |  |  |  |  |
| Bone and joint pain | 1 | 1 | 1.00 ^b^ | 0.91, 1.00^a^ |  |  |  |  | 0 | 0 |  |  |  |  |  |
| Fertility issues | 1 | 1 | 1.00 ^b^ | 0.84, 1.00^a^ |  |  |  |  | 0 | 0 |  |  |  |  |  |
| Swelling | 1 | 1 | 0.20 ^b^ | 0.10, 0.34 |  |  |  |  | 0 | 0 |  |  |  |  |  |
| Dizziness | 1 | 1 | 0.14 ^b^ | 0.00, 0.58 |  |  |  |  | 0 | 0 |  |  |  |  |  |
| Vomiting | 1 | 1 | 0.14 ^b^ | 0.00, 0.58 |  |  |  |  | 0 | 0 |  |  |  |  |  |
| Diplopia | 1 | 1 | 0.14 ^b^ | 0.00, 0.58 |  |  |  |  | 0 | 0 |  |  |  |  |  |
| Confusion | 1 | 0 |  |  |  |  |  |  | 0 | 0 |  |  |  |  |  |
| Paraparesis | 1 | 0 |  |  |  |  |  |  | 0 | 0 |  |  |  |  |  |
| Agitation | 1 | 0 |  |  |  |  |  |  | 0 | 0 |  |  |  |  |  |
| Kyphoscoliosis | 1 | 0 |  |  |  |  |  |  | 0 | 0 |  |  |  |  |  |
| Neck rigidity | 1 | 0 |  |  |  |  |  |  | 0 | 0 |  |  |  |  |  |
| Epistaxis | 1 | 0 |  |  |  |  |  |  | 0 | 0 |  |  |  |  |  |
| Diarrhoea | 1 | 0 |  |  |  |  |  |  | 0 | 0 |  |  |  |  |  |
| Melaena | 1 | 0 |  |  |  |  |  |  | 0 | 0 |  |  |  |  |  |
| Abdominal distension | 1 | 0 |  |  |  |  |  |  | 0 | 0 |  |  |  |  |  |
| Postpartum metrorrhagia | 1 | 0 |  |  |  |  |  |  | 0 | 0 |  |  |  |  |  |

TB: tuberculosis

1. One-sided, 97.5% confidence interval.
2. Meta-analysis unable to be performed due to lack of studies reporting prevalence
