# Supplementary material for: The clinical presentation and detection of tuberculosis during pregnancy and in the postpartum period in low- and middle-income countries: A systematic review and meta-analysis
Source: PLOS Glob Public Health. 2023 Aug 23;3(8):e0002222. doi: 10.1371/journal.pgph.0002222 (PMC10446195; doi:10.1371/journal.pgph.0002222)

**Appendix S9. Meta-analyses of clinical features of tuberculosis in pregnancy and postpartum**

Figure 1. Forest plot of pooled meta-analysis of prevalence of cough in pregnant and up to six months postpartum women with tuberculosis.


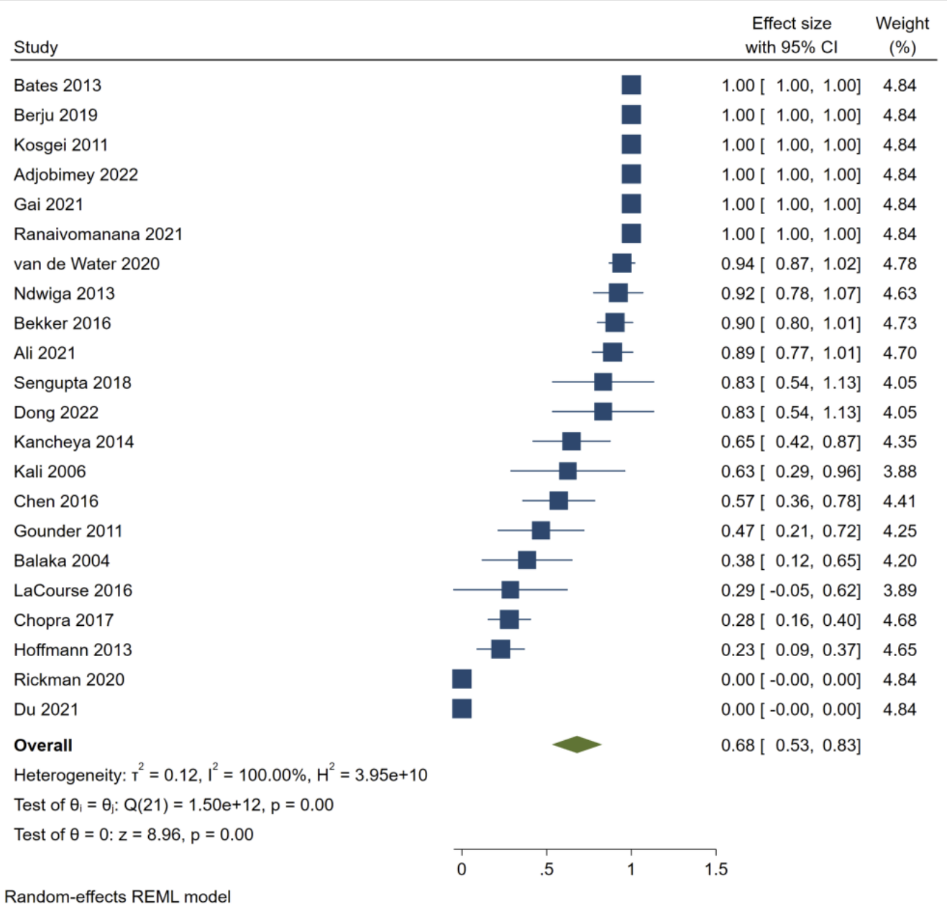


Figure 2. Forest plot of pooled meta-analysis of prevalence of cough in pregnant and up to six months postpartum women with pulmonary tuberculosis.


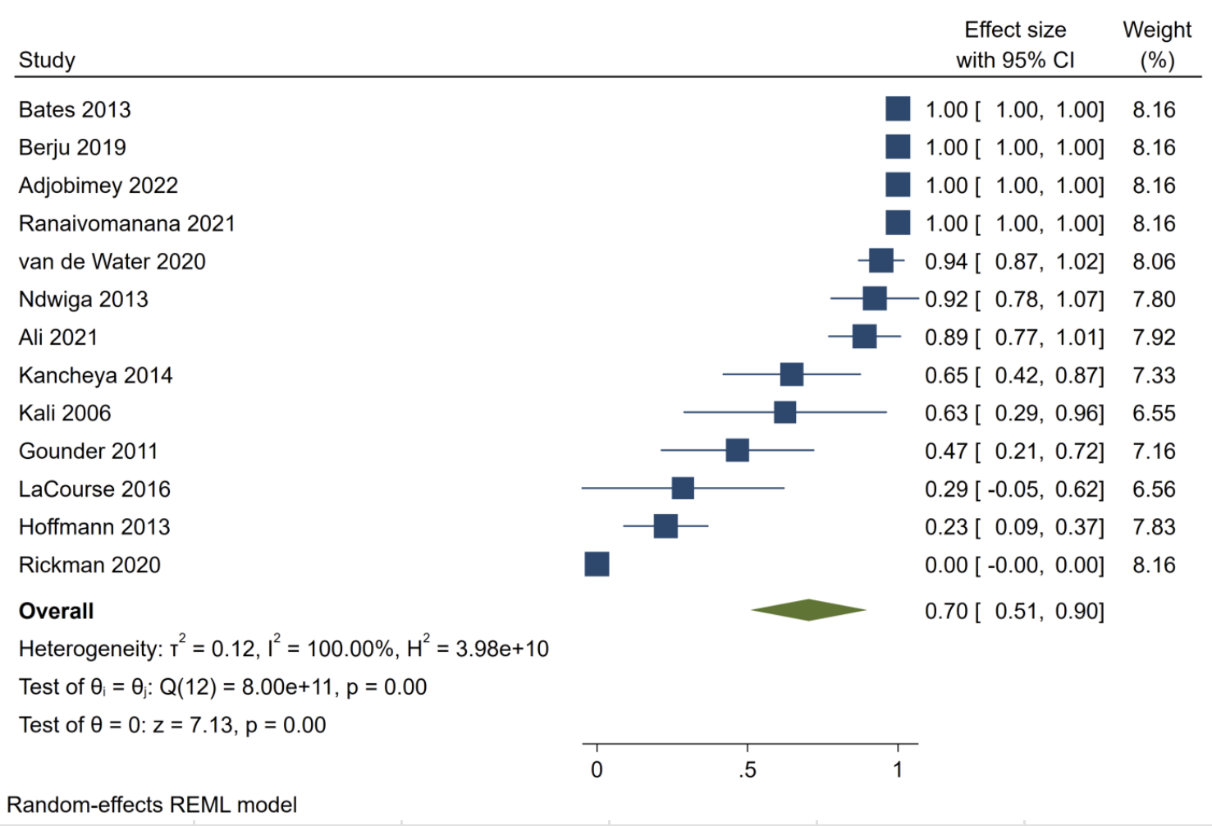


Figure 3. Forest plot of pooled meta-analysis of prevalence of fever in pregnant and up to six months postpartum women with tuberculosis.


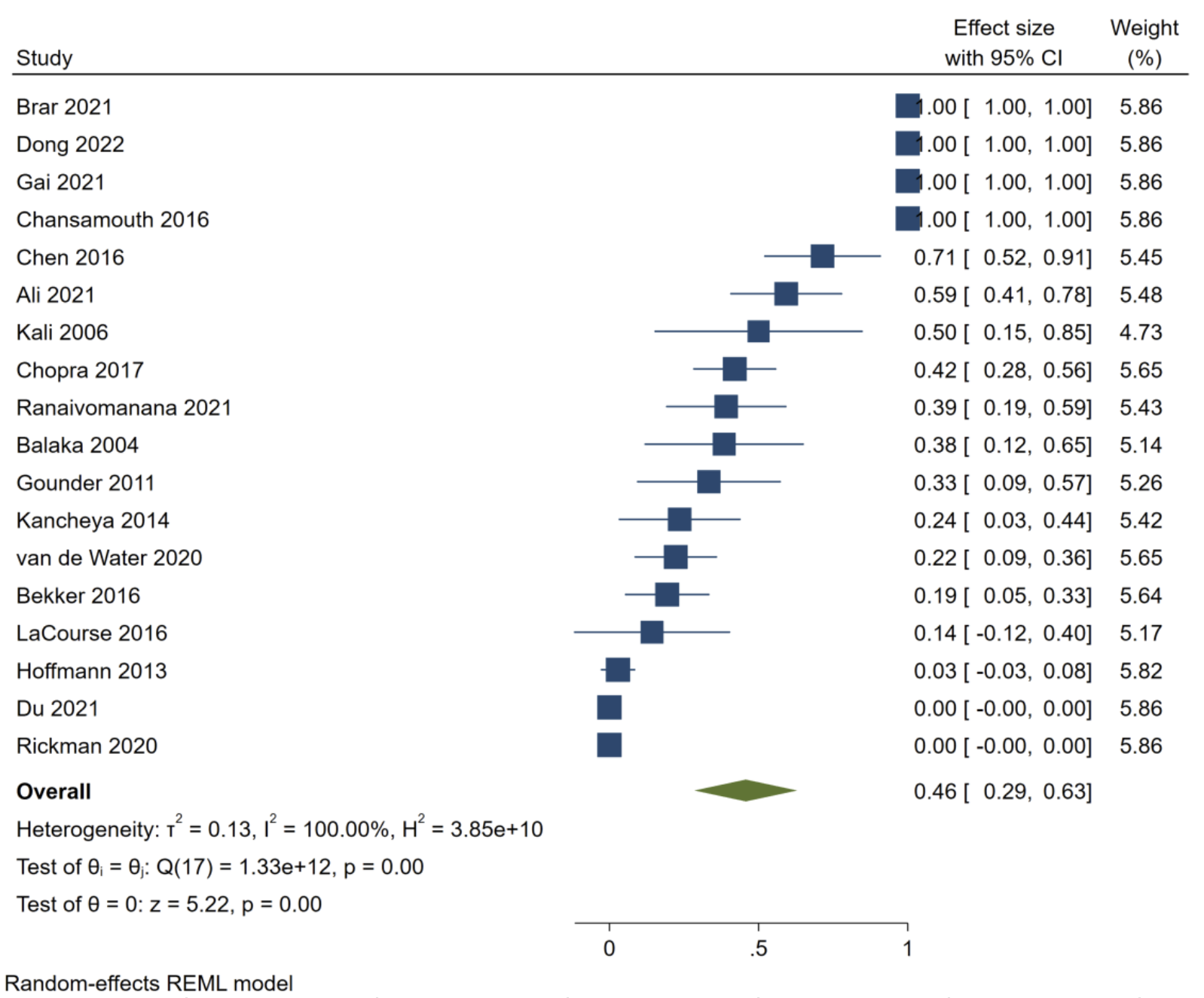


Figure 4. Forest plot of pooled meta-analysis of prevalence of fever in pregnant and up to six months postpartum women with pulmonary tuberculosis.


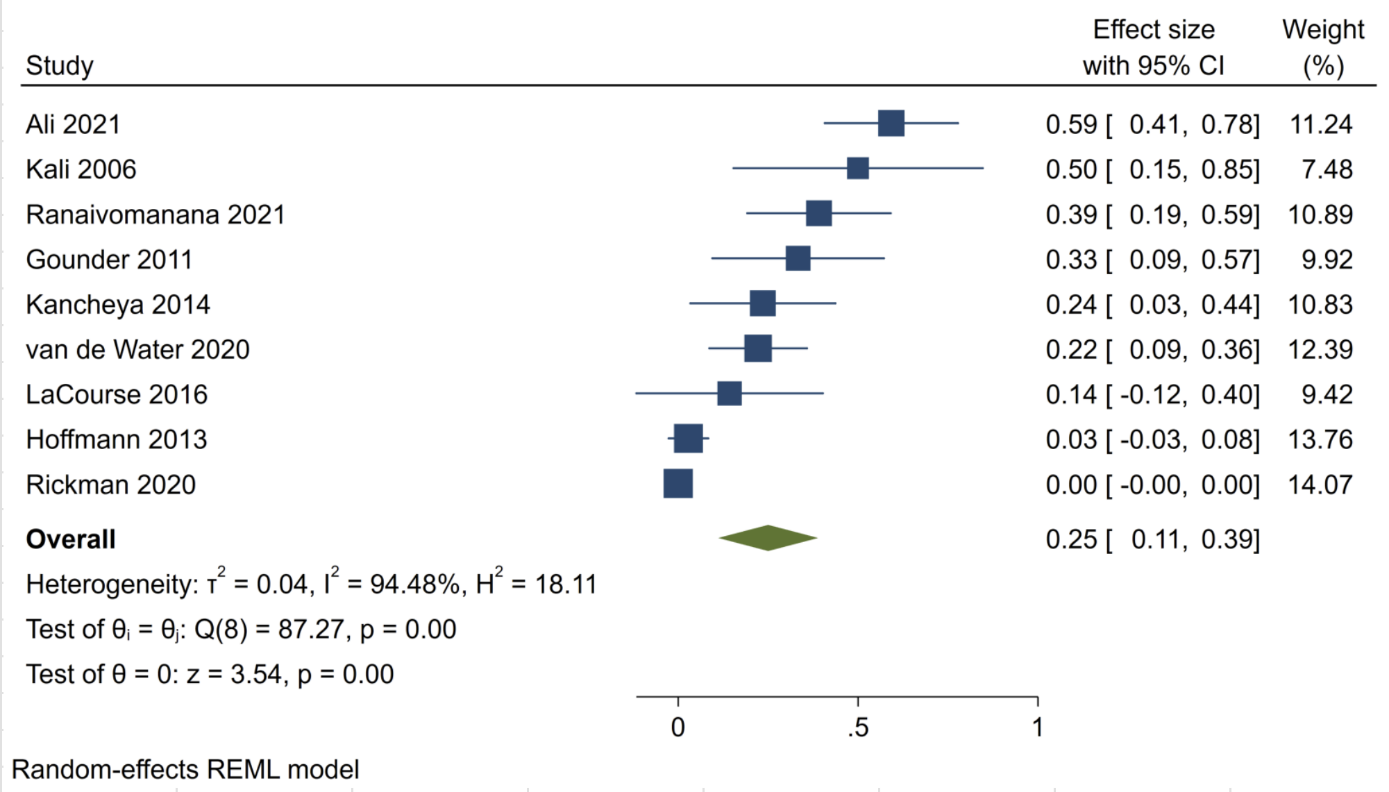


Figure 5. Forest plot of pooled meta-analysis of prevalence of weight loss in pregnant and up to six months postpartum women with tuberculosis.


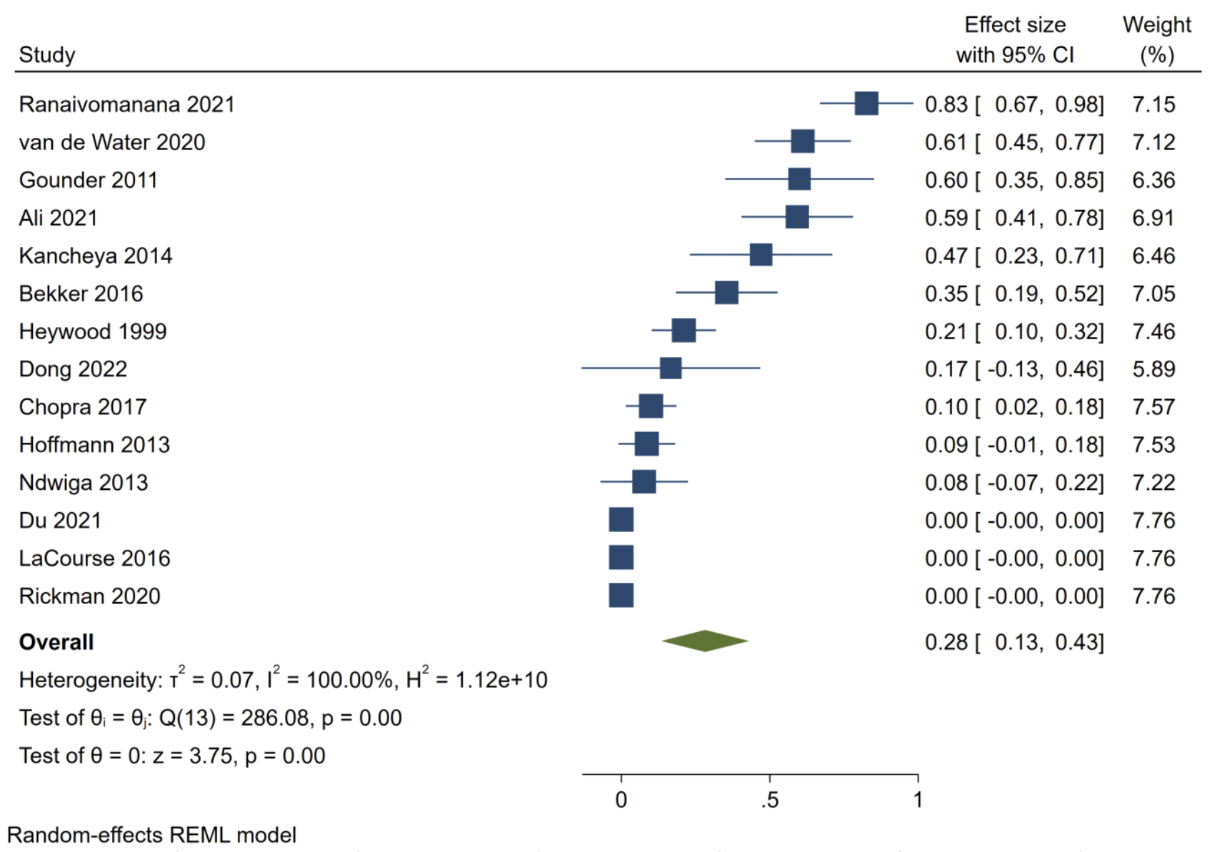


Figure 6. Forest plot of pooled meta-analysis of prevalence of weight loss in pregnant and up to six months postpartum women with pulmonary tuberculosis.


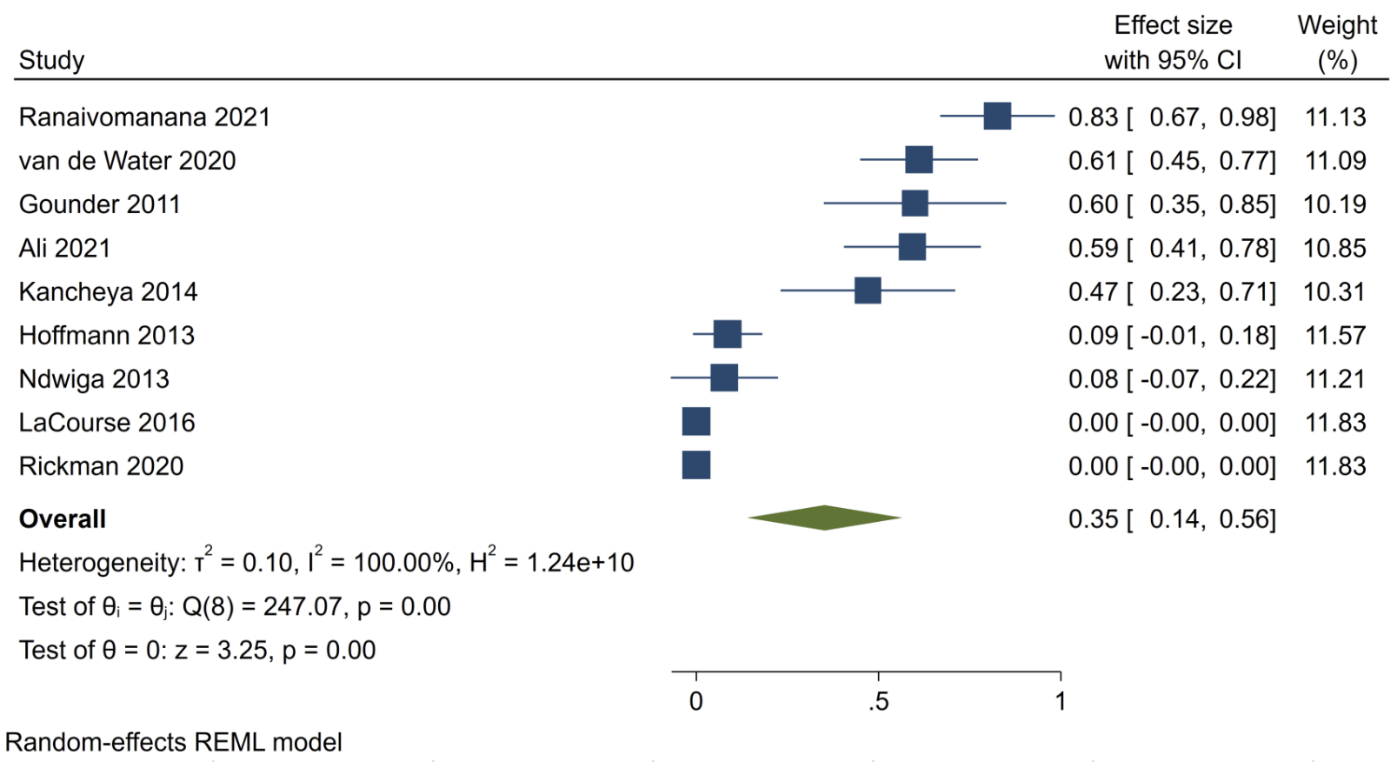


Figure 7. Forest plot of pooled meta-analysis of prevalence of sputum production in pregnant and up to six months postpartum women with tuberculosis.


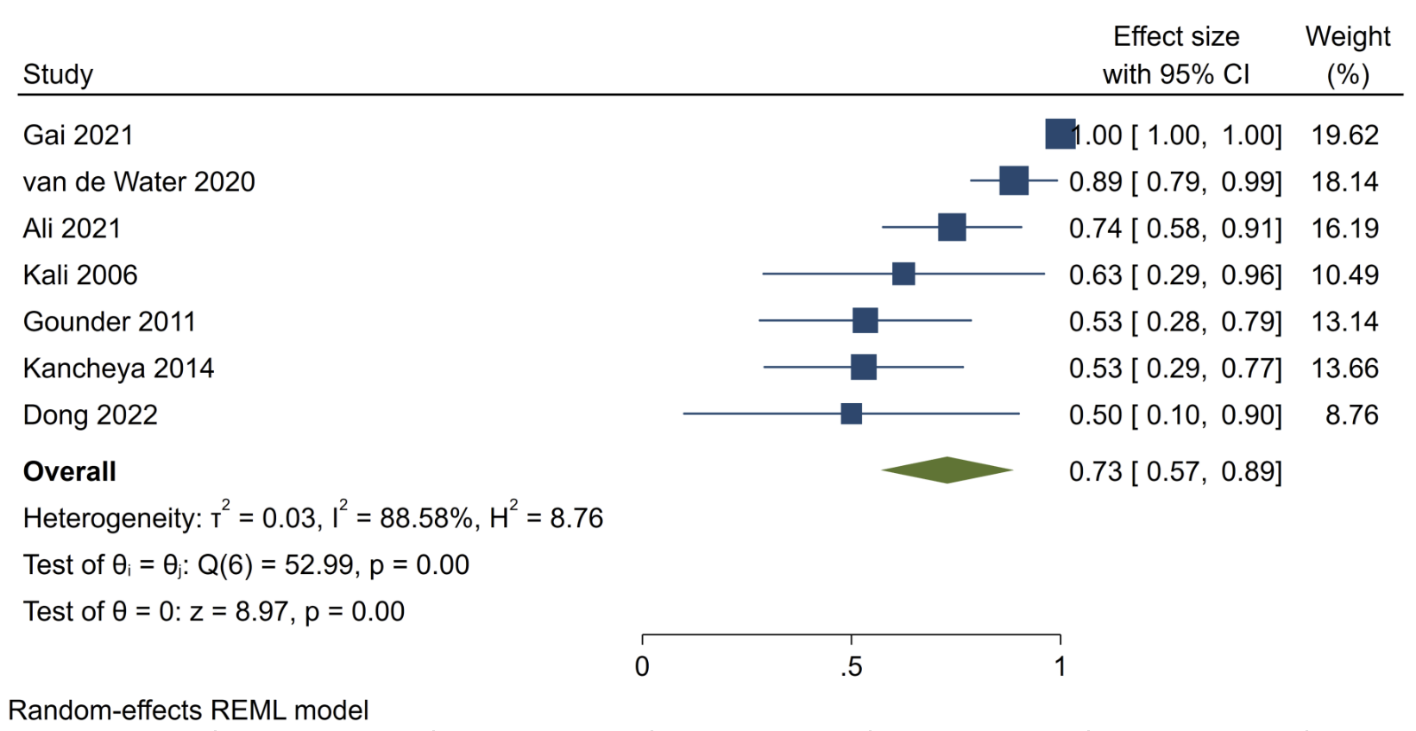


Figure 8. Forest plot of pooled meta-analysis of prevalence of sputum production in pregnant and up to six months postpartum women with pulmonary tuberculosis.


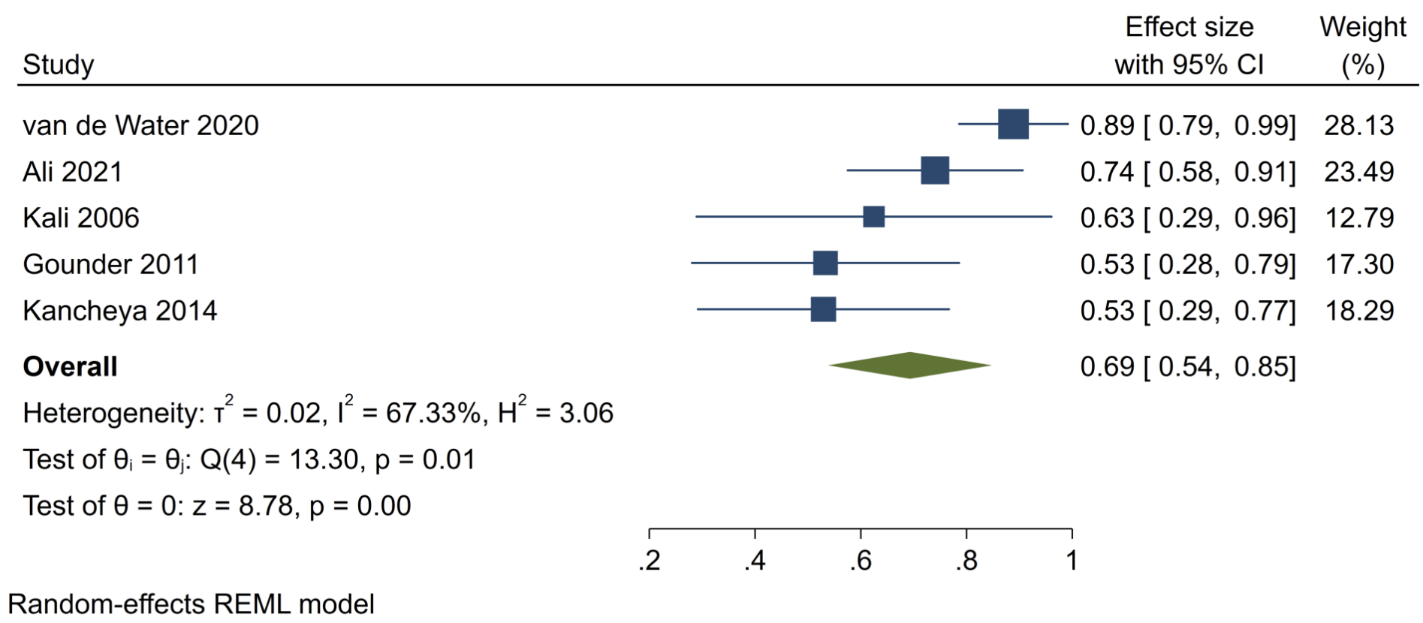


Figure 9. Forest plot of pooled meta-analysis of prevalence of night sweats in pregnant and up to six months postpartum women with tuberculosis.


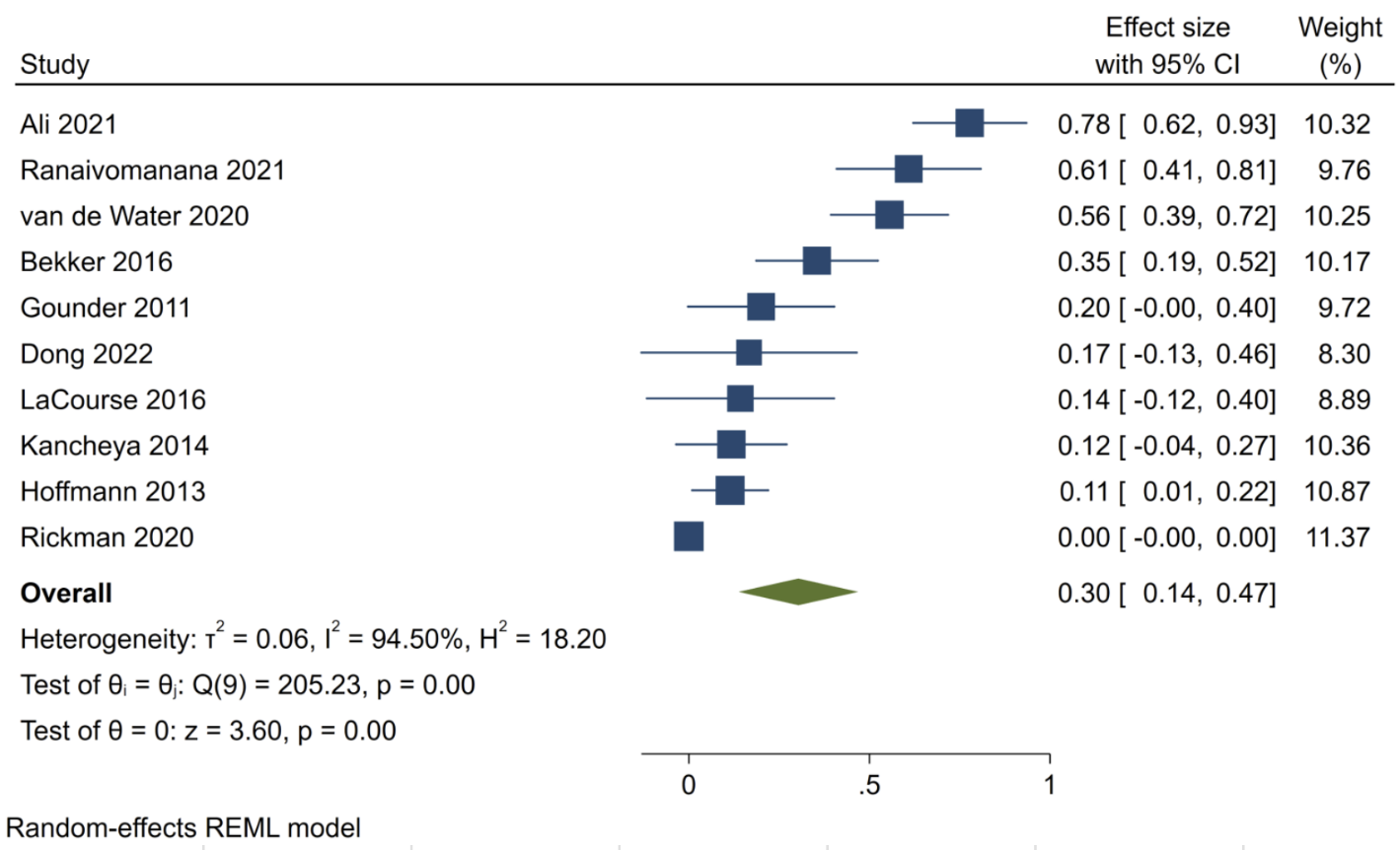


Figure 10. Forest plot of pooled meta-analysis of prevalence of night sweats in pregnant and up to six months postpartum women with pulmonary tuberculosis.


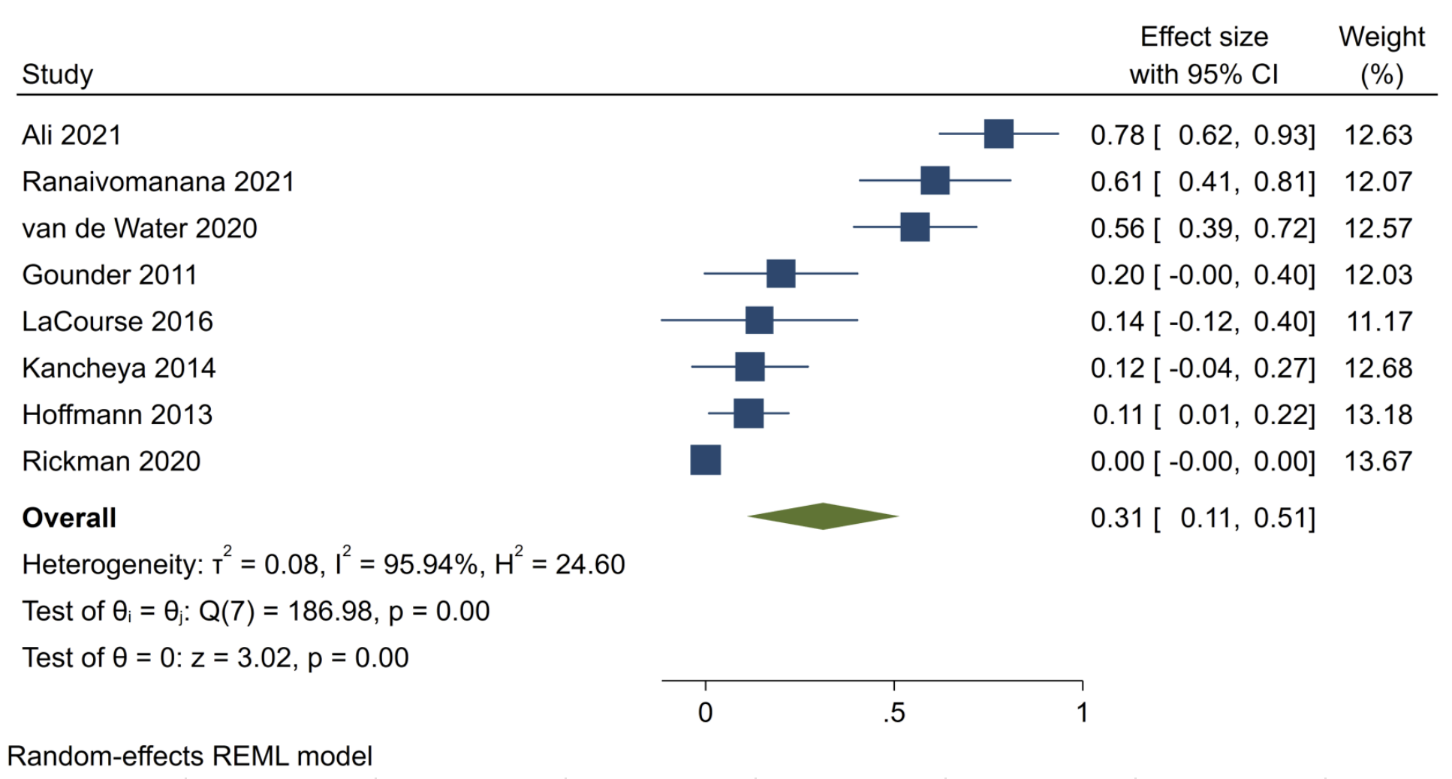


Figure 11. Forest plot of pooled meta-analysis of prevalence of haemoptysis in pregnant and up to six months postpartum women with tuberculosis.


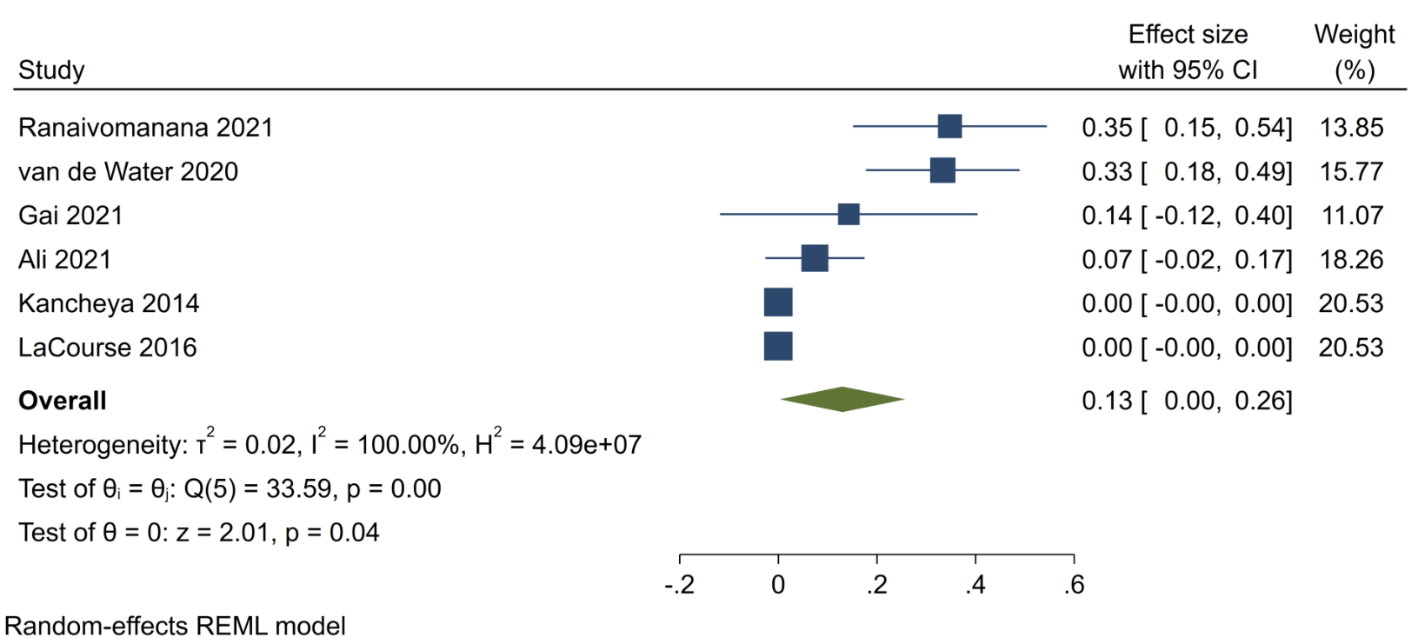


Figure 12. Forest plot of pooled meta-analysis of prevalence of haemoptysis in pregnant and up to six months postpartum women with pulmonary tuberculosis.


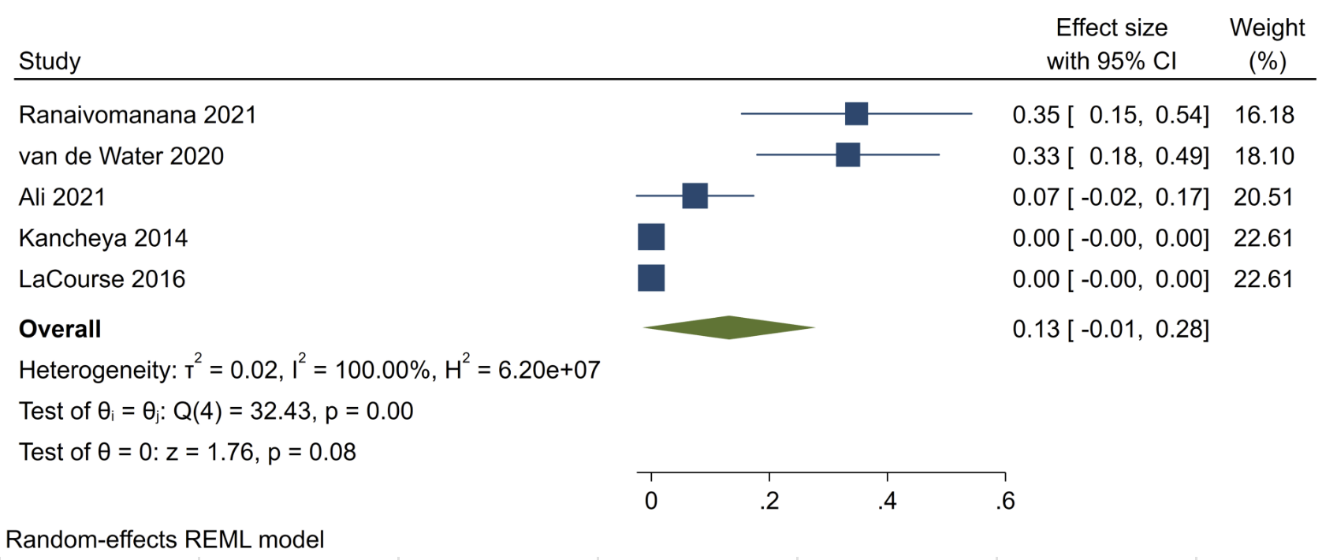


Figure 13. Forest plot of pooled meta-analysis of prevalence of shortness of breath in pregnant and up to six months postpartum women with tuberculosis.


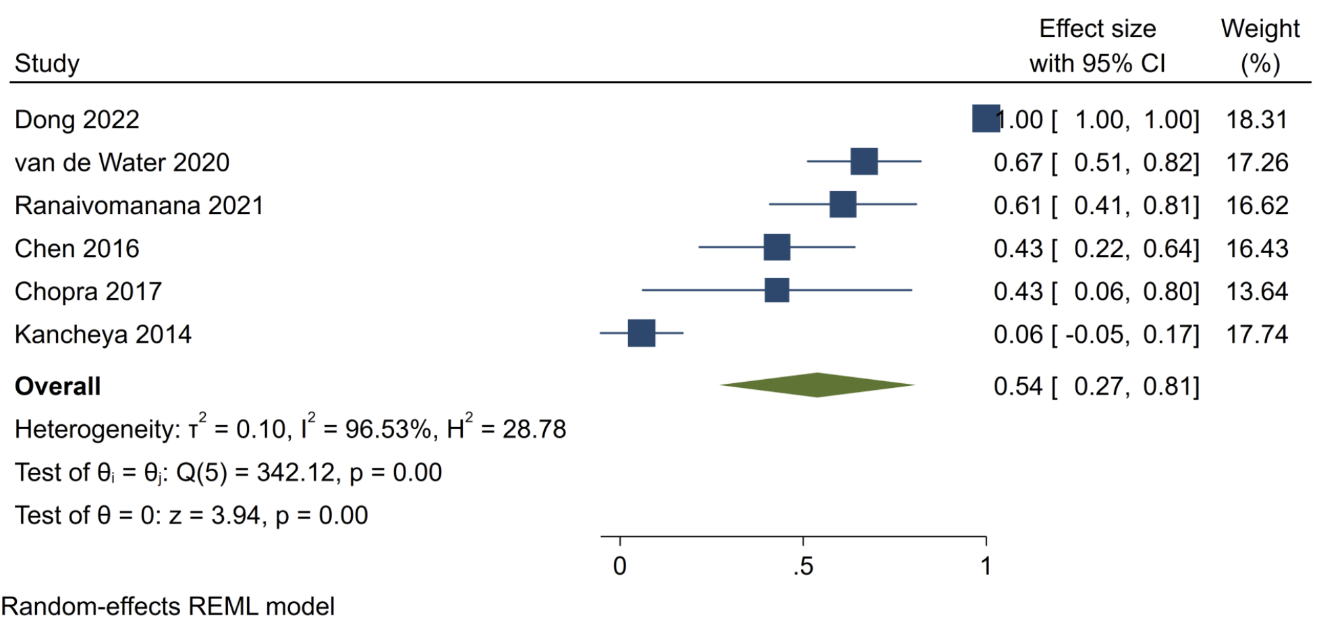


Figure 14. Forest plot of pooled meta-analysis of prevalence of shortness of breath in pregnant and up to six months postpartum women with pulmonary tuberculosis.


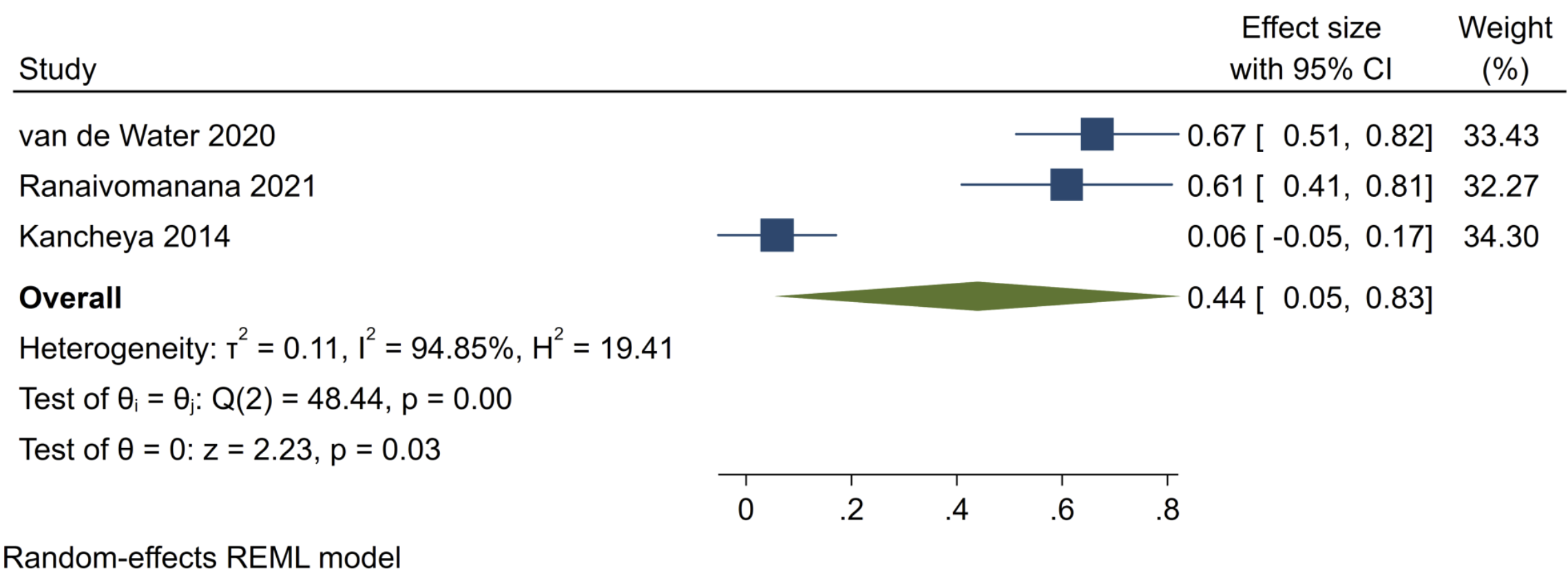


Figure 15. Forest plot of pooled meta-analysis of prevalence of chest pain in pregnant and up to six months postpartum women with tuberculosis.


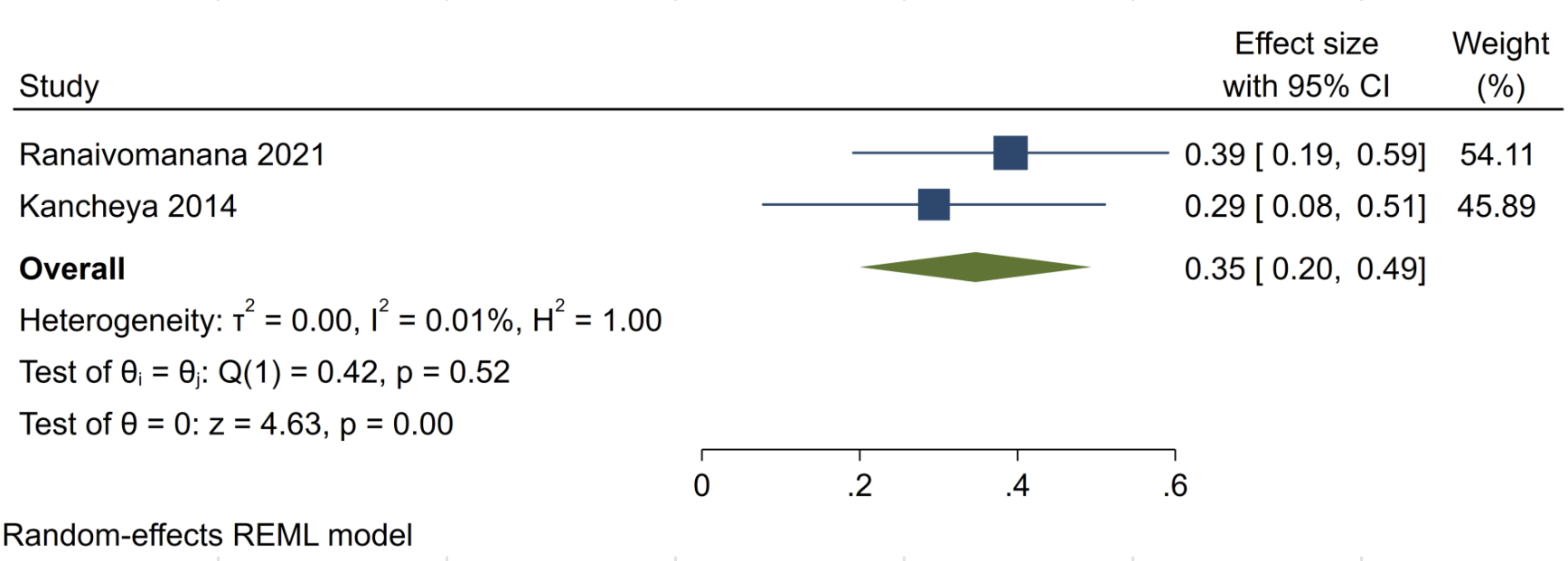


Figure 16. Forest plot of pooled meta-analysis of prevalence of chest pain in pregnant and up to six months postpartum women with pulmonary tuberculosis.


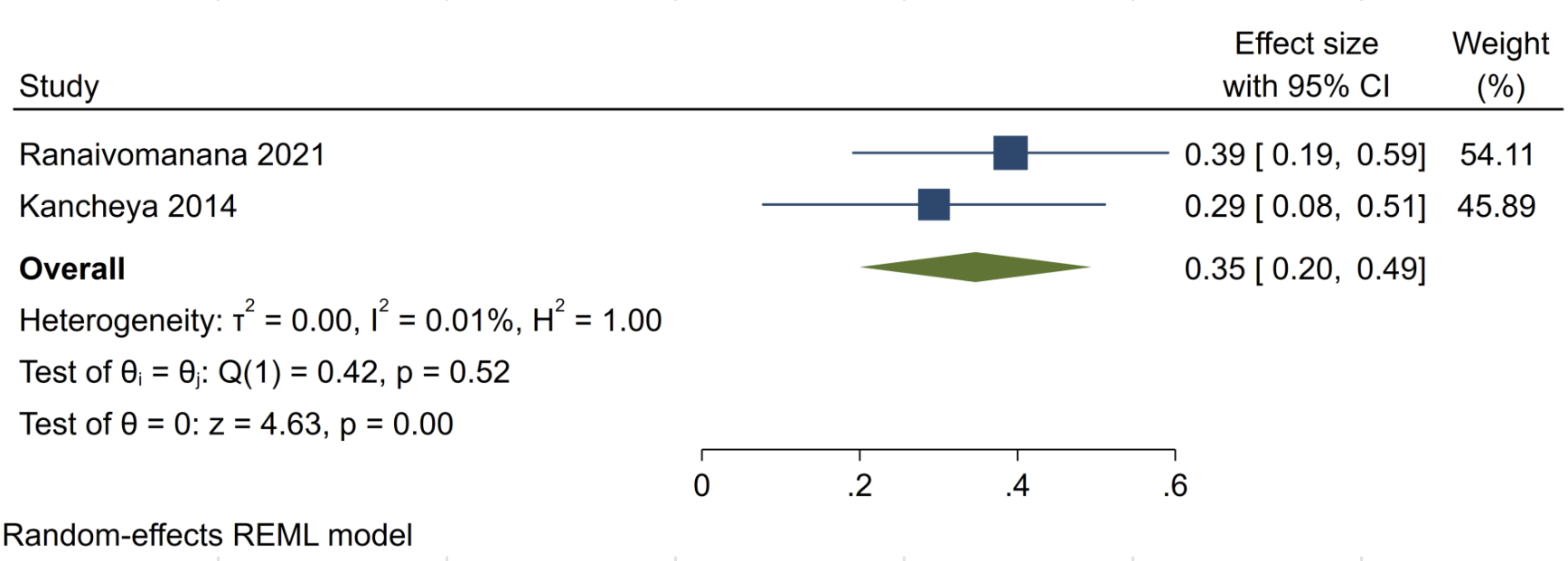


Figure 17. Forest plot of pooled meta-analysis of prevalence of headache in pregnant and up to six months postpartum women with tuberculosis.


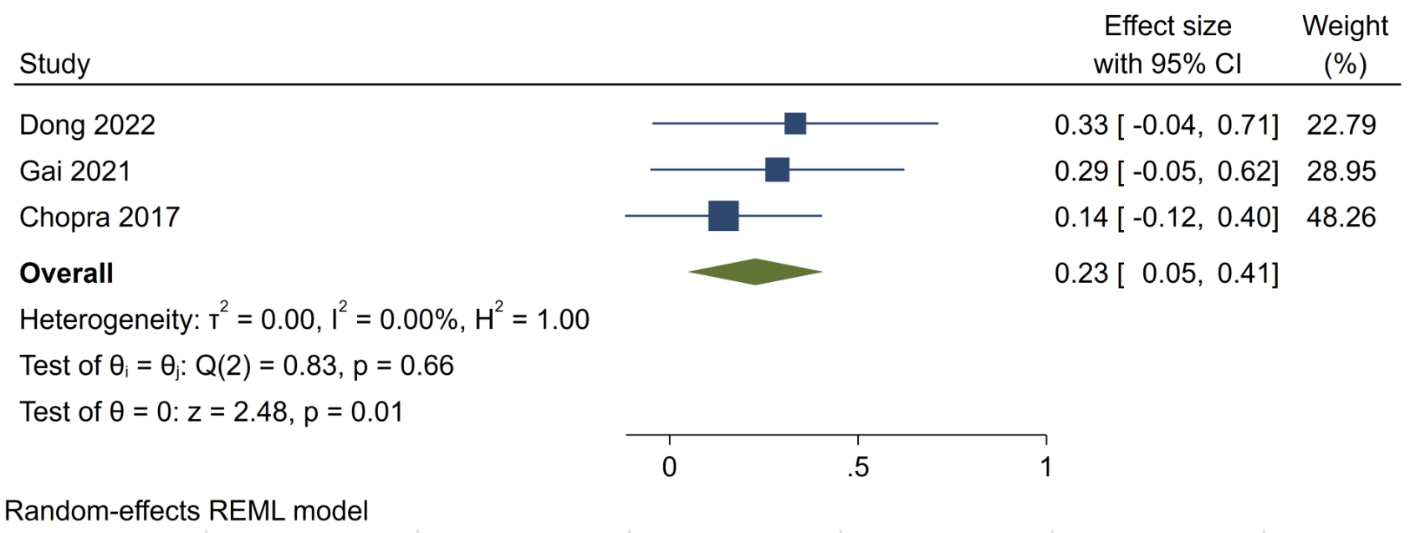


Figure 18. Forest plot of pooled meta-analysis of prevalence of fatigue in pregnant and up to six months postpartum women with tuberculosis.


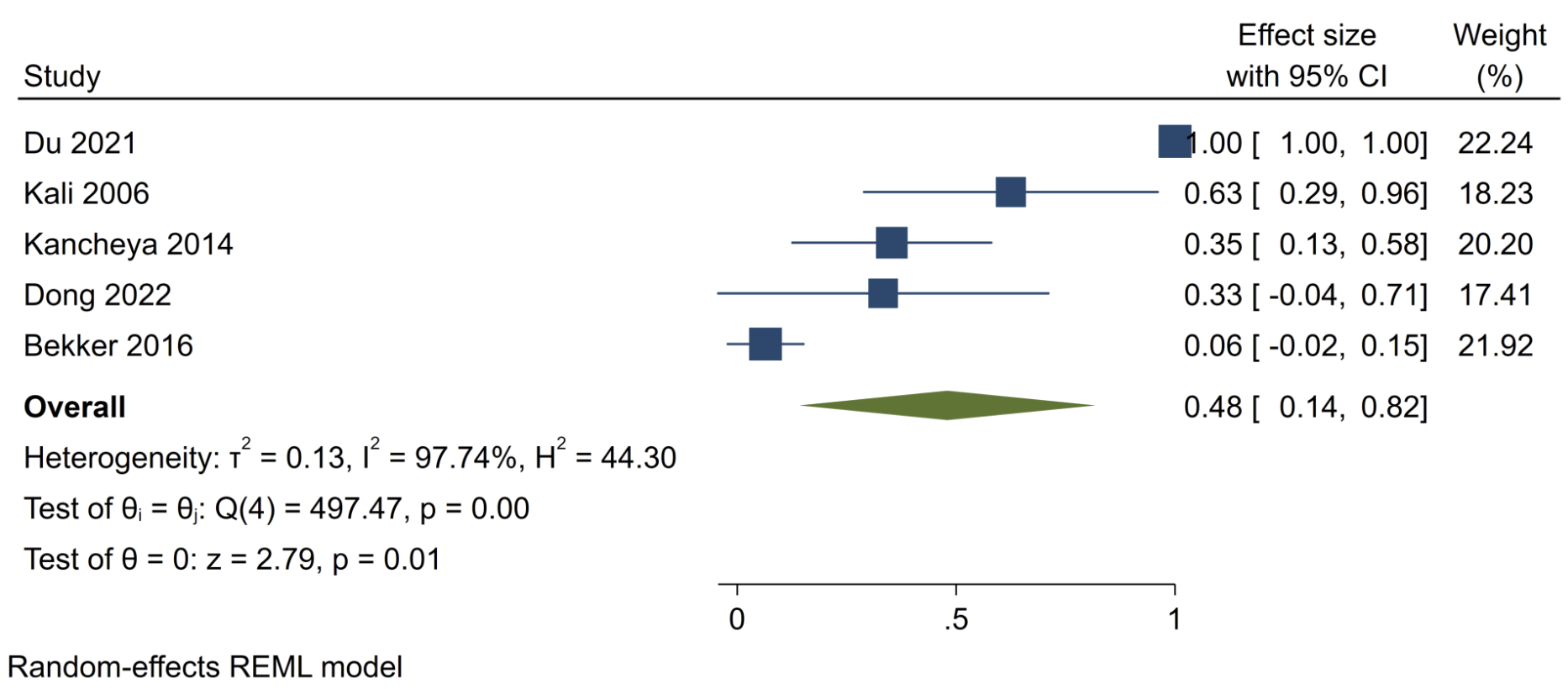


Figure 19. Forest plot of pooled meta-analysis of prevalence of fatigue in pregnant and up to six months postpartum women with pulmonary tuberculosis.


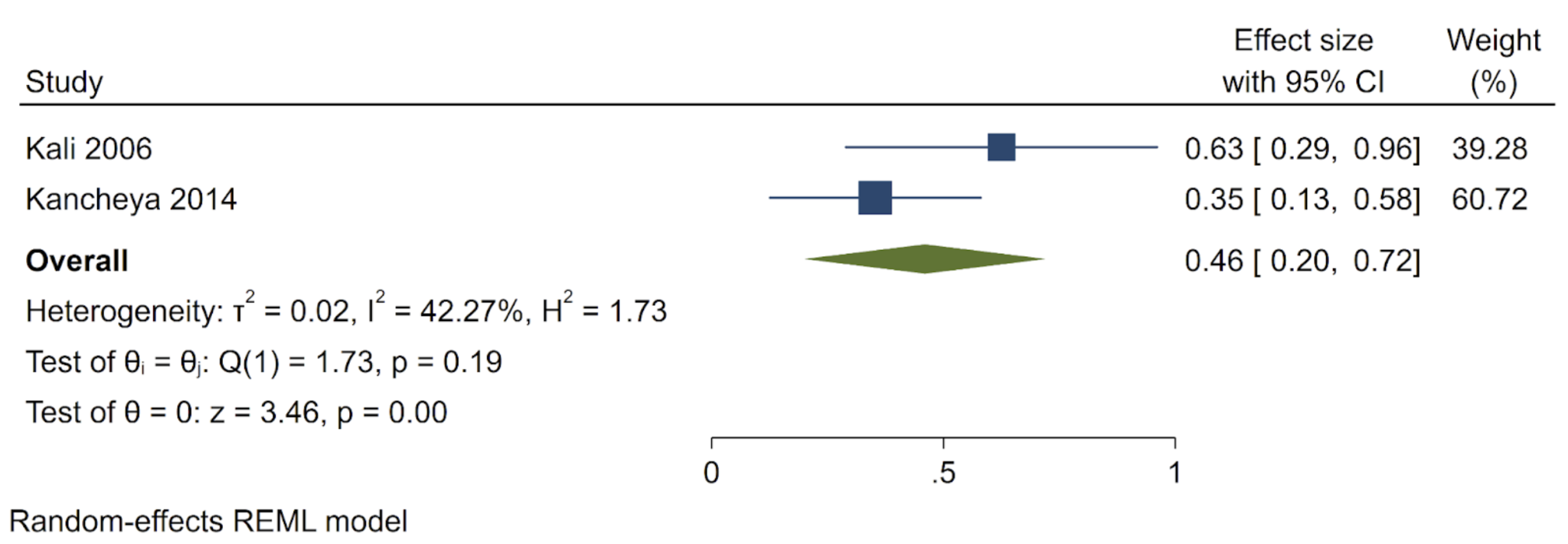


Figure 20. Forest plot of pooled meta-analysis of prevalence of loss of appetite in pregnant and up to six months postpartum women with tuberculosis.


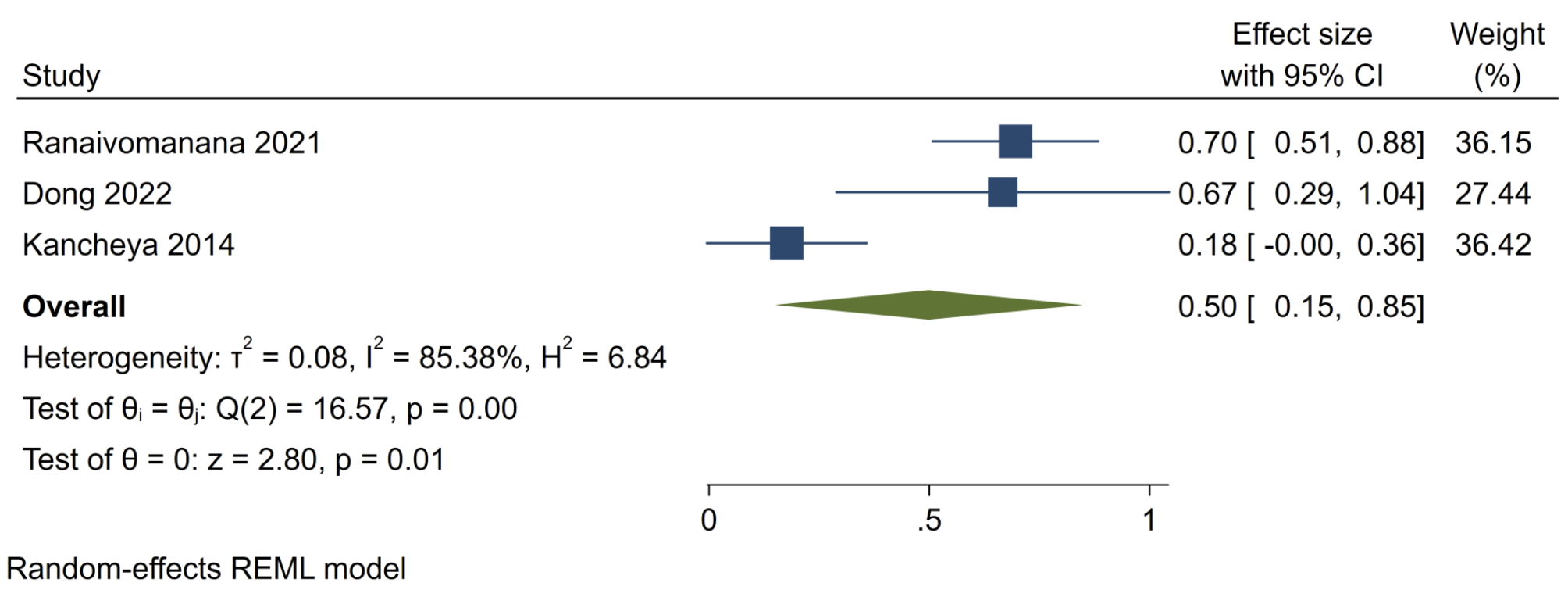


Figure 21. Forest plot of pooled meta-analysis of prevalence of loss of appetite in pregnant and up to six months postpartum women with pulmonary tuberculosis.


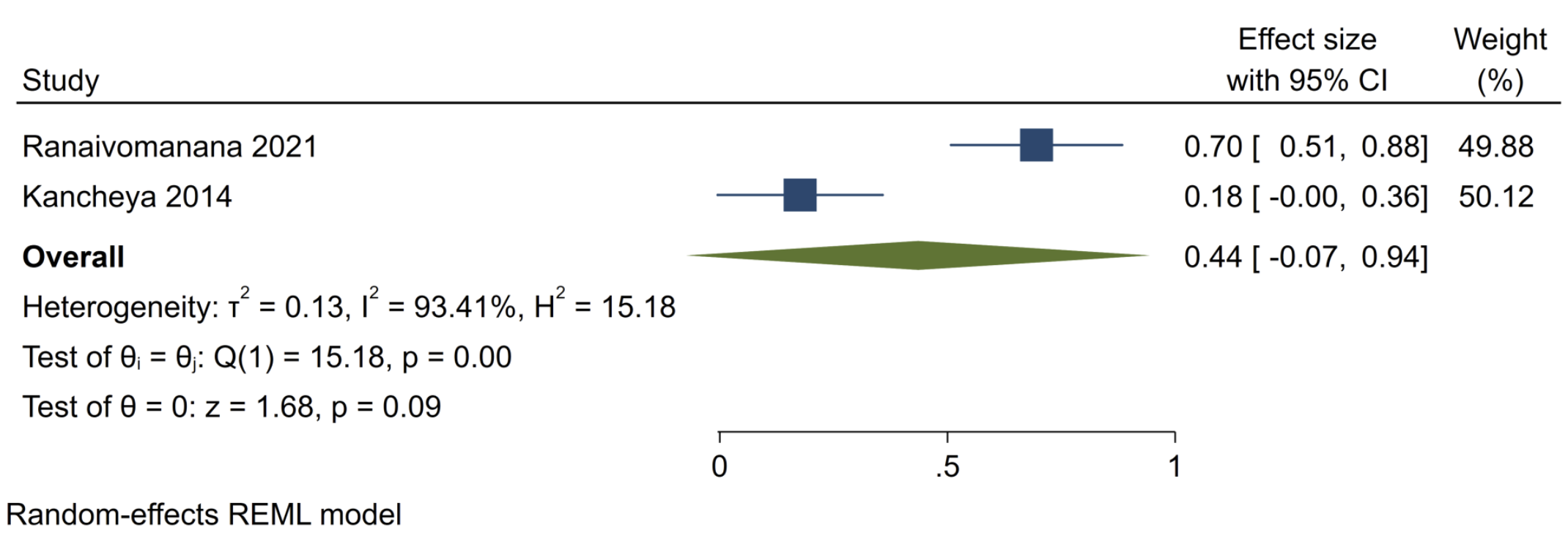


Figure 22. Forest plot of pooled meta-analysis of prevalence of lymphadenopathy in pregnant and up to six months postpartum women with tuberculosis.


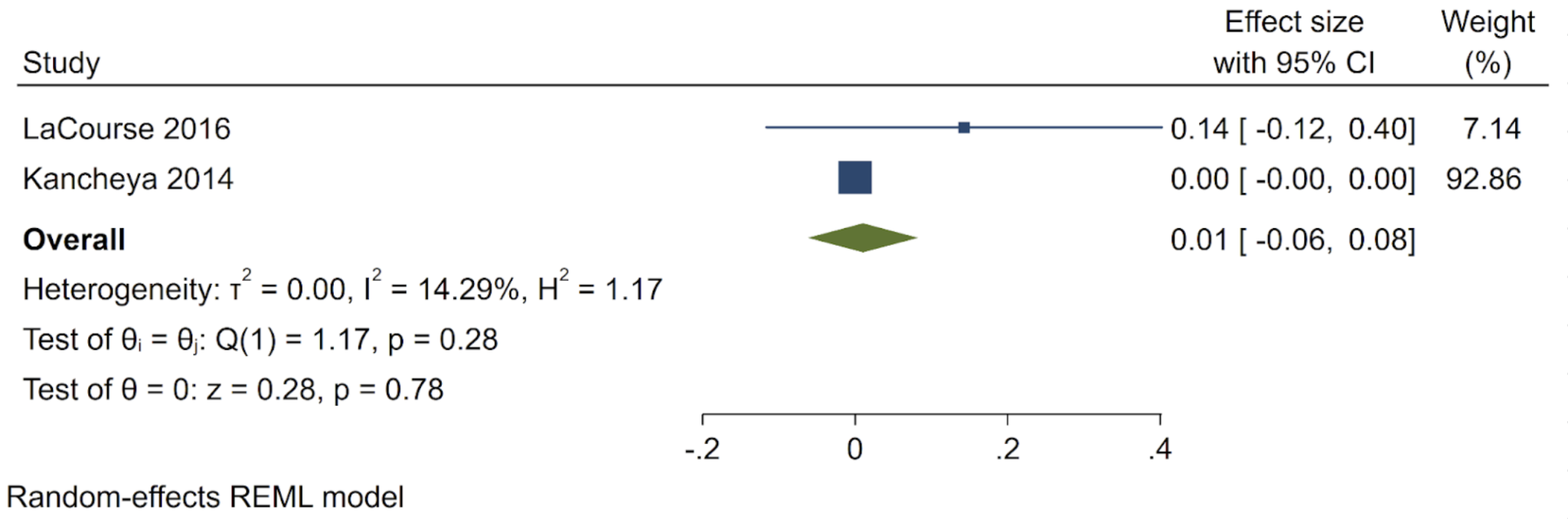


Figure 23. Forest plot of pooled meta-analysis of prevalence of lymphadenopathy in pregnant and up to six months postpartum women with pulmonary tuberculosis.


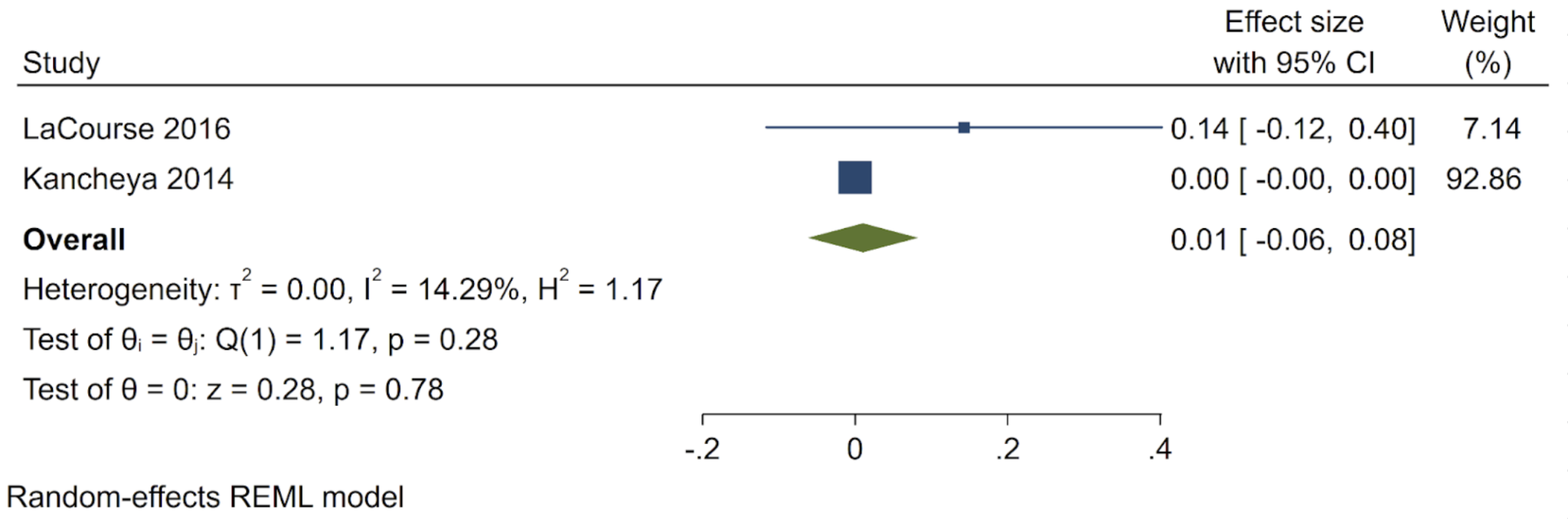


Figure 24. Forest plot of pooled meta-analysis of prevalence of altered sensorium in pregnant and up to six months postpartum women with tuberculosis.


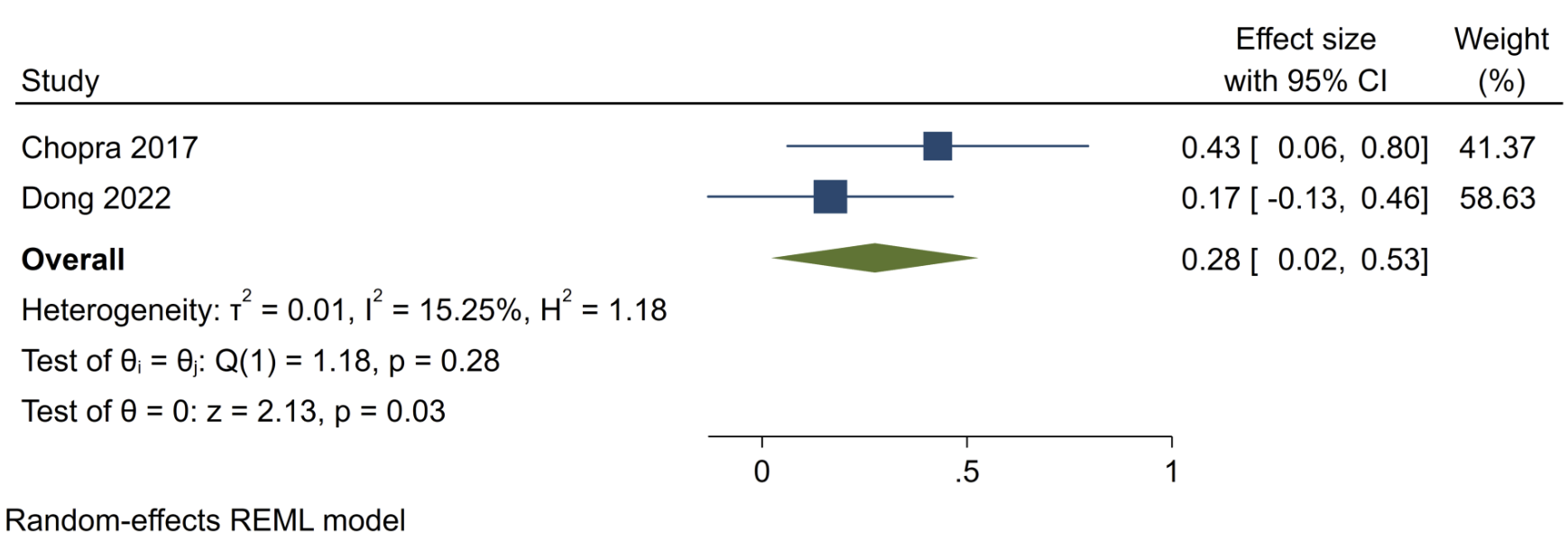


Figure 25. Forest plot of pooled meta-analysis of prevalence of vaginal bleeding in pregnant and up to six months postpartum women with tuberculosis.


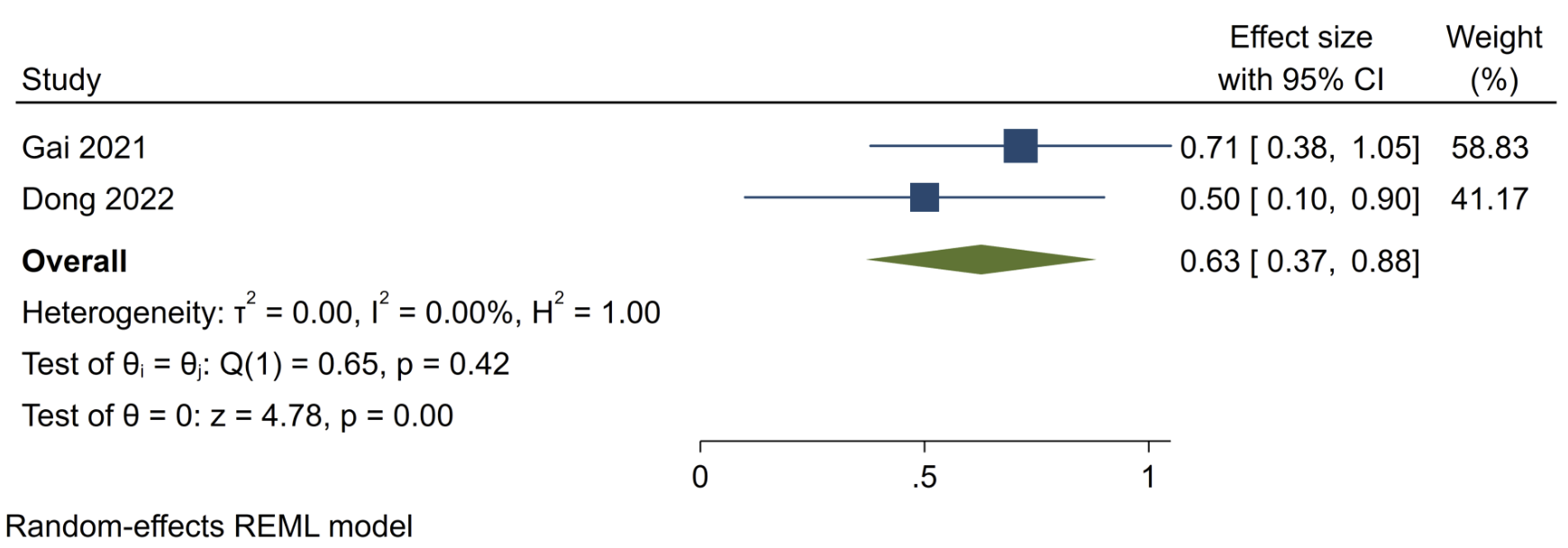


Figure 26. Forest plot of pooled meta-analysis of prevalence of being asymptomatic in pregnant and up to six months postpartum women with tuberculosis.


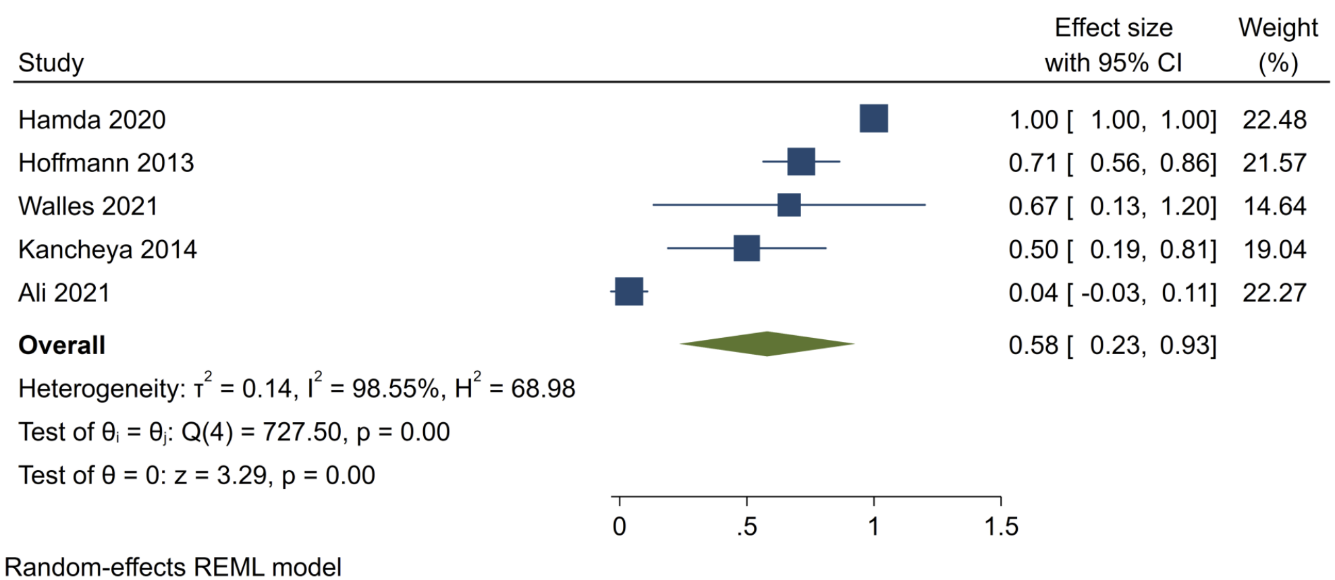


Figure 27. Forest plot of pooled meta-analysis of prevalence of being asymptomatic in pregnant and up to six months postpartum women with pulmonary tuberculosis.


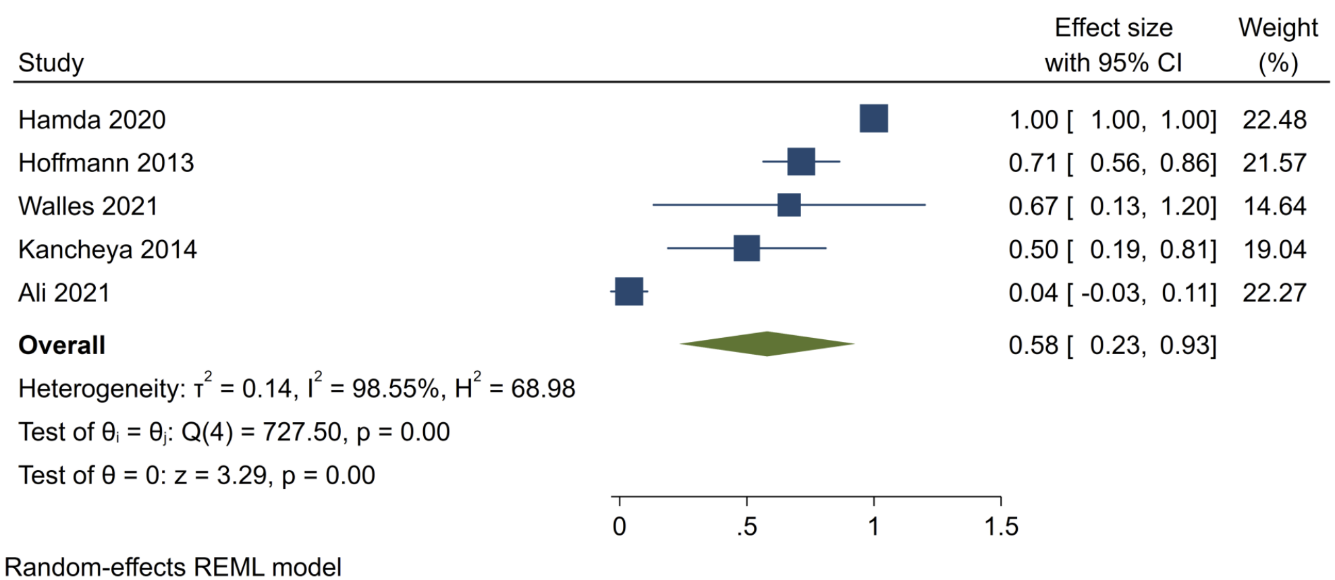


Figure 28. Forest plot of pooled meta-analysis of prevalence of having a prior history of tuberculosis in pregnant and up to six months postpartum women with tuberculosis.


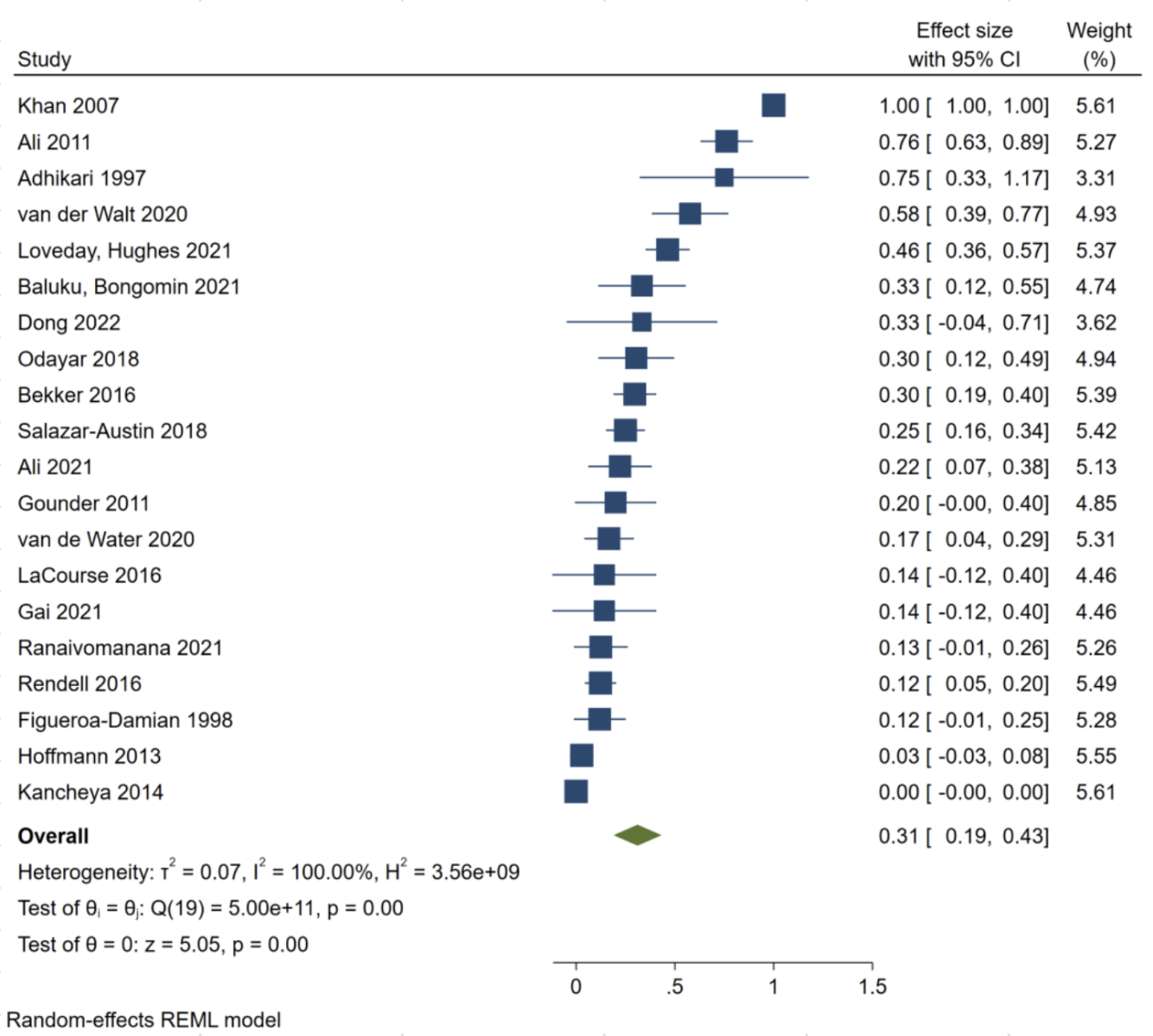


Figure 29. Forest plot of pooled meta-analysis of prevalence of having a prior history of tuberculosis in pregnant and up to six months postpartum women with pulmonary tuberculosis.


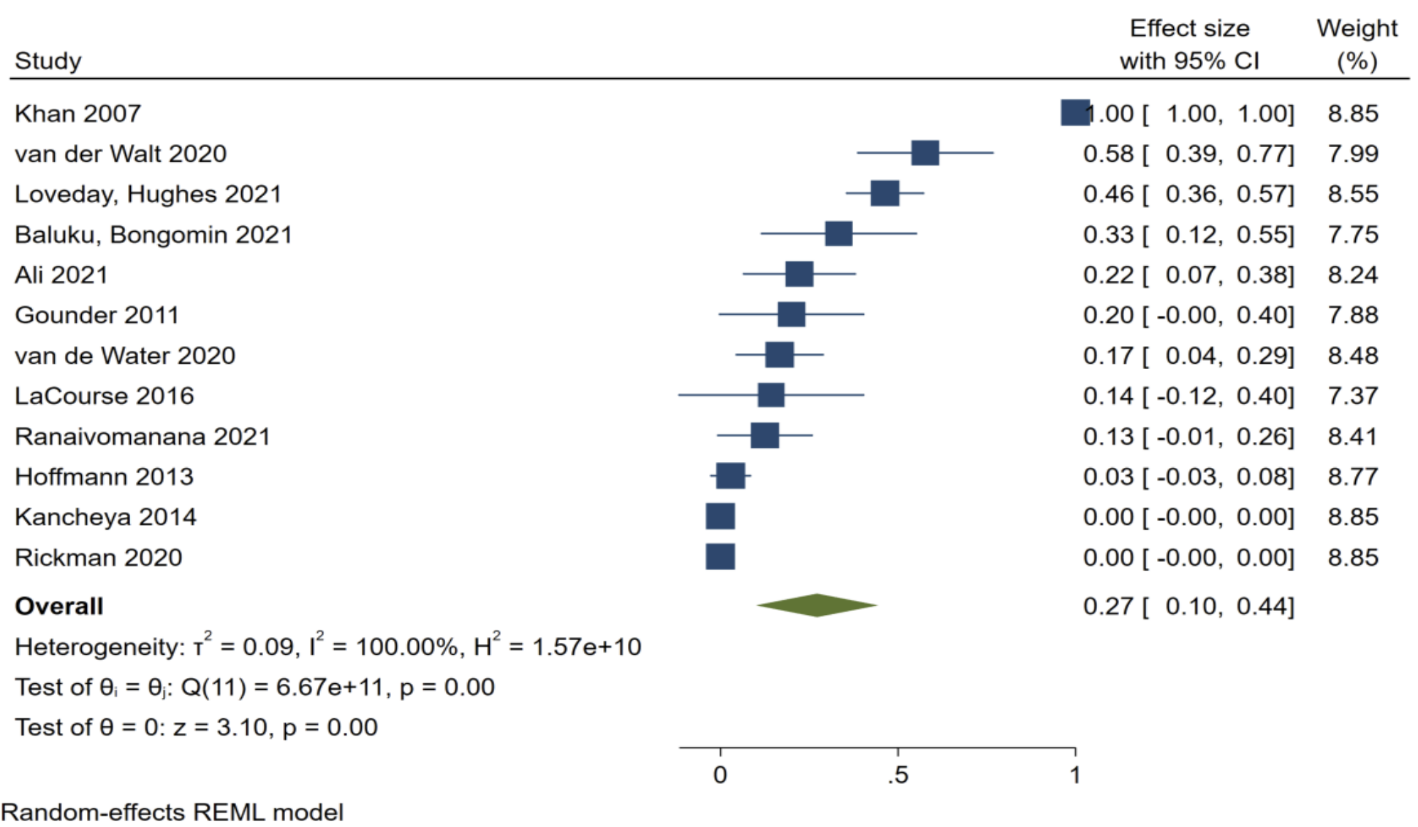


Figure 30. Forest plot of pooled meta-analysis of prevalence of having a history of a known tuberculosis exposure in pregnant and up to six months postpartum women with tuberculosis.


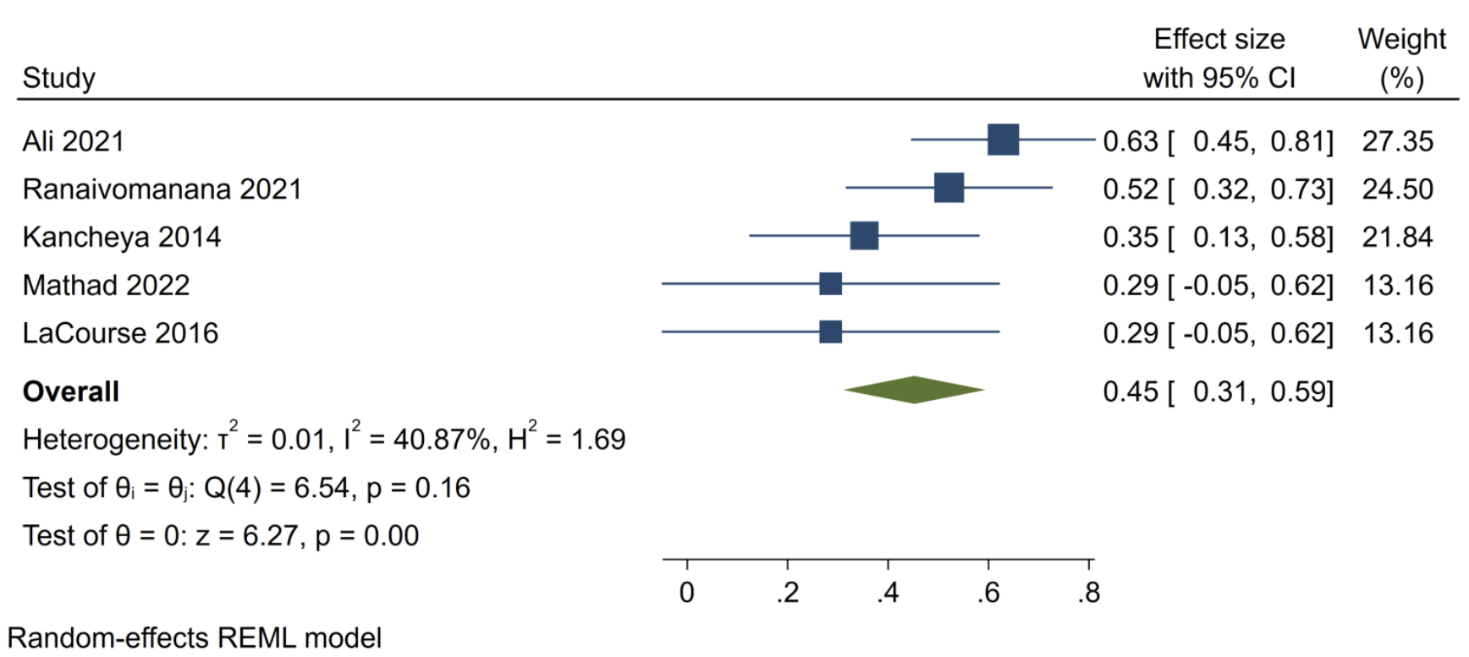


Figure 31. Forest plot of pooled meta-analysis of prevalence of having a history of a known tuberculosis exposure in pregnant and up to six months postpartum women with pulmonary tuberculosis.


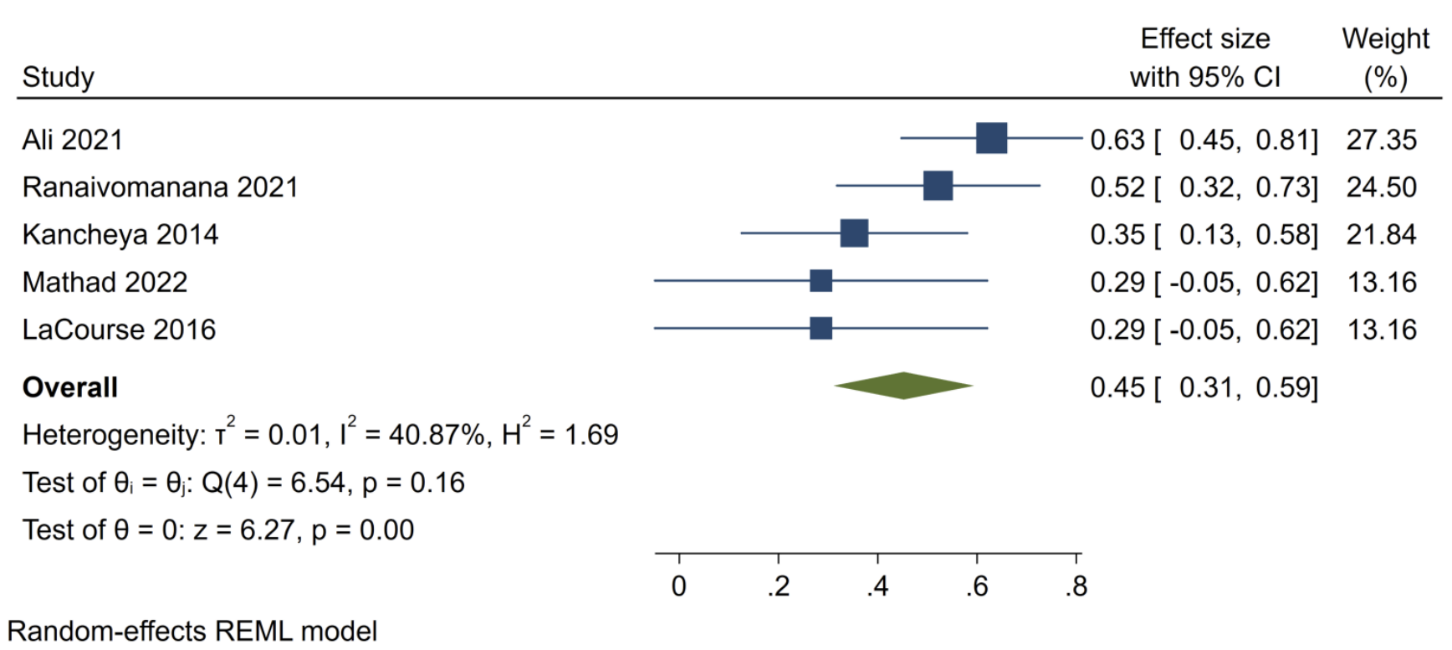


Figure 32. Forest plot of pooled meta-analysis of prevalence of having a history of a close tuberculosis contact in pregnant and up to six months postpartum women with tuberculosis.


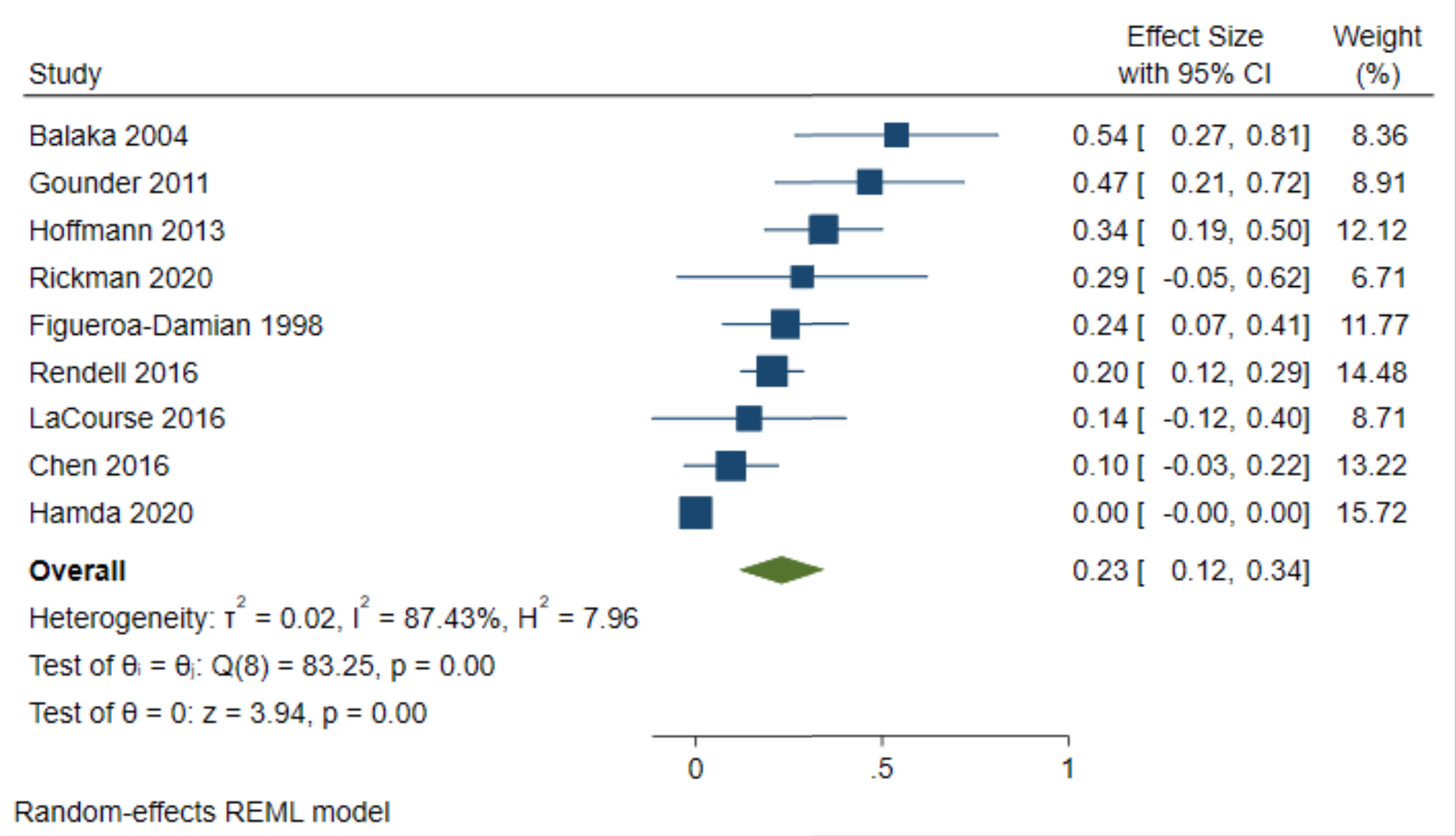


Figure 33. Forest plot of pooled meta-analysis of prevalence of having a history of a close tuberculosis contact in pregnant and up to six months postpartum women with pulmonary tuberculosis.


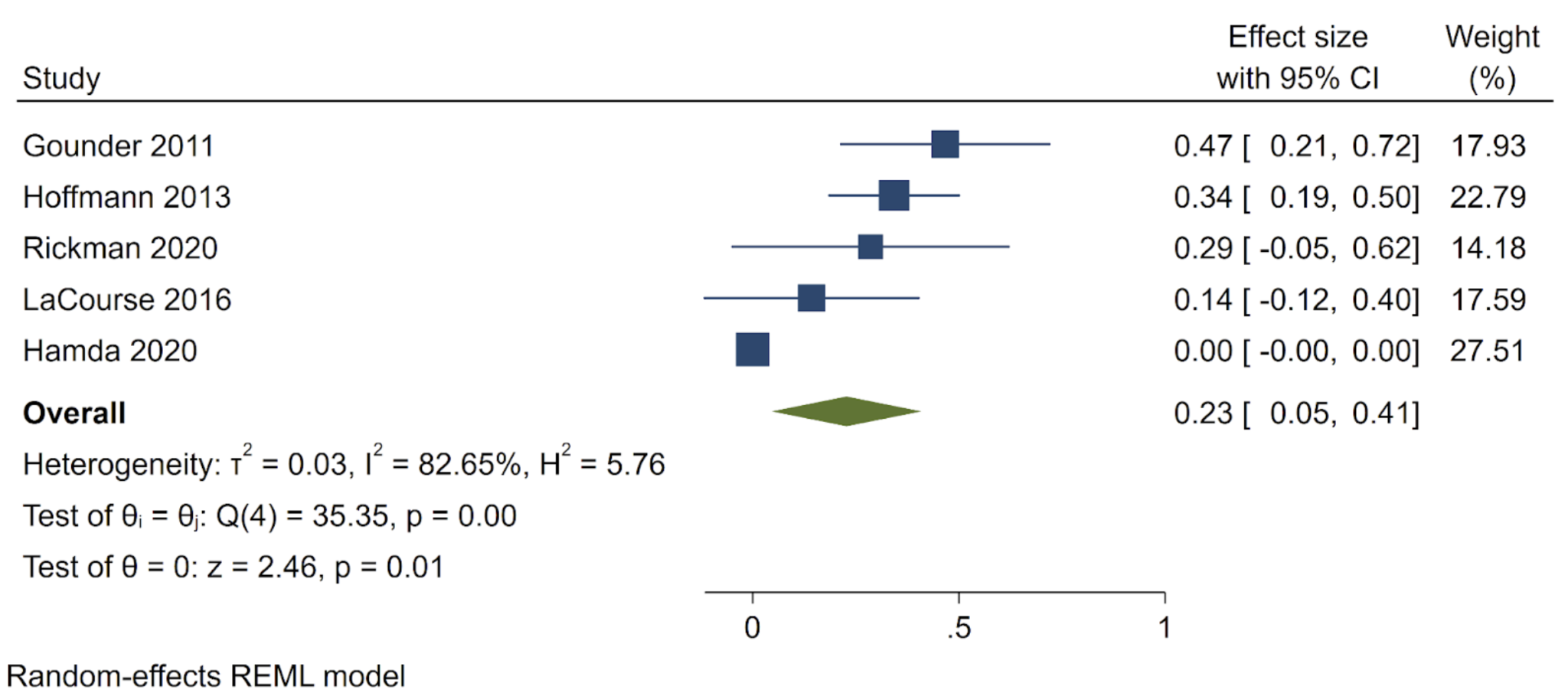

Supplement: S9 File — (DOCX) [file pgph.0002222.s009.docx]
