# Supplementary material for: The clinical presentation and detection of tuberculosis during pregnancy and in the postpartum period in low- and middle-income countries: A systematic review and meta-analysis
Source: PLOS Glob Public Health. 2023 Aug 23;3(8):e0002222. doi: 10.1371/journal.pgph.0002222 (PMC10446195; doi:10.1371/journal.pgph.0002222)

**Appendix S10. Meta-analysis and forest plots for different methods of detecting tuberculosis**

**Symptom screen**. Figure 1a below shows pooled prevalence of women who underwent and were positive on symptom screening. Figure 1b is a subset of these women, including only those women where tuberculosis (TB) diagnosis was unknown prior to testing.

Figure 1a. Forest plot of pooled meta-analysis of the proportion of symptom screened women with symptoms.


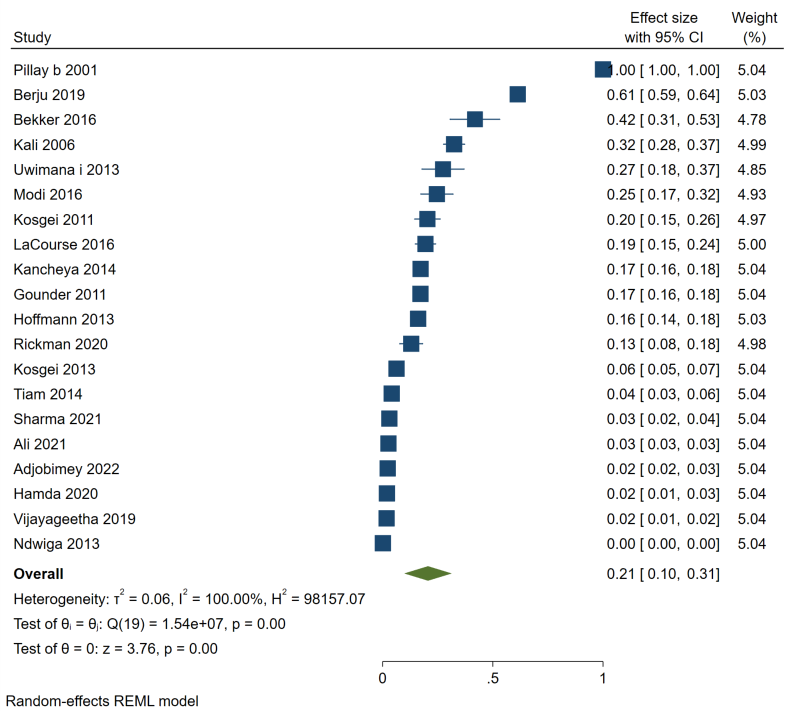


Figure 1b. Forest plot of pooled meta-analysis of the proportion of symptom screened women with symptoms, excluding studies where positive cases were known prior to testing.


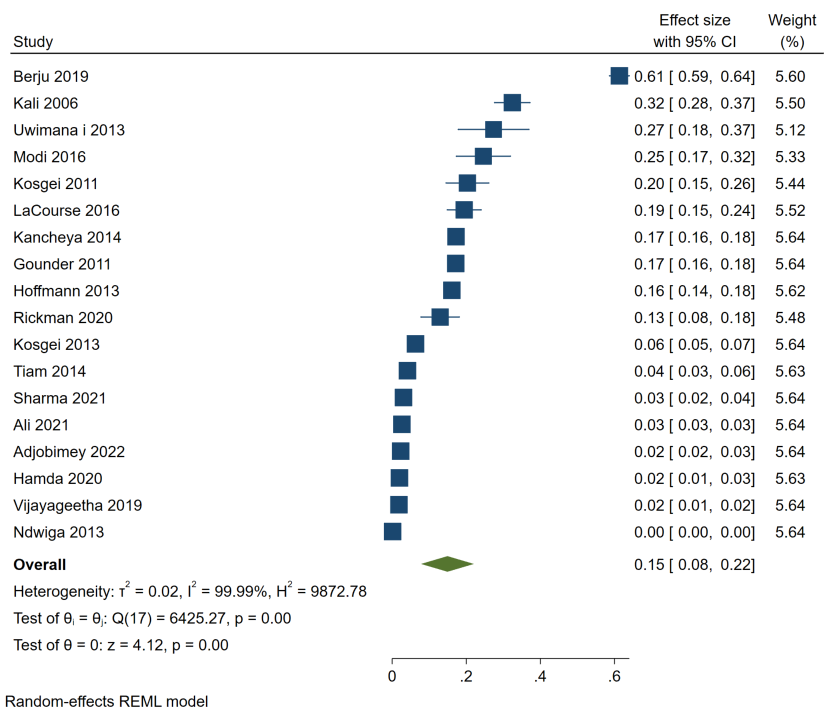


**Chest X-ray**. Figure 2a below shows pooled prevalence of women who underwent and were positive on chest X-ray. Figure 2b is a subset of these women, including only those women where TB diagnosis was unknown prior to testing.

Figure 2a. Forest plot of pooled meta-analysis of the proportion of women given a chest X-ray that have findings suggestive of tuberculosis.


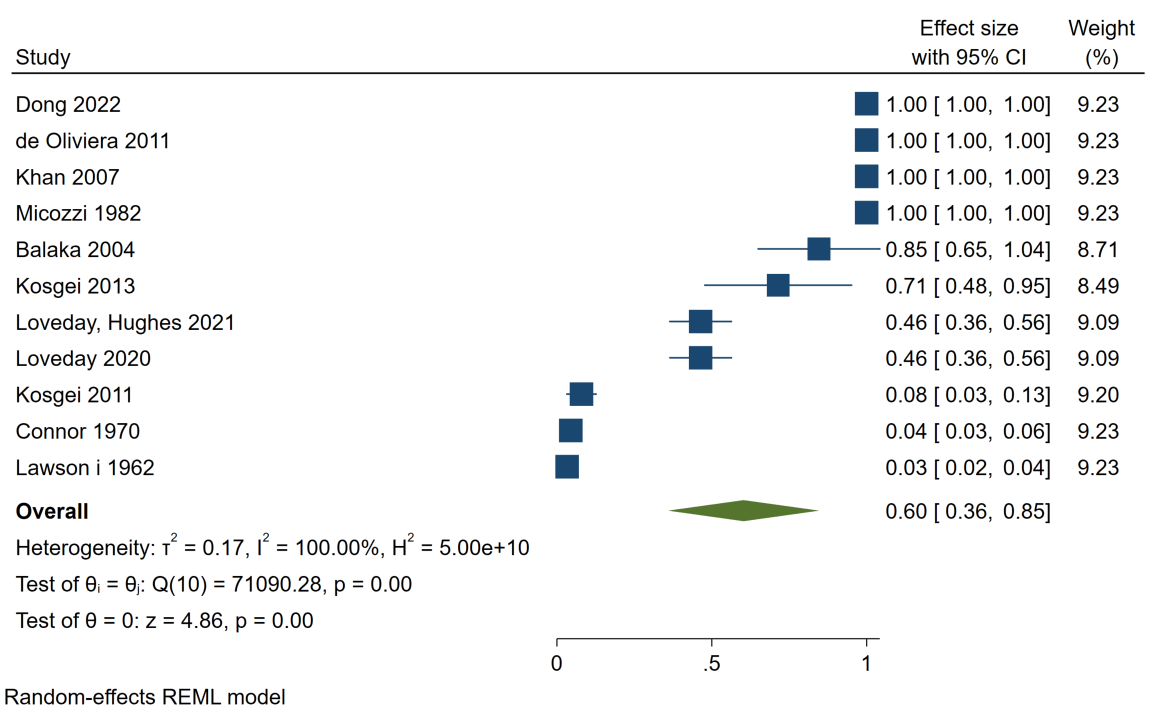


Figure 2b. Forest plot of pooled meta-analysis of the proportion of women given a chest X-ray that have findings suggestive of tuberculosis, excluding studies where positive cases were known prior to testing.


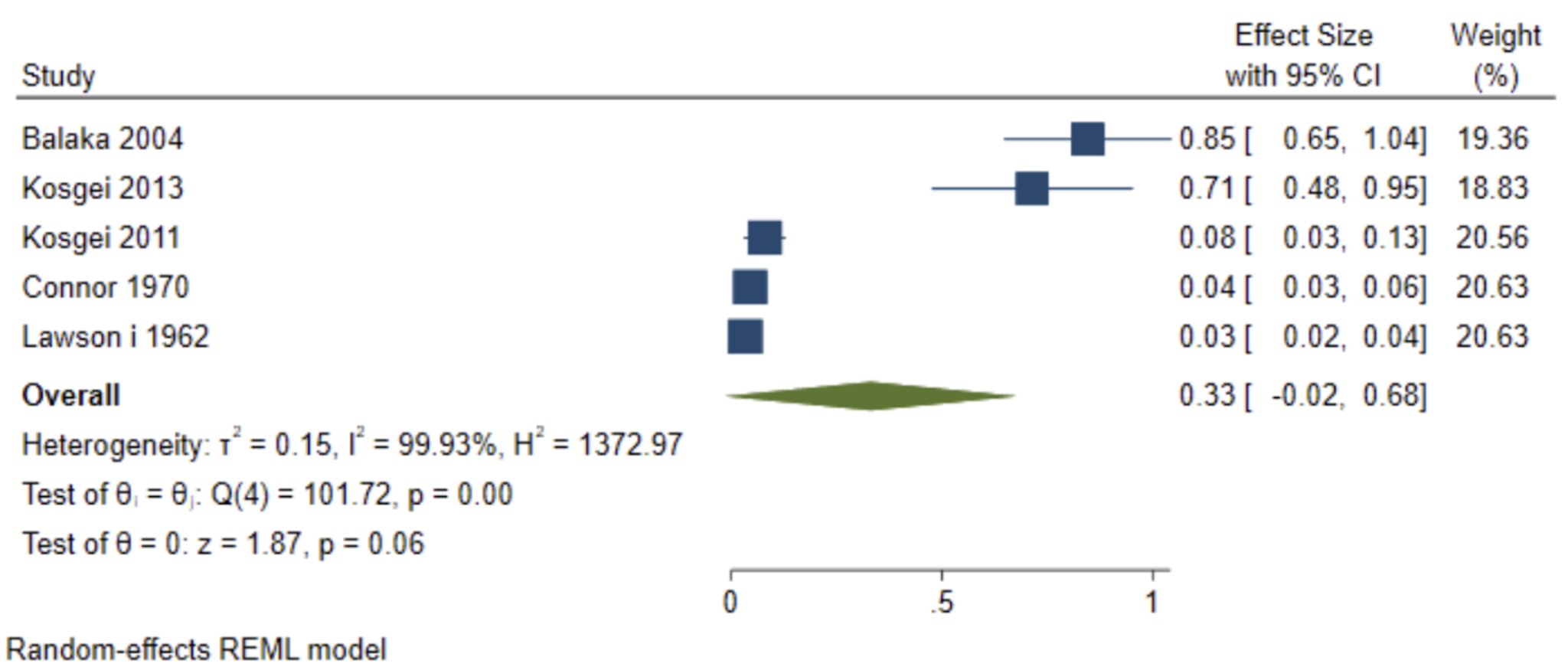


**Sputum smear**. Figure 3a below shows pooled prevalence of women who underwent and were positive on sputum smear. Figure 3b is a subset of these women, including only those women where TB diagnosis was unknown prior to testing.

Figure 3a. Forest plot of pooled meta-analysis of the proportion of women given a sputum smear that have tuberculosis.


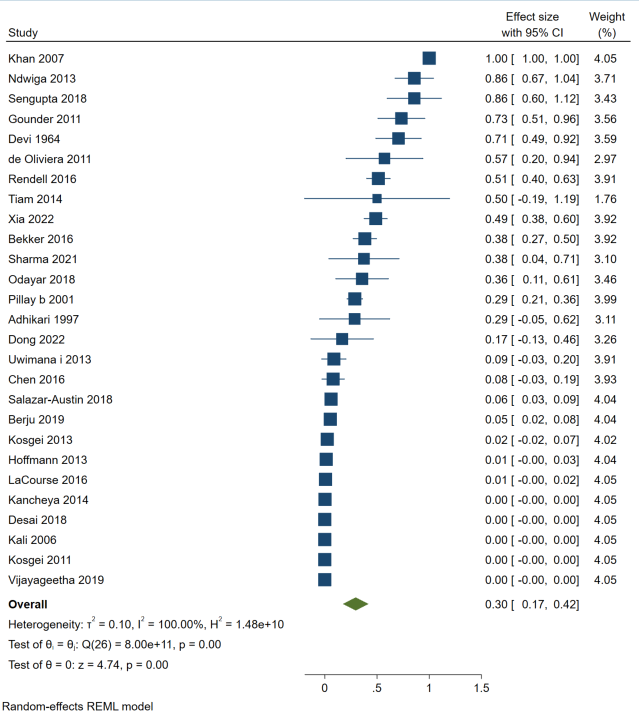


Figure 3b. Forest plot of pooled meta-analysis of the proportion of women given a sputum smear that have tuberculosis, excluding studies where positive cases were known prior to testing.


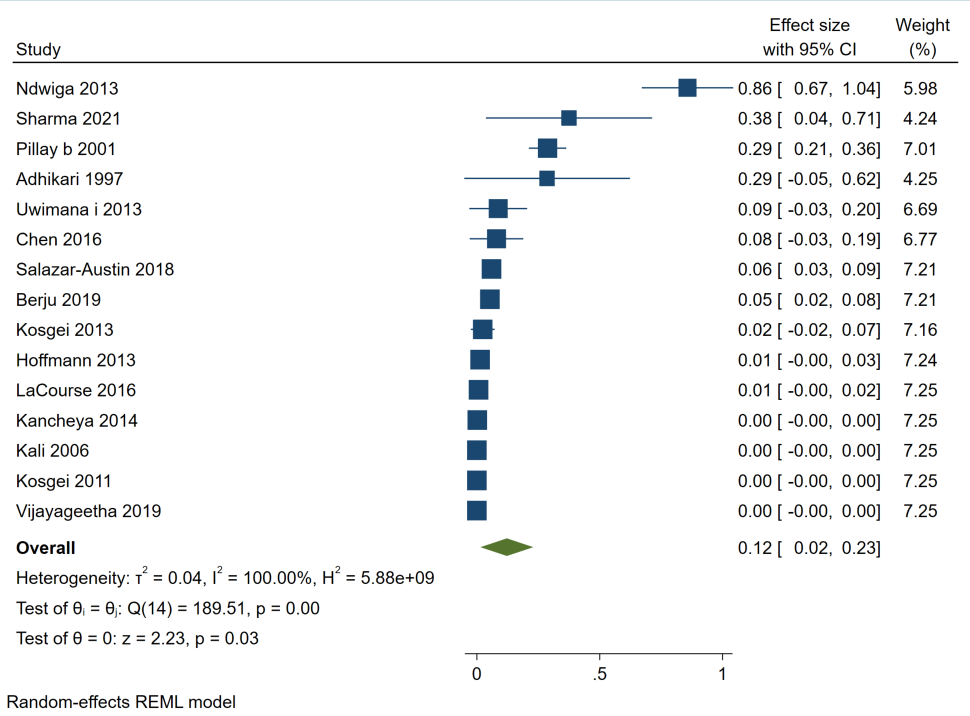


**Sputum Culture**. Figure 4a below shows pooled prevalence of women who underwent and were positive on sputum culture. Figure 4b is a subset of these women, including only those women where TB diagnosis was unknown prior to testing.

Figure 4a. Forest plot of pooled meta-analysis of the proportion of women given a sputum culture that have tuberculosis.


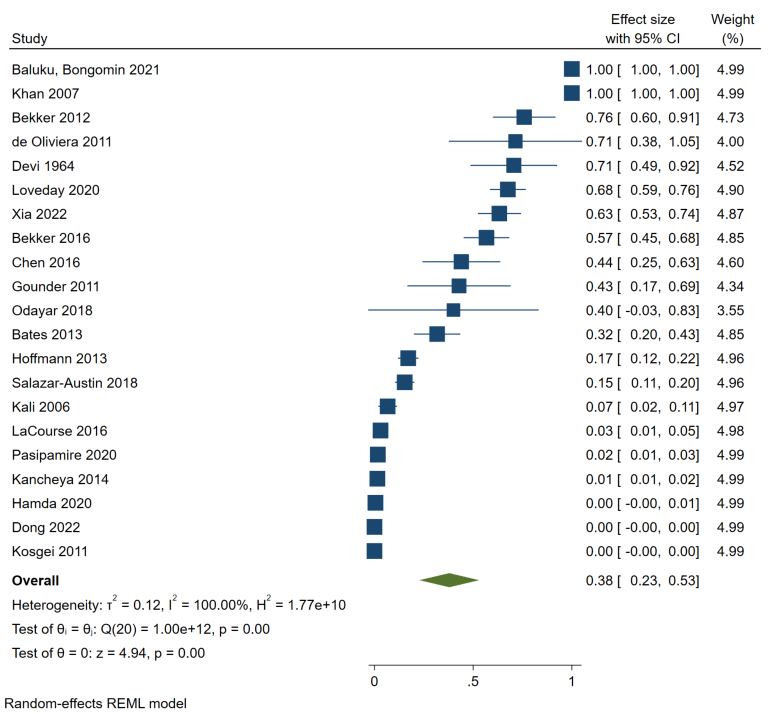


Figure 4b. Forest plot of pooled meta-analysis of the proportion of women given a sputum culture that have tuberculosis, excluding studies where positive cases were known prior to testing.


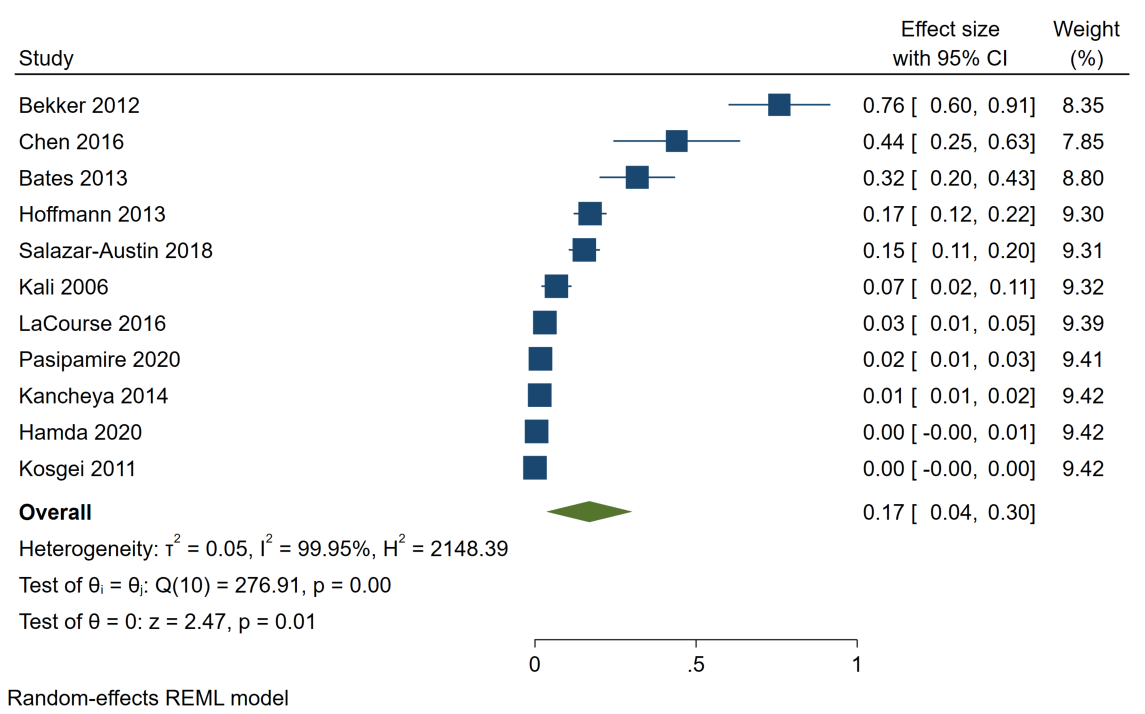


**Polymerase chain reaction (PCR)**. Figure 5a below shows pooled prevalence of women who underwent and were positive on PCR. Figure 5b is a subset of these women, including only those women where TB diagnosis was unknown prior to testing.

Figure 5a. Forest plot of pooled meta-analysis of the proportion of women given a polymerase chain reaction (PCR) test that have tuberculosis.


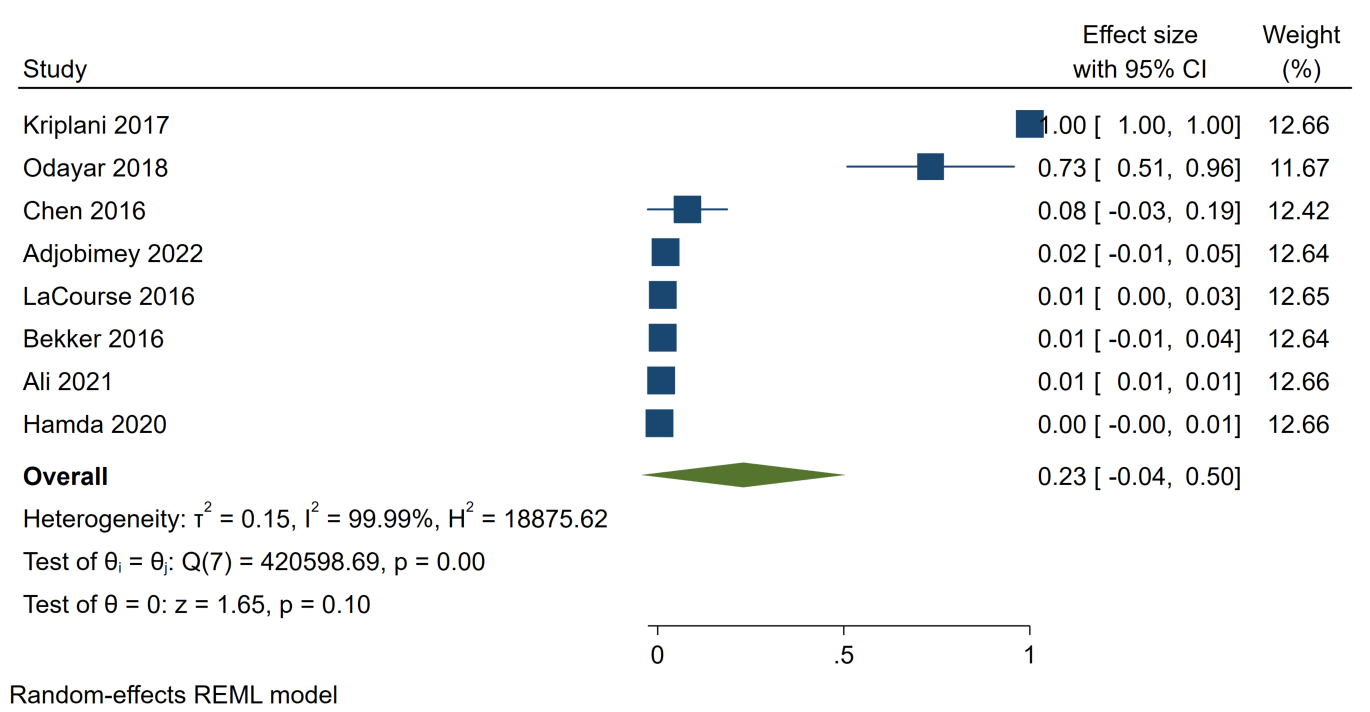


Figure 5b. Forest plot of pooled meta-analysis of the proportion of women given a polymerase chain reaction (PCR) test that have tuberculosis, excluding studies where positive cases were known prior to testing.


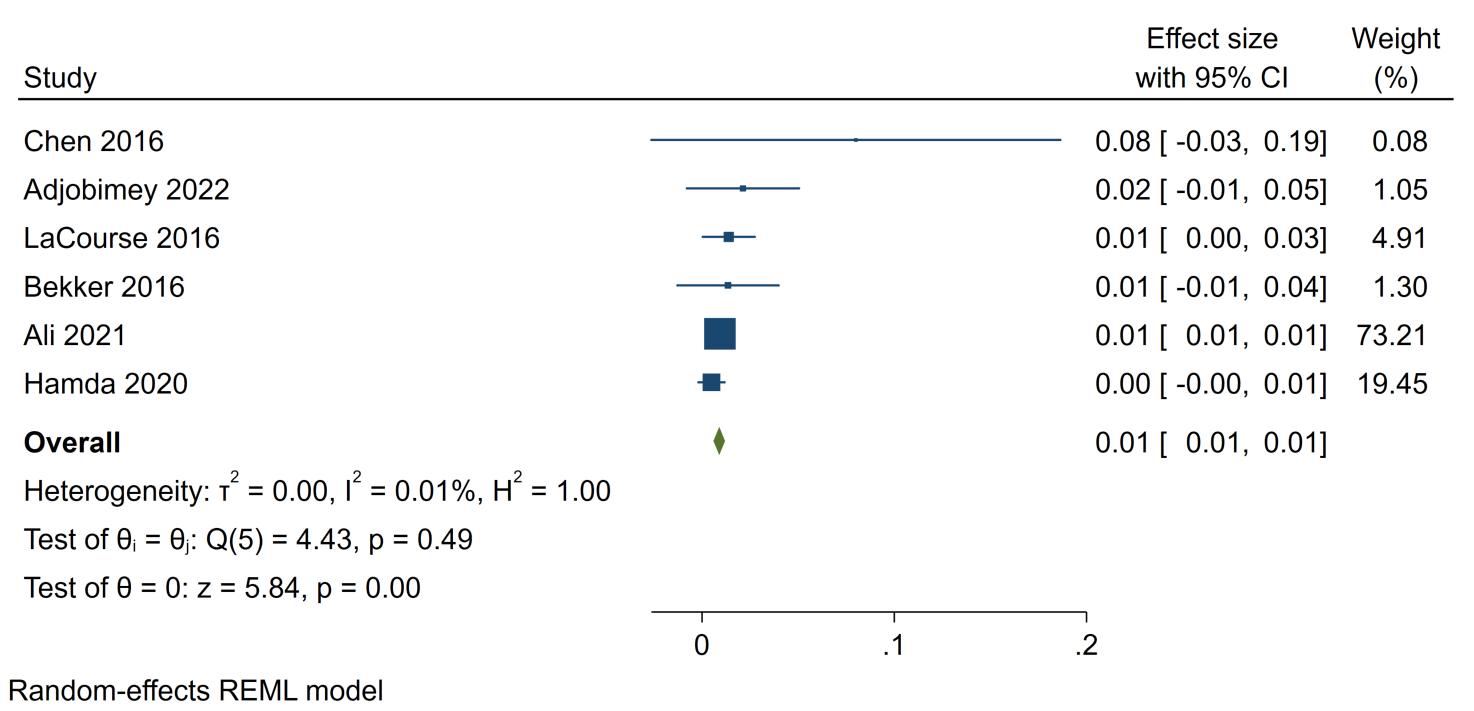


Figure 6. Forest plot of pooled meta-analysis of the proportion of women diagnosed with tuberculosis that are reported to be treated for tuberculosis.


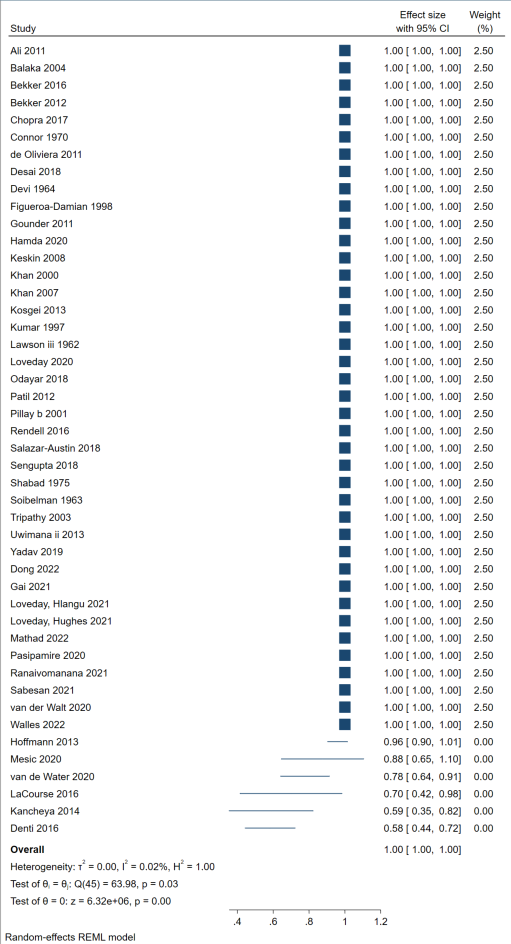

Supplement: S10 File — (DOCX) [file pgph.0002222.s010.docx]
