# Supplementary material for: The clinical presentation and detection of tuberculosis during pregnancy and in the postpartum period in low- and middle-income countries: A systematic review and meta-analysis
Source: PLOS Glob Public Health. 2023 Aug 23;3(8):e0002222. doi: 10.1371/journal.pgph.0002222 (PMC10446195; doi:10.1371/journal.pgph.0002222)
Supplement: S11 File — (DOCX) [file pgph.0002222.s011.docx]

**Appendix S11. Full texts unable to be obtained for analysis.**

1. Anonymous. Millions of women lack maternity care. Safe Motherhood. 1994(14):1-2.
2. Brentlinger PE, Silva W, Valverde E, Buene M, De Morais L, Moon T. Performance of a new guideline for assessment and management of anemia in HIV-infected mozambican adults: Findings from a prospective observational study. American Journal of Tropical Medicine and Hygiene. 2013;89(5):23.
3. Chang FY, Yu MH, Shaio MF. Seroprevalence of human immunodeficiency virus infection in Guinea-Bissau, west Africa. [Chinese]. Zhonghua Minguo wei sheng wu ji mian yi xue za zhi = Chinese journal of microbiology and immunology. 1994;27(2):98-102.
4. Chen YQ, Gao WW. [Progress in therapy of tuberculosis during pregnancy]. Zhonghua Jie He He Hu Xi Za Zhi. 2021;44(5):413-6.
5. D'Cruz IA, Fonseca JM, Parmar VT. Medical causes of maternal mortality. Journal of the Association of Physicians of India. 1968;16(7):417-24.
6. Figueroa Damian R, Arredondo Garcia JL. [Tuberculosis in the pregnant woman]. Ginecologia y Obstetricia de Mexico. 1992;60:209-16.
7. Jedlicka J, Kukralova H. Pulmonary tuberculosis as an indication for interruption of pregnancy. [Czech]. Pracovni Lekarstvi. 1948;28(16):356-60.
8. Kuaban C, Fotsin JG, Koulla-Shiro S, Ekono MR, Hagbe P. Lower lung field tuberculosis in Yaounde, Cameroon. Central African Journal of Medicine. 1996;42(3):62-5.
9. Lorenzo YDJ, Gomensoro Senac J, Gonzalez Abadia M, Gonzalez Panizza V, Lorenzo YDIJ. Blood values in the new-born infants of tuberculous mothers. II. [Spanish]. Archives de Pediatrie. 1950;21(3):185-9.
10. Nedelkov A. Surgical tb in Bulgaria. Bull. 1951;Inst. Microbiol. Acad. Bulgare Sci. 4-5:249-74.
11. Nersieyan RK. THE COURSE of PREGNANCY and LABOR in PATIENTS with RENAL TUBERCULOSIS (Russian). Akuserstvo i ginekologia. 1965;4:31-6.
12. Nguenha D, Acacio S, Murias-Closas A, Ramanlal N, Saavedra B, Karajeanes E, et al. Prevalence and clinical characteristics of pulmonary TB among pregnant and post-partum women. Int J Tuberc Lung Dis. 2022;26(7):641-9.
13. Nogales F, Manglano I. Pathology of cervical polyps. [Spanish]. Acta ginecologica. 1960;11(3):137-51.
14. Pardo IJ, Garcia Galindo G. [Pulmonary resections for tuberculosis during pregnancy; report of the first cases of surgery in Venezuela]. Revista de Obstetricia y Ginecologia de Venezuela. 1955;15(1):515-21.
15. Ragoza VI, Kremer MF. Conduction of labour in patients with active tuberculosis (Russian text). Akush. 1958;I Ginek. 5:60-5.
16. Ranaivomanana P, Knoblauch AM, Razafimahatratra MC, Raherinandrasana AH, Grandjean Lapierre S, Herindrainy P, et al. Pulmonary and pleural TB prevalence in pregnant women. Int J Tuberc Lung Dis. 2021;25(8):668-70.
17. Ryabova MA, Shumilova NA, Lavrova OV, Pestakova LV, Fedotova YS. [Differential diagnostics of the causes responsible for a cough in the pregnant women]. Vestnik Otorinolaringologii. 2016;81(4):50-3.
18. Sadauskas VM, Morkunas AM. Pregnancy and labor in women with a history of pulmonary resection for tuberculosis (Russian). [Russian]. Problemy Tuberkuleza. 1974;52(7):44-6.
19. Schertel L. Patient material at a radiologic department in Western Africa. [German]. Medizinische Klinik (1947). 1971;66(17):652-7.
20. Shah HN, Patel S, Nagpal S. Pelvic inflammation (a study of 800 cases). Journal of Obstetrics & Gynaecology of India. 1978;28(3):429-35.
21. Soibelman LM. The influence of pregnancy, labor, the lactation period, and abortion upon osteoarticular tuberculosis (russian). Voprosy Okhrany Materinstva i Detstva. 1965;11:56-61.
22. Taranenko MI, Ivanov YP. Pregnancy and labor following surgery on the lungs for tuberculosis (Russian). [Russian]. Problemy Tuberkuleza. 1973;51(9):82-3.
23. Urassa E, Massawe S, Mgaya H, Lindmark G, Nystrom L. Female mortality in reproductive ages in Dar es Salaam, Tanzania. East African Medical Journal. 1994;71(4):226-31.
24. Urcuyo Gallegos C, Stein De Guzman MG, Teran Valls M, Rojas A. Preliminary study of severe anaemias of pregnancy. [Spanish]. Revista Medica de Costa Rica. 1950;9(90):37-45.
