# Supplementary material for: The clinical presentation and detection of tuberculosis during pregnancy and in the postpartum period in low- and middle-income countries: A systematic review and meta-analysis
Source: PLOS Glob Public Health. 2023 Aug 23;3(8):e0002222. doi: 10.1371/journal.pgph.0002222 (PMC10446195; doi:10.1371/journal.pgph.0002222)
Supplement: S12 File — (DOCX) [file pgph.0002222.s012.docx]

The clinical presentation and detection of tuberculosis during pregnancy and the postnatal period in low and middle income countries.

**Background**

Tuberculosis (TB) is the major infectious disease globally, causing active disease in an estimated 10 million people and 1.5 million deaths each year (1). An estimated 1.7 billion people or one quarter of the world’s population are infected with *Mycobacterium tuberculosis* and therefore at risk of developing TB (1). For women infected with TB, pregnancy is associated with an increased risk of developing (or worsening) clinical disease (2-4). In TB-endemic countries, it is increasingly recognized that TB is an important cause of maternal and newborn morbidity and mortality associated with an increased risk of preterm birth, low birth weight, and fetal death (5). While congenital TB infection is rare, there is a major risk of mothers infecting their newborns in the postnatal period via airborne spread (6-8). It is therefore imperative that TB is detected and treated during pregnancy or the early postnatal period.

Limited data exist regarding the prevalence of TB in pregnancy. Using population-level data and the incidence of TB, it was estimated that as many as 216,500 women globally experienced TB during pregnancy in 2014 (9). However, the actual number of cases is likely to be much higher - multiple studies have found that pregnancy is an independent risk factor for TB with some showing that there is a 2-3 fold increase in TB during pregnancy and the postnatal period (2-4). The exact mechanisms of this increased risk remain uncertain however proposed mechanisms relate to changes in the immune system during pregnancy. These include pregnancy-related decreases in both TNF-α and proinflammatory T-helper 1 responses that may contribute (3, 10).

Active TB in pregnancy is most prevalent in countries with the highest prevalence of TB infection. Many of these countries are also low and middle income countries (LMICs), defined as those with a gross national income per capita of less than US$12,375, with limited resources for health care and surveillance (11, 12). For countries with a TB prevalence of 100 cases per 100,000 people or greater, the World Health Organization (WHO) suggests screening for active TB in pregnant women as part of routine antenatal care. This may be via standardized symptom screening or chest radiography (13). However, this can be problematic as the symptoms of TB in pregnancy are often difficult to detect. Such symptoms may be either masked, such as with loss of weight, or can be explained by normal physiological changes in pregnancy, such as fatigue, dyspnoea, mild fever and night sweats (7, 14). It is likely that most pregnant women will experience at least one of these symptoms. Given resource limitations, LMICs are unlikely to be able to screen all pregnant women and therefore a successful screening program would require a targeted approach to identify pregnant women at high risk of active TB.

Considering the limited data regarding the clinical presentation of TB during pregnancy or the postnatal period, we aim to conduct a systematic review of the clinical features associated with the presentation and diagnosis of TB during pregnancy in LMICs.

**Objectives**

To identify and summarise the findings of studies regarding the clinical presentation and diagnosis of TB during pregnancy and the postnatal period in low and middle income countries.

**Methods**

Criteria for including studies for this review

*Types of studies*

The studies we will include in this review are peer reviewed studies only, including randomized control trials, non-randomized and quasi randomized studies, cohort, case-control, cross-sectional and descriptive studies. Case reports, letters to the editor, commentaries and conference abstracts will be excluded. We will be following the PRISMA guidelines.

*Types of participants*

In this review, we will include all women in low and middle income countries who are either pregnant or in the postpartum period up to 6 months who are diagnosed with active TB. All types of active TB will be included regardless of method of diagnosis (bacteriologically confirmed or clinically diagnosed) as well as pulmonary or extrapulmonary TB.

*Types of interventions*

We will include all studies regardless of the use of interventions.

*Types of outcome measures*

In this review, we will describe all reported symptoms and signs on the women’s initial presentation and assessment, the demographics of the presenting women and the detection methods for tuberculosis used.

Search methods for identification of studies

*Electronic searches*

This review will require us to search several online databases. The databases used will be Ovid MEDLINE, Embase, CINAHL and Global Index Medicus. The keywords and search strategies used will be altered to suit individual databases. There will be no limitations placed on year of publication or language used in the studies. Included studies will have their reference lists screened for further relevant studies.

Data collection and analysis

*Selection of studies*

Two independent reviewers will independently screen and assess the title and abstracts of the search results and select relevant trials using the inclusion criteria. We will compare these results and where discrepancies are found these will be resolved through discussion between reviewers or consultation with a third reviewer. The same process of assessing eligibility will be conducted for the full texts. Endnote X9 and Covidence online software will facilitate this process. The results of all included and excluded studies will be displayed in a PRISMA flow diagram.

*Data extraction and management*

Data will be extracted from each included study by the primary reviewer and at least one other reviewer and reported in an Excel spreadsheet. The data extracted will include:

- Details of the study
  - Date of publication
  - Type of study conducted
  - Number of participants
  - Country and TB endemicity (high or low)
- Demographics of the study population
  - Age
  - Ethnicity
  - Parity
  - Comorbidities
    - Human immunodeficiency virus (HIV) positive
    - Diabetes mellitus (not including gestational diabetes mellitus)
  - Week of gestation or weeks postpartum at diagnosis
  - TB treatment commenced
- Clinical data
  - Presenting symptoms and signs
  - Onset and duration
  - Detection methods used to diagnose active tuberculosis
  - Bacteriologically confirmed or clinically diagnosed
  - Pulmonary TB or extrapulmonary TB (and type of extrapulmonary TB)
- TB and pregnancy-related outcomes as per standard programmatic definitions

Data extractions will be reviewed for accuracy. Any studies that have discrepancies between the data extractions will be either settled by discussion between reviewers or settled by another reviewer.

For any studies that are not able to be obtained in full text, we will contact the authors to obtain a full text copy. For any trials that are not in English, we will translate the article via Google Translate or consult a native speaker.

*Assessment of study quality*

Two independent reviewers will assess the risk of bias of each included study and any discrepancies found in overall risk category assigned to each study will be settled by discussion between reviewers or a third reviewer.

For all randomized studies included, we will use version 2 of the Cochrane risk of bias tool to assess study quality. The studies will be graded and categorized as being either low risk of bias, some concerns, or high risk of bias.

For all non-randomized studies included, we will use the Newcastle-Ottawa scale to assess study quality. The studies will be graded and categorized as being either good, fair, or poor.

*Data synthesis*

The extracted data will be summarized and reported. If sufficient data is available, we will conduct a meta-analysis to determine the strength of correlation between different presentations and detection methods, and accurate tuberculosis diagnosis.

**Acknowledgements**

Support was provided by the Burnet Institute.

**Contributions of authors**

The idea for the protocol was conceived by Dr. Alyce Wilson, Prof. Steve Graham and Dr. Michelle Scoullar. Grace Simpson drafted the protocol. Dr. Alyce Wilson, Prof. Steve Graham, Dr. Michelle Scoullar and Assoc. Prof. Joshua Vogel reviewed the protocol and provided feedback.

**Declarations of interest**

We declare no conflicts of interest that could influence the study conducted.

**References:**

1. World Health Organization. Global tuberculosis report 2019. 2019.

2. Jonsson J. Tuberculosis control in Sweden. Karolinska University Hospital, Solna 2018.

3. Jonsson J, Kuhlmann-Berenzon S, Berggren I, Bruchfeld J. Increased risk of active tuberculosis during pregnancy and postpartum: a register-based cohort study in Sweden. Eur Respir J. 2020;55(3).

4. Zenner D, Kruijshaar ME, Andrews N, Abubakar I. Risk of tuberculosis in pregnancy: a national, primary care-based cohort and self-controlled case series study. Am J Respir Crit Care Med. 2012;185(7):779-84.

5. Sobhy S, Babiker Z, Zamora J, Khan KS, Kunst H. Maternal and perinatal mortality and morbidity associated with tuberculosis during pregnancy and the postpartum period: a systematic review and meta-analysis. BJOG. 2017;124(5):727-33.

6. Heyns L, Gie RP, Goussard P, Beyers N, Warren RM, Marais BJ. Nosocomial transmission of Mycobacterium tuberculosis in kangaroo mother care units: a risk in tuberculosis-endemic areas. Acta Paediatr. 2006;95(5):535-9.

7. K JS, A B, G KH, S MG. Tuberculosis in pregnant women and neonates: A meta-review of current evidence. Paediatr Respir Rev. 2020.

8. Starke JR. Tuberculosis. An old disease but a new threat to the mother, fetus, and neonate. Clin Perinatol. 1997;24(1):107-27.

9. Sugarman J, Colvin C, Moran AC, Oxlade O. Tuberculosis in pregnancy: an estimate of the global burden of disease. Lancet Glob Health. 2014;2(12):e710-6.

10. Piccinni MP. T cell tolerance towards the fetal allograft. J Reprod Immunol. 2010;85(1):71-5.

11. The World Bank Group. World Bank Country and Lending Groups 2020 [Available from: <https://datahelpdesk.worldbank.org/knowledgebase/articles/906519-world-bank-country-and-lending-groups>.

12. World Health Organization Regional Office for Africa. Tuberculosis 2020 [Available from: <https://www.afro.who.int/news/tuberculosis#:~:text=Over%2095%25%20of%20TB%20deaths,Nigeria%2C%20Pakistan%20and%20South%20Africa>.

13. World Health Organization. WHO recommendations on antenatal care for a positive pregnancy experience. 2016.

14. Nguyen HT, Pandolfini C, Chiodini P, Bonati M. Tuberculosis care for pregnant women: a systematic review. BMC Infect Dis. 2014;14:617.
